# Identification of QTL associated with plant vine characteristics and infection response to late blight, early blight, and Verticillium wilt in a tetraploid potato population derived from late blight-resistant Palisade Russet

**DOI:** 10.3389/fpls.2023.1222596

**Published:** 2023-10-11

**Authors:** Jaebum Park, Vidyasagar Sathuvalli, Solomon Yilma, Jonathan Whitworth, Richard G. Novy

**Affiliations:** ^1^Small Grains and Potato Germplasm Research Station, United States Department of Agriculture – Agricultural Research Service, Aberdeen, ID, United States; ^2^Hermiston Agricultural Research and Extension Center, Oregon State University, Hermiston, OR, United States; ^3^Department of Crop and Soil Science, Oregon State University, Corvallis, OR, United States

**Keywords:** potato late blight (causal agent *Phytophthora infestans*) resistance, early blight resistance, Verticillium wilt resistance, potato vine size, potato vine maturity, tetraploid potato QTL analysis

## Abstract

Potato late blight (causal agent *Phytophthora infestans*) is a disease of potatoes with economic importance worldwide. Control is primarily through field monitoring and the application of fungicides. Control of late blight with fungicides and host plant resistance is difficult, with documented cases of such control measures failing with the advent of new pathotypes of *P. infestans*. To better understand host plant resistance and to develop more durable late blight resistance, Quantitative Trait Locus/Loci (QTL) analysis was conducted on a tetraploid mapping population derived from late blight-resistant potato cultivar Palisade Russet. Additionally, QTL analyses for other traits such as Verticillium wilt and early blight resistance, vine size and maturity were performed to identify a potential relationship between multiple traits and prepare genetic resources for molecular markers useful in breeding programs. For this, one hundred ninety progenies from intercrossing Palisade Russet with a late blight susceptible breeding clone (ND028673B-2Russ) were assessed. Two parents and progenies were evaluated over a two-year period for response to infection by the US-8 genotype of *P. infestans* in inoculated field screenings in Corvallis, Oregon. In Aberdeen, Idaho, the same mapping population was also evaluated for phenotypic response to early blight and Verticillium wilt, and vine size and maturity in a field over a two-year period. After conducting QTL analyses with those collected phenotype data, it was observed that chromosome 5 has a significant QTL for all five traits. Verticillium wilt and vine maturity QTL were also observed on chromosome 1, and vine size QTL was also found on chromosomes 3 and 10. An early blight QTL was also detected on chromosome 2. The QTL identified in this study have the potential for converting into breeder-friendly molecular markers for marker-assisted selection.

## Introduction

*Phytophthora infestans* (Mont) de Bary, causal agent for potato late blight, has detrimentally impacted potato production worldwide (Kassa and Hiskias, 1996; Fry and Goodwin, 1997). During 1840s, *P. infestans* was a major contributing factor to the Irish potato famine (Ristaino, 2002). Today, late blight results in annual global losses of billions of dollars, making it a significant threat to global food security (Latijnhouwers et al., 2004; Haas et al., 2009). Cultural controls such as removing cull piles and volunteer potatoes can reduce pathogen loading (Garrett and Dendy, 2001). Chemical controls such as chlorothalonil, copper oxychloride, dimethomorph, fenamidone + mancozeb, mancozeb, and metalaxyl have been primarily used (Milgroom and Fry, 1998; Khadka et al., 2020). However, the high cost of repeated fungicide application over the growing season to control late blight can significantly impact grower economic returns. This approach is further problematic in areas of the world where fungicides are not readily available or affordable. Guenthner et al. (2001) reported that that the estimated fungicide cost and lost revenue for US growers were $77.1 million and $210.7 million, respectively. Guenther’s average $507 per hectare fungicide cost has increased as the cost of fungicides has increased since 2001. Chemical residue in the crop and the potential of new fungicide-resistant *P. infestans* strains development are additional negative consequences (Milgroom and Fry, 1998; Khadka et al., 2020).

Planting late blight resistant cultivars is an effective and sustainable solution. Multiple potato genetic studies have identified late blight resistance sources, which can be introduced into cultivars of commercial importance. Early potato researchers identified multiple late blight resistance genes (e.g., *R1-R11*) from *Solanum demissum*, a wild hexaploid species indigenous to Mexico. Breeders incorporated these resistance genes into cultivated potato (Black et al., 1953; Malcolmson and Black, 1966; Umaerus and Umaerus, 1994; Chakrabarti et al., 2014; Lindqvist-Kreuze et al., 2021). Resistance derived from *S. demissum* appeared as dominant R genes inducing a hypersensitive response. Each R gene was effective against only a specific race(s) of *P. infestans* indicating vertical resistance (Chakrabarti et al., 2014). *P. infestans* had rapidly evolved to overcome those race-specific R genes through coevolution of matching avirulence. Breeding programs with only one R gene could not successfully generate sustainably resistant clones against *P. infestans* (Chakrabarti et al., 2014). The second concept, quantitative resistance (horizontal or general resistance), compensates for the disadvantage of reliance on a single race-specific resistance gene (van der Plank, 1968). Quantitative resistance typically encompasses several components exerting smaller effects each. These are controlled by the interaction of several genes, providing a more stable host tolerance against various races of *P. infestans* (Graham, 1963; Toxopeus, 1964; Black, 1970; Collins et al., 1999; Costanzo et al., 2004). Finally, pyramiding multiple race-specific R genes is another option to develop clones with more durable resistance. (Tan et al., 2010; Dalton et al., 2013).

Various genetics studies and quantitative trait locus/loci (QTL) analyses have been conducted with wild potato species to achieve pyramiding genes or stable quantitative resistance performance regardless of *P. infestans* races and environmental effects. Since both major and minor late blight resistance sources (e.g., genes or QTL) were observed from diverse wild potato species, such as *Solanum demissum, S. bulbocastanum, S. polyadenium, S. stoloniferum, S. vernei*, and *S. verrucosum*, (Graham, 1963; Toxopeus, 1964; Black, 1970; Khiutti et al., 2015; Karki et al., 2021), various mapping populations were first developed with them, and then analyzed by researchers to localize new resistance genes or QTL. Major and minor late blight resistance QTL were detected across all the 12 fundamental potato chromosomes after inspecting ten different genetic studies conducted with multiple diploid (or di-haploid) bi-parental mapping populations having various wild potato species’ genetic backgrounds (Leonards-Schippers et al., 1992; Leonards-Schippers et al., 1994; van Eck and Jacobsen, 1996; Collins et al., 1999; Visker et al., 2003; Simko et al., 2006; Śliwka et al., 2007; Wickramasinghe et al., 2009; Li et al., 2012; Chakrabarti et al., 2014). Chromosome 5 was most frequently identified as a hotspot for significant QTL relating to *P. infestans* (Leonards-Schippers et al., 1992; Leonards-Schippers et al., 1994; Collins et al., 1999; Visker et al., 2003; Śliwka et al., 2007). Similar QTL analyses were also performed with tetraploid mapping populations, localizing multiple QTL on chromosomes 8 and 9 (Meyer et al., 1998; Massa et al., 2015). The genomic selection study conducted by Enciso-Rodriguez et al. (2018) found that chromosomes 3, 5, 9, 10, and 11 contained several SNPs closely linked to late blight resistance.

In this study, a tetraploid mapping population derived from the hybridization of russet market class parents, was phenotyped for response to pathotype US-8 in Oregon over a two-year period. Additional traits for response to early blight (*Alternaria solani*), Verticillium wilt (*Verticillium dahliae*), and vine size and maturity were characterized during those same years in Idaho. QTL analyses have shown close association of late blight resistance with these four traits (Collins et al., 1999; Hackett et al., 2014; Massa et al., 2018; Odilbekov et al., 2020). Analyses were conducted to determine whether such an association among traits could be found in our tetraploid russet population, and to identify QTL candidates useful for marker-assisted selection (MAS) in potato breeding programs.

## Materials and methods

### Plant material

Palisade Russet is a tetraploid cultivar noted for its resistance to late blight genotype US-8 of *Phytophthora infestans* (Novy et al., 2012), while field assessments indicate the susceptibility of ND028673B-2Russ (Susie Thompson, North Dakota State University, personal communication). Palisade Russet (female parent) was intercrossed with breeding clone ND028673B-2Russ (male parent) in 2008 at Aberdeen, Idaho. The resultant 190 progeny were used as a mapping population with family designation A08241. The primary use of this population was to identify QTL associated with late blight resistance derived from Palisade Russet. Additional traits added to QTL analyses were vine size and maturity, and phenotypic response to early blight and Verticillium wilt.

The late blight-resistant cultivar, Palisade Russet was obtained from the cross between the breeding clone, AWN86514-2 (female) and susceptible breeding clone, A86102-6 (male) with the 4-generation pedigree of Palisade Russet reported by Novy et al. (2012). AWN86514-2 has a complex genetic background, comprised of the potato species *Solanum acaule, S. demissum, S. simplicifolium, S. stoloniferum*, *S. tuberosum* gp Phureja, and *S. tuberosum* gp Andigena (Corsini et al., 1999). The authors postulated that the observed late blight resistance of AWN86514-2 likely was derived from the diversity of species in its pedigree, and this is thought to also be the source of the late blight resistance observed in its progeny, Palisade Russet.

### Late blight resistance field tests in Oregon

US-8, which was one of the strains of late blight, was obtained by Dr. Kenneth Johnson, Department of Botany and Plant Pathology, Oregon State University, and maintained in the potato breeding and genetics program laboratory. The late blight inoculum was increased on modified Rye A media (**Supplementary Material 1**). The Sporangia were harvested by washing the plates with double distilled water. Spore concentration was adjusted to (10^4^ sporangia per mL) by measuring the spore concentration with a hemocytometer. The adjusted sporangia were stored for two hours between 4 and 12 degree Celsius to promote the release of zoospores before field inoculation. Individuals of family A08241 were evaluated for their response to US-8 in inoculated field trials conducted over a two-year period (2019-2020) at Corvallis, Oregon. The experiment was designed as a randomized complete block with two replications of ten-hill plots. The mapping population was planted on 6/20/19 and spreader rows of Ranger Russet and Russet Burbank were sprayed with US-8 spores on 8/30/19 and 9/6/19. The field was irrigated each morning to maintain humidity favorable to late blight spread. Late blight foliage damage was evaluated on September 13^th^, 20^th^, and 27^th^ in 2019. The same procedures were repeated in 2020: planting on 6/24; inoculation on 9/1 and 9/4; foliage damage evaluations on 9/15, 22, and 29; and harvest on 10/22 and 23 of 2020. Late blight field reading scores (1-9 scale) indicated severity of late blight symptoms of each plot (**Supplementary Table 1**). In brief, the higher the number, the more susceptible the individual. After collecting all late blight field scores, an area under disease progress curve (AUDPC) was calculated with the midpoint rule method (Campbell and Madden, 1990).

The AUDPC values were obtained through the following formula:


(eq. 1) $$AUDPC=\sum_{i=1}^{n-1}\left(\frac{\left(t_{i+1}+t_{i}\right)\left(y_{i}+y_{i+1}\right)}{2}\right)$$

Where ***t*** is time in days of each reading, ***y*** is percentage of affected foliage at each reading, and ***n*** is number of readings. Later in this study, two independent QTL analyses were performed for late blight foliage damage reading scores and AUDPC data.

### Idaho field tests for early blight, Verticillium wilt resistance, vine maturity and size evaluations

The A08241 mapping population was also planted in a field at the USDA-ARS Small Grains and Potato Germplasm Research Unit, Aberdeen ID, to allow assessment of foliar early blight and Verticillium wilt responses to infection, as well as vine maturity and size. Naturally occurring *A. solani* and *V. dahliae* in the experiment field were used as inoculums for early blight and Verticillium wilt foliar response tests, respectively. This Idaho field test was executed in 2019-20, as were the late blight evaluations. The Idaho experiment was designed as randomized complete block design with two replications of eight-hill plots. The mapping population was planted on 5/3/19, and four traits were evaluated for a three-day period, 118 days after the planting date. In 2020, the same clones were planted on 5/1, and the four traits were assessed for a three-day period, 117 days after the planting date. A rating scale from 0 (no symptoms) to 9 (>90% of the foliage necrotic) measured mapping population response to infection by early blight and Verticillium wilt (**Supplementary Table 2**). Plant maturity was quantified based on a scale of 1-9 (**Supplementary Table 3**) from 1 (very early: 100% necrosis of vine due to senescence) to 9 (very late: plants are green, in full bloom, and new buds are evident). Vine size was estimated on a scale of 1-9 (**Supplementary Table 4**) with 1 (less than one-foot-tall) to 9 (five-feet-tall or more) when vine is pulled vertically off the ground. All scales used in Idaho field tests had been developed and used over a thirty-year period by potato researchers at the USDA-ARS Small Grains and Potato Germplasm Research Unit. Trained workers cross-checked all phenotype data collected in Idaho to enhance evaluation accuracy. Detailed descriptions of the assessment of the four traits are summarized in **Supplementary Tables 2**–**4**, respectively.

### Best Linear Unbiased Predictors analyses for late blight field reading scores and late blight AUDPC

Before running QTL analyses, all phenotype data were scrutinized through the following mixed-effects model, to calculate estimates of variance components and prediction of genetic values for genotypes (Fernando and Grossman, 1989; Barr et al., 2013; Peixouto et al., 2016):


(eq. 2) $$y_{ijk}=\mu \;+\;G_{i}\;+\;R_{j\left(k\right)}\;+\;Y_{k}+{\left(GY\right)}_{ik}+\;{\text{ε}}_{ijk}$$

Where $y_{ijk}$ is the phenotype of genotype *i* in replication *j* of year *k*, *μ* is population mean, $G_{i}$ is random effect of genotype *i*, $R_{j}$ is random effect of replication *j* within an environment, $Y_{k}$ is fixed effect of year *k*, ${\left(GY\right)}_{ik}$ is genotype *i* by year *k* interaction, and ${\text{ε}}_{ijk}$ is residual error. Each random effect is assumed to be independent from all other random effects, and is normally distributed with a zero mean. Newly obtained prediction for random genotype effects (BLUPs) were used in ensuing QTL analyses (Park et al., 2021). Distribution of all BLUP datasets, non-normality, and data transformation are discussed in the Result and Discussion section below. Exceptionally, the BLUP of the early blight damage phenotype data were not used for the following analyses (e.g., QTL analysis, allele effect analysis, etc.) because almost no segregation was observed in the 2020 phenotype data. Instead, the 2019 raw phenotype data were directly used in those following investigations.

### Statistics for heritability

Broad-sense heritability of each phenotype was computed using following equations (Schmidt et al., 2019).


(eq. 3) $$H^{2}\;=\;\frac{{\text{σ}}_{ɡ}^{2}}{{\text{σ}}_{p}^{2}}$$

(eq. 4) $${\text{σ }}_{p}^{2}\;=\;{\text{σ }}_{ɡ}^{2}+\frac{{\text{σ}}_{ɡy}^{2}}{y}+\frac{{\text{σ}}_{\varepsilon }^{2}}{y\cdot \beta }$$

In equation 3 (eq. 3), $\sigma_{ɡ}^{2}$ and $\sigma_{p}^{2}$ stand for variances of genotypic effect and mean phenotypic measurements across replicates, respectively. In equation 4 (eq. 4), variances of genotypic effect: $G_{i}$ , genotype i by year k interaction effect: ${\left(GY\right)}_{ik}$ , and residual error: ${\text{ε}}_{ijkl}$ are indicated by $\sigma_{ɡ}^{2}$ , $\sigma_{ɡy}^{2}$ , and $\sigma_{\varepsilon }^{2}$ correspondingly. The terms *y* and *β* used in equation 4 (eq. 4) represent the number of years and replications, respectively. JMP Pro^®^ Statistics, Version 12 (SAS Institute Inc., Cary, NC, USA) was used for all statistical analyses and visualization of resulting data (e.g., histograms) discussed in this study.

### Correlation tests between years for each trait and between different traits

Multivariate correlation tests were executed to elucidate similarity across the three BLUP datasets for each trait as well as to look into either positive or negative relationships between two different traits (**Supplementary Tables 5**, **6**). A multivariate function in JMP Pro^®^ Statistics, Version 12 (SAS Institute Inc., Cary, NC, USA) was used to conduct all correlation tests. To discriminate the significance of the p-values of correlation coefficients, a p-value< 0.0001 was selected as the standard. Because only one-year data for the early blight damage was available, the correlation test for the three BLUP datasets within the trait was not performed. Instead, the 2019 raw phenotype data were directly used in the correlation test comparing different traits.

### Genotyping, SNP calling and dosage evaluation

The DNA samples of the mapping population were genotyped with Illumina Infinium SolCAP SNP array version 3 (21,027 SNPs) and Illumina iScan system. Initial DNA sample quality check and acquirement of SNP theta scores were executed by GenomeStudio software (Illumina, Inc., San Diego, CA) as described in Park et al. (2019) and Staaf et al. (2008). Translation from the SNP theta values to autotetraploid marker genotypes (AAAA, AAAB, AABB, ABBB, and BBBB) were carried out with R-package, ClusterCall (version 1.5) (Schmitz-Carley et al., 2017).

### Construction of genetic linkage groups and QTL maps

**Figure 1:**
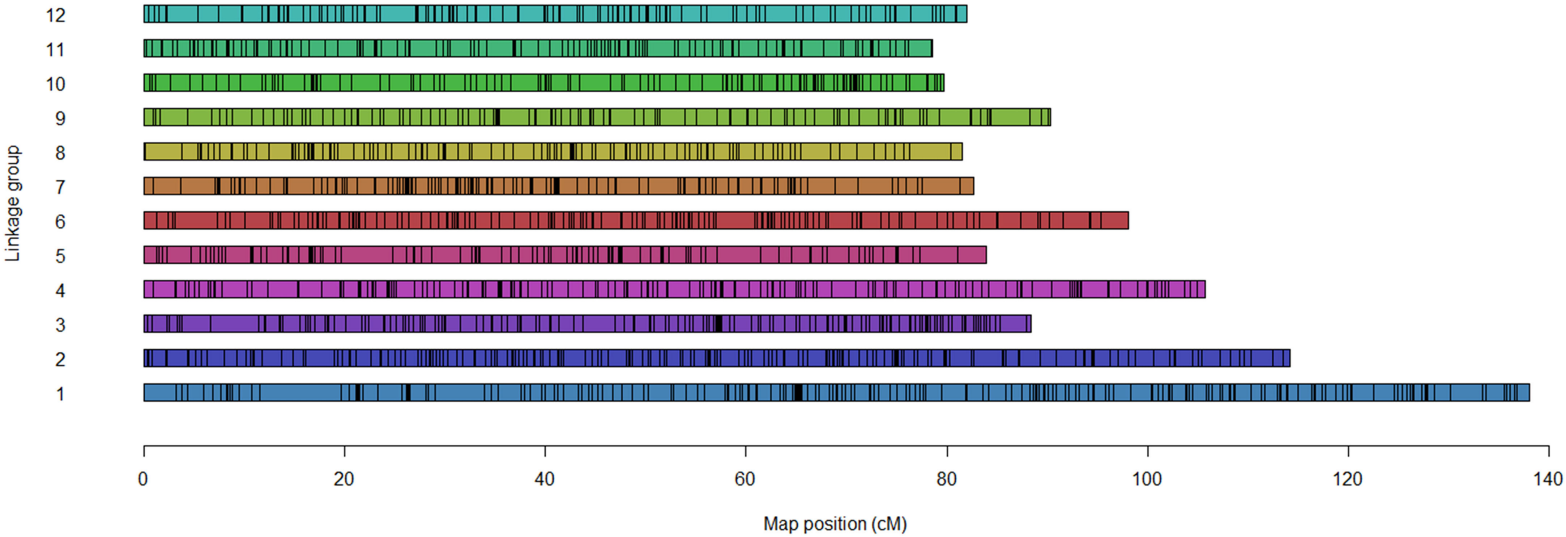
Distribution of the selected 4040 markers across the 12 linkage groups.

MAPpoly software (v. 0.2.3; R-package) constructed overall linkage groups. One strength of MAPpoly is its use in probing polyploids up to octoploid with hidden Markov models (HMM) (Mollinari and Garcia, 2019; Mollinari et al., 2020; R Development Core Team, 2020). Primary uninformative marker filtration processes were conducted through *filter_missing, filter_segregation, make_seq_mappoly*, and *elim.redundant* functions after the translated SNP marker dataset was loaded onto MAPpoly. MAPpoly assembled and refined 12 linkage groups (**Figure 1**; **Supplementary Figures 1**, **2**) based on a mapping pipeline described in Park et al. (2021), using two-point analysis, unweighted pair group method with arithmetic mean (UPGMA) hierarchical clustering, multidimensional scaling (MDS), and potato reference genome PGSC Version 4.03 (Hackett and Luo, 2003; Potato Genome Sequencing Consortium, 2011; Sharma et al., 2013; Hirsch et al., 2014; Preedy and Hackett, 2016; da Silva Pereira et al., 2020; Mollinari et al., 2020; Spud Database, 2020).

QTL mapping was also automated by QTLpoly (R-package), which can run QTL mapping processes of polyploid organisms. The 12 linkage groups and all phenotypic data, converted to BLUP (**Supplementary Figure 3**), were loaded on QTLpoly to construct 12 QTL maps, based on the instruction of da Silva Pereira et al. (2020). Exceptionally, only the 2019 raw early blight damage phenotype dataset was employed to localize early blight resistance QTL. Concisely, among the diverse genetic analysis arguments embedded in QTLpoly, the *remim* function, which carried out a random-effect multiple interval mapping (REMIM) model, was chosen for fitting various random-effect QTL by evaluating a single parameter per QTL. The QTLpoly software then ran linear score statistics tests (Qu et al., 2013) at every position and compared its p-value to a prescribed critical value. The p-values appeared as a continuous pattern over the whole range of the unit interval as a result of weighted sums of the scores from the profiled likelihood (Qu et al., 2013; da Silva Pereira et al., 2020). QTLpoly conducted conversion of the p-values to LOP scores (LOP = – log10 (p-value)) to visualize and compare newly detected QTL in this study intuitively as well as to estimate support intervals of those QTL. Based on the QTLpoly software developer’s recommendation, the QTL with four or higher LOP scores were adopted as significant QTL peaks (da Silva Pereira et al., 2020). Besides, this software also presented support intervals defined as the QTL peak adjacent to zone with LOP higher than or equal to LOP – d, where d is a constant, which subtracts the highest LOP in that region (Lander and Green, 1987; da Silva Pereira et al., 2020). Approximately 95% support intervals were selected for this study and calculated by using LOP – 1.5 (da Silva Pereira et al., 2020). Moreover, QTLpoly can compute the heritability of the significant QTL using the *fit_model* argument (da Silva Pereira et al., 2020). Those QTL heritability values were reported with the symbol “h^2^_QTL_.” This QTL heritability (h^2^_QTL_) should be distinguished from the general heritability (e.g., Broad-sense heritability), which represents how well a trait was inheritable from two parents to their progeny. The h^2^_QTL_ with over 10% was considered a major QTL, while another h^2^_QTL_ ≤ 10% was considered a minor QTL as the software inventor did before (da Silva Pereira et al., 2020). In addition, other information, such as the closest SNPs linked to significant QTL, allele effects, etc., were intensely inspected after localizing significant QTL.

### Allelic effect analyses

The *qtl_effects* function of QTLpoly developed bar graphs of the allelic effects at each QTL position (**Supplementary Figure 4**; **Supplementary Table 7**). The four homologs of both parents were indicated on the X-axis of an allele effect graph. For instance, “a-d” stand for four Palisade Russet homologs, and “e-h” represent another four homologs of the ND028673B-2Russ. The quantity of an allele effect of each homolog was depicted on Y-axis (**Supplementary Figure 4**). The bar graphs efficiently present parental contributions to the average of the whole mapping population. These indicate how much each homolog of each parent adds to or subtracts from the mean of the 190 progenies (da Silva Pereira et al., 2020), revealing which allele(s) among the eight parental homologs most significantly impact a trait. The vector, the quantity of either positive (= increase in) or negative (= decrease in), of each allele was converted to absolute value to compare allele effects. For example, the sum of all the eight absolute values at each mapped locus was used to quantify the influence of the mapped QTL. The sum of the four absolute values of each parent was also utilized to compare the contribution of each parent to a trait. (G. da Silva Pereira, unpublished).

### Haplotype comparison between late blight resistant and susceptible clones

The haplotypes of each individual were visualized by the *calc_homoprob* function of MAPpoly, which were then used for the linkage mapping process (Mollinari et al., 2020). The haplotype images revealed regions of cross-over, where inversion of probability magnitudes between homologs from same parent took place. The haplotype comparison analysis could display haplotype differences between late blight resistant and susceptible groups. Since the major interest of this study was localization of late blight resistance, that haplotype comparison analysis was conducted to reinforce reliability of late blight QTL detected in this study.

For haplotype comparison, resistant clones consistently ranked in the lowest 20% across two late blight AUDPC (LB-AUDPC) year datasets, were selected as a late blight resistant panel. Likewise the highest 20% of the most susceptible clones were chosen as a susceptible panel. After collecting all the haplotype images of those selected clones, the place where the significant QTL for the late blight resistance was identified was intensively investigated to confirm the presence of a resistant allele on an appropriate homolog and position in the resistant clones or vice versa (In other words, the absence of the resistant allele in the susceptible clones). The feature of the resistant haplotype comparison provided helpful information for future MAS.

## Results

### Marker selection and linkage group construction

A total of 8222 tetraploid markers were selected and translated by ClusterCall into readable tetraploid genotypes. Forty-three SNPs having no-call in either of two parents were omitted, as they could not contribute to linkage groups. Since the accurate chromosome numbers for 61 SNPs were not available in the potato reference genome PGSC Version 4.03, those SNPs were omitted to avoid potential errors. Nine SNPs tagged as having incorrect physical map location information were removed to avoid extending the length of a linkage group (**Supplementary Material 2**). Additional marker filtration was run on the remaining 8,109 markers using MAPpoly software. When markers were loaded on the *read_geno_csv* function, 3315 non-conforming and redundant markers further were eliminated. One hundred fifteen SNPs with 5% or more no-calls were filtered by the *filter_missing* argument. The *filter_segregation* function conducted the chi-squared (χ2) test, which matches expected genotype frequencies against observed frequencies, resulting in the associated p-value. Informative SNPs were distinguished by the Bonferroni correction (p-value< 0.05). The *make_seq_mappoly* argument excluded an additional 162 SNP markers, which did not meet expected segregation ratios based on Mendelian inheritance. Finally, 477 markers, which were uninformative, co-segregating, or not belonging to one of 12 linkage groups were removed during two-point and MDS processes in MAPpoly.

A total of 4040 informative SNP markers were selected to develop 12 linkage groups identical to the underlying potato chromosome number (**Table 1**). Selected markers were uniformly allocated on each chromosome without wide gaps between contiguous SNPs (**Figure 1**). The number of SNP markers used to develop each linkage group ranged from 404 for chromosome 1 to 201 for each of chromosomes 10 and 12 (**Table 1**). In Palisade Russet and ND028673B-2Russ, 3217 and 3153 markers segregated, respectively. Among the 12 linkage groups, chromosome 1 was longest [138.02 centiMorgan (cM)], while chromosome 11 was shortest (78.53 cM). The majority of linkage groups showed almost 100% coverage of the potato physical map, excepting chromosome 7, which has a 94% map coverage rate (**Table 1**). Average distance between contiguous SNPs for the two parents was 0.35 cM. Comparison of SNP marker positions of the linkage groups to PGSC version 4.03 physical maps confirmed a high concordance (**Supplementary Figure 1**). **Supplementary Figure 2** includes all visualized 12 complete autotetraploid linkage groups for each parent.

**Table 1:** Linkage group summary for Palisade Russet and ND028673B-2Russ.

	No. Mapped SNPs ^a^			Map Length (cM) ^b^			Map Coverage ^c^	
Chr ^d^	Total	Palisade Russet	ND028673 B-2Russ	Palisade Russet	ND028673 B-2Russ		Palisade Russet	ND028673 B-2Russ
1	505	362	404	138.01	138.02		1	1
2	454	371	353	114.15	114.15		1	1
3	384	344	317	88.44	88.44		1	1
4	384	275	300	105.75	105.75		1	1
5	296	215	217	83.93	83.93		1	1
6	314	247	244	98.04	98.04		1	1
7	294	242	218	82.64	68.83		1	0.94
8	317	262	240	81.58	76.28		1	0.98
9	273	203	228	90.38	90.38		1	1
10	258	231	201	79.73	79.73		1	1
11	318	259	230	78.53	78.53		1	1
12	243	206	201	80.99	82.03		0.99	1
Total	4040	3217	3153	1122.17	1104.11		1	0.99

a The number of mapped single nucleotide polymorphisms.

b Linkage group lengths in centiMorgans (cM).

c Map coverage relative to PGSC Version 4.03 pseudomolecules.

d Chromosome number.

### Summary of segregation pattern, heritability, and BLUP datasets of collected phenotype data

#### Late blight foliage damage and LB-AUDPC

No late blight scores of “1” (no symptom of infection) or “2” (more than 0% but less than 10%) were observed in late blight foliage damage records across three reading points, two years, and two replications. This indicates that no individuals in the population displayed an immune or highly resistant response to late blight (**Supplementary Material 3**). Late blight foliage damage scores taken at final field reading reflected scores of “3” (up to 10% of foliage expressing late blight symptoms) to “9” (completely destroyed foliage). The only exception was clone A08241-12 (scored as a “2” in 2020) (**Supplementary Material 3**). The third field ratings for resistant parent Palisade Russet were “4” in 2019 and “3” in 2020, respectively. Those of ND028673B-2Russ were consistently “9” across two years, reflecting its susceptibility to infection by late blight. Ratings of late blight infection in the population taken at the third evaluation in each year (September 27^th^, 2019 & September 27^th^, 2020) were used for the QTL analysis. LB-AUDPC values for each clone were calculated by LB-AUDPC equation 1 (eq. 1), based on raw late blight foliage damage data (**Supplementary Material 3**). LB-AUDPC values were distributed from 97 to 2188 in 2019 and 66.5 to 1400 in 2020. LB-AUDPC values of Palisade Russet averaged 191 in 2019 and 115.5 in 2020, respectively. Those of ND028673B-2Russ were 2081.5 in 2019 and 1366.8 in 2020, respectively. Third reading points of late blight foliage damage and LB-AUDPC data were then run through the mixed-effects model (eq. 2), resulting in variance component estimates and BLUP values of the two traits. **Table 2** summarizes variance component estimates of late blight foliage damage and LB-AUDPC. When variance components of three random effects and the residual of late blight foliage damage were compared, clone and clone × year effects accounted for approximately 93% of the variance components of late blight foliage damage. Likewise, clone and clone × year effects accounted for about 92% of the variance components of LB-AUDPC (**Table 2**). Broad-sense heritability of late blight foliage damage and LB-AUDPC were 0.71 and 0.66, respectively. Based on these results, genetic and G × E effects were the primary contributors to variance in foliar late blight response to US-8 observed in the mapping population.

**Table 2:** Variance component estimates of late blight foliage damage, LB-AUDPC, Verticillium wilt foliage damage, vine maturity, and size.

Late blight foliage damage (The third reading point only)			Area Under the Disease Progress Curve (LB-AUDPC)		
Random Effect	Var ^a^ Component	Std Error ^b^	Random Effect	Var ^a^ Component	Std Error ^b^
clone ^c^	2.1306651	0.3184543	clone ^c^	138439.75	22678.298
rep[year]	0.010144	0.0116243	rep[year]	260.1653	384.04509
clone*year	1.5782682	0.1763395	clone*year	130865.87	14632.559
Residual	0.2841269	0.0205587	Residual	23764.867	1719.5654
Total	4.0032041	0.3188287	Total	293330.66	22697.5
Fixed Effect	Estimate	Std Error ^b^	Fixed Effect	Estimate	Std Error ^b^
Intercept	7.1276042	0.134585	Intercept	1165.2826	34.02678
year[2019]	0.3958333	0.083762	year[2019]	358.07682	20.89932
Verticillium wilt foliage damage					
Random Effect	Var ^a^ Component	Std Error ^b^			
clone ^c^	1.238304	0.151373			
rep[year]	0.002336	0.006218			
clone*year	0.046156	0.04973			
Residual	0.715723	0.052804			
Total	2.002519	0.153703			
Fixed Effect	Estimate	Std Error ^b^			
Intercept	2.724957	0.091294			
year[2019]	1.001949	0.040953			
Vine maturity			Vine size		
Random Effect	Var ^a^ Component	Std Error ^b^	Random Effect	Var ^a^ Component	Std Error ^b^
clone ^c^	1.584096	0.182213	clone ^c^	0.655115	0.082612
rep[year]	0.002099	0.004952	rep[year]	3.99E-05	0.001504
clone*year	0.054202	0.03847	clone*year	0.121411	0.028914
Residual	0.525865	0.03883	Residual	0.269235	0.019999
Total	2.166261	0.183208	Total	1.0458	0.083182
Fixed Effect	Estimate	Std Error ^b^	Fixed Effect	Estimate	Std Error ^b^
Intercept	4.878051	0.099494	Intercept	4.461094	0.064988
year[2019]	-0.48061	0.037182	year[2019]	-0.21075	0.026481

a Variance component.

b Standard error.

c “clone” indicates the genetic effect of a clone.

Late blight foliage damage (the third reading point) produced three BLUP datasets and LB-AUDPC data, depending on the combination of BLUP effects of each clone. The first BLUP dataset, “*LB_clo*” is BLUPs of pooled phenotypic data across both years, with “*LB*” being an abbreviation for late blight foliage damage. The second set, “*LB_clo_2019*,” had BLUPs of the interaction between the clone and the 2019-year effect. Likewise, the third set, “*LB_clo_2020*,” included BLUPs of the interaction between the clone and the 2020-year effect. Another three BLUP datasets (*LB-AUDPC_clo, LB-AUDPC _clo_2019*, and *LB-AUDPC _clo_20202*) were obtained in the same manner. Each BLUP dataset mentioned above was composed of 190 BLUPs equal to the progenies number used for the linkage mapping process. A detailed description of BLUP datasets is summarized in **Supplementary Table 5**. All six BLUP datasets skewed to one side (**Supplementary Figure 3**), but the skew did not affect QTL results. Additional data transformation tests and supporting evidence will be briefly discussed in the Discussion section below to show that observed non-normality of BLUPs did not impact QTL analysis reported in this study.

#### Vine maturity, vine size, Verticillium wilt damage and early blight foliage damage

Vine maturity evaluation scores of the A08241 population were distributed from “1” to “8” in 2019 and from “2” to “9” in 2020, respectively (**Supplementary Material 3**). Maturity was predominantly affected by each clone’s genetic background; the variance component of clone effect accounted for over 73% of total variance components of vine maturity (**Table 2**). Broad-sense heritability of maturity was 0.91. Vine size scores ranged from “2” to “8” in 2019 and from “3” to “8” in 2020 (**Supplementary Material 3**). Similarly, vine size was mainly affected by clone effect, accounting for over 63% of the total variance components. Broad-sense heritability of vine size was 0.84.

In the Verticillium wilt resistance evaluation, progenies of the A08241 population scored from “0” to “9” in 2019, although no progeny scored “9” in 2020 (**Supplementary Material 3**). Clone effect (62%) was significantly greater than the other components of Verticillium wilt resistance, indicating a strong association of trait with genetic impact for each clone (**Table 2**). The resistance trait’s broad-sense heritability was 0.86.

The mixed-effects model (eq. 2) generated three BLUP datasets for each trait except early blight data, which was omitted from the 2020-year data. The same naming system as introduced in late blight foliage damage (*LB*) and LB-AUDPC (*LB-AUDPC*) BLUPs above was used, with *“VM,” “VS,”* and *“VW”* abbreviating vine maturity, vine size, and Verticillium wilt damage, respectively. See **Supplementary Table 5** for a detailed description with total BLUP numbers for each dataset. Although 190 progeny were used for genetic mapping, some BLUP datasets had fewer than 190 BLUPs because several clones in the population had poor field emergence or did not grow well during the growing seasons (**Supplementary Material 3**; **Supplementary Table 5**).

Unlike the other five characterized traits above, minimal segregation was observed for early blight in 2020 in the mapping population, with individuals displaying no or very few early blight symptoms of the foliage during the growing season (**Supplementary Material 3**). Our early blight resistance test relied on a naturally occurring *Alternaria solani* in the research field; thus, the amount of inoculum may have been insufficient in 2020 to induce effective segregation for QTL analysis, resulting in no meaningful segregation and no significant QTL from 2020 early blight data. Therefore, the calculation of broad-sense heritability for early blight resistance, conversion from raw phenotype data to BLUP, correlation test between the two year phenotype datasets, and QTL analysis with BLUP datasets were not discussed for early blight resistance data. Instead, the average of two replicates of 2019 raw data for early blight resistance phenotype (*EB_2019_raw_pheno*) were used for a multivariate correlation test with other five traits and QTL analysis. Subsequent interpretation associated with accuracy of QTL analysis relying on raw 2019 early blight damage phenotype data will be further discussed in the Discussion section.

The distribution patterns of three Verticillium wilt damage BLUP datasets were almost normal when visually evaluated (**Supplementary Figure 3**). Two BLUPs of vine size (*VS_clo and VS_clo_2019*) were adjacent to a normal distribution, but *VS_clo_2020* were flat across the whole X-axis except one section from -0.5 to -0.75 cM, which had a significantly higher peak. Three BLUPS of vine maturity were also close to a normal distribution but departed slightly from the normal. Dataset *EB_2019_raw_pheno* was relatively skewed toward resistance (**Supplementary Figure 3**).

### Multivariate correlation tests for 16 BLUP datasets

Correlation tests were carried out for various combinations of BLUP data. Correlation coefficients can be found in **Supplementary Table 6**. Correlation coefficients for *LB*_clo, pooled late blight foliage damage phenotypic data across each year were approximately 0.9, but when correlation coefficients for *LB*_clo_2019 and *LB*_clo_2020 were compared, the result was a much lower correlation (0.6). LB-AUDPC BLUP datasets showed similar patterns (**Supplementary Table 6**). High correlation coefficients, near 1, were observed across the three BLUP datasets for VW, VM, and VS. These results exhibited consistency within each trait across the two years.

Interesting associations were observed from the correlation tests between different traits. Since high correlation coefficients were observed between BLUPs of pooled phenotypic data (e.g., XX_clo) and different year BLUP data (e.g., XX_clo_2019 or 2020) within the same trait, we compared pooled phenotypic data across two years (e.g., *LB_clo, LB-AUDPC_clo, VW_clo, VM_clo*, and *VS_clo*) to find potential links between those traits. Because of almost negligible segregation in 2020 early blight data, the *EB_2019_raw_pheno* dataset was used for the correlation test. As expected, the correlation between *LB_clo* and *LB-AUDPC_clo* was 0.96 because the *LB-AUDPC_clo* BLUP was based on LB raw phenotype data. When *LB* and *LB-AUDPC* (late blight resistance associated BLUP data) were compared with resistances against *EB* and *VW*, their correlation coefficients ranged from 42% to 59% (**Supplementary Table 6**), suggesting potential connections between those resistances or genes. Interestingly, the relationship was negative when VM and VS BLUPs were compared to those pathogen resistance BLUP data (*LB, LB-AUDPC, EB*, and *VW*) (**Supplementary Table 6**). This result insinuated that those clones with late maturity tended to display younger and more healthy vine status, making them more resistant to Verticillium wilt and early and late blight during the period of those pest infestations.

### QTL for LB, LB-AUDPC, EB, VW, VM, and VS

**Figure 2:**
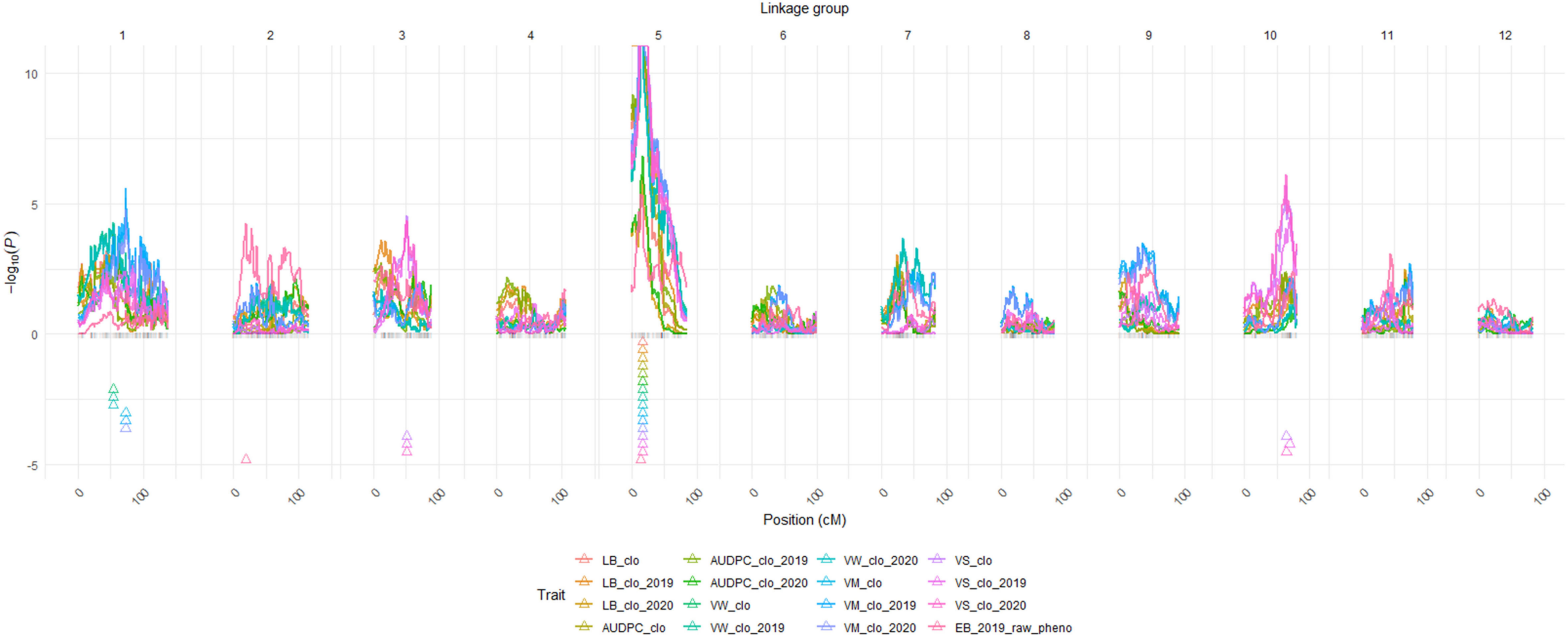
QTL maps for the six traits. BLUP data abbreviations: *LB*, Late Blight Foliage Damage; *LBAUDPC*, Late Blight Area Under the Disease Progress Curve; *EB*, Early Blight resistance; *VW*, Verticillium Wilt resistance; *VM*, Vine Maturity; *VS*, Vine Size; *clo*, a genetic effect of clones; *2019*, 2019; *2020*, 2020 year effects; Triangles indicate the locations of significant QTL peaks. Y axis represents LOP score, which equals – log10 (p-value). *Panel size limit of the QTLpoly prevented QTL having LOP scores over 11 from being completely visualized on chromosome 5 in this figure.

**Figure 3:**
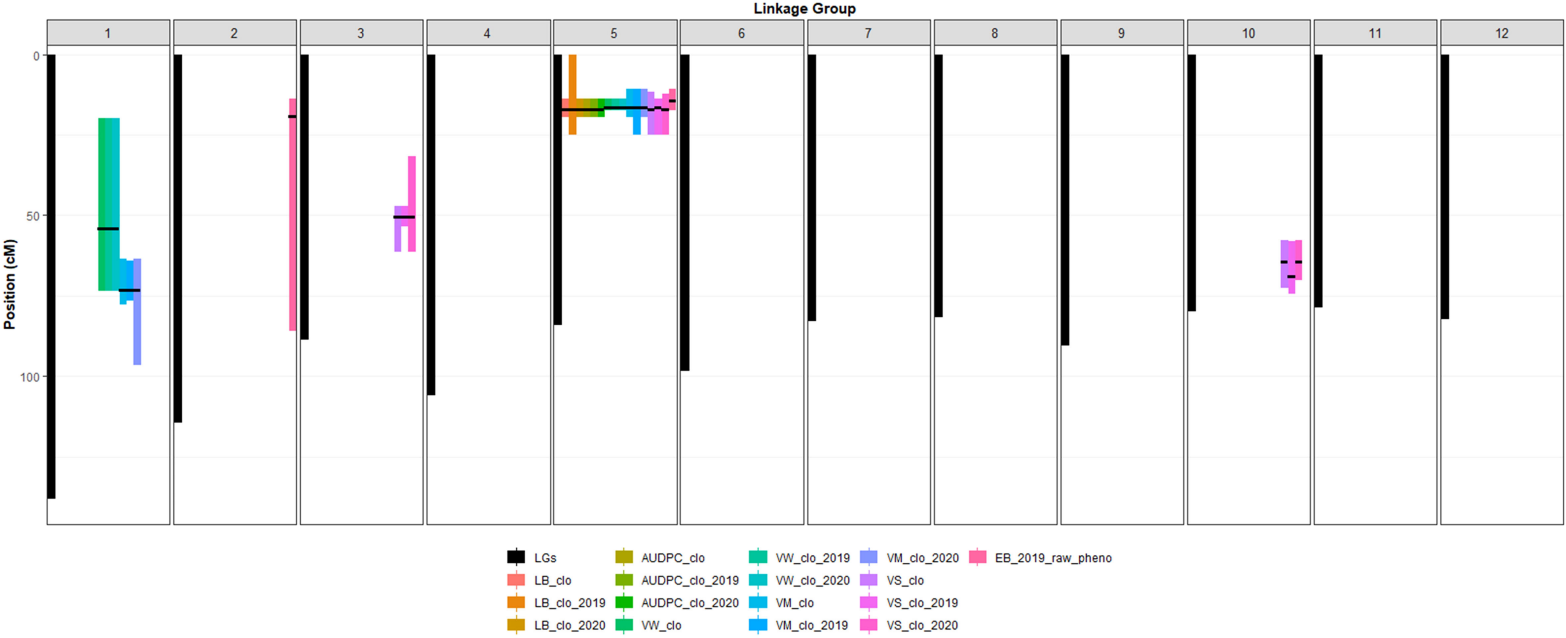
Location of significant QTL peaks and their support intervals. The X axis in **Figure 3** represents 12 different potato chromosomes. Black bars indicate length of each chromosome. Color bars indicate length of each support interval. Support intervals are labeled as described in **Figure 2**. Black thin horizontal lines on each support interval indicate the locations of the mapped significant QTL peaks.

The BLUP datasets (or raw phenotype data) of the six traits (**Supplementary Table 5**) and the 12 linkage groups (**Supplementary Figure 2**) were loaded on QTLpoly software to initiate QTL analyses. Overall, QTL mapping procedures were automatically conducted by the *remim* function equipped in QTLpoly, producing 12 complete QTL maps with LOP score, location including both chromosome number and exact position in centimorgan, support intervals, the heritability of the significant QTL (*h2QTL*), and proximate SNP markers to the mapped QTL (**Figures 2**, **3**; **Table 3**). Thanks to the *qtl_effects* argument of QTLpoly, meticulous investigation for each allele effect of the significant QTL positions was feasible (**Supplementary Figure 4**). A total of three, three, six, six, nine, and two QTL were detected for *LB, LB-AUDPC, VW, VM*, *VS*, and *EB*, respectively (**Table 3**).

**Table 3:** Summary table of QTL for *LB, LB-AUDPC, EB, VW, VM*, and *VS*.

QTL titles	BLUP datasets ^a^	Chr ^b^	LOP Score	Heritability of mapped QTL (*h^2^_QTL_*)	QTL Position (Support Interval) [Unit: cM] ^c^	Closest marker ^d^
*LB_clo_ch5*	*LB_clo*	5	>15.65 ^e^	0.56	**17.09** (13.90 - 19.07)	PotVar0077880 ^1)^
*LB_clo_2019_ch5*	*LB_clo_2019*	5	>15.65 ^e^	0.71	**17.09** (0 - 24.79)	PotVar0077880 ^1)^
*LB_clo_2020_ch5*	*LB_clo_2020*	5	6.01	0.29	**17.09** (13.90 - 19.07)	PotVar0077880 ^1)^
*LB-AUDPC_clo_ch5*	*LB-AUDPC_clo*	5	>15.65 ^e^	0.61	**17.09** (13.90 - 19.07)	PotVar0077880 ^1)^
*LB-AUDPC_clo_2019_ch5*	*LB-AUDPC_clo_2019*	5	>15.65 ^e^	0.65	**17.09** (13.90 - 19.07)	PotVar0077880 ^1)^
*LB-AUDPC_clo_2020_ch5*	*LB-AUDPC_clo_2020*	5	6.81	0.30	**17.09** (13.90 - 19.07)	PotVar0077880 ^1)^
*VW_clo_ch1*	*VW_clo*	1	4.20	0.07	**54.07** (19.69 - 73.16)	c2_37574 ^2)^
*VW_clo_ch5*	*VW_clo*	5	>15.65 ^e^	0.57	**16.54** (13.90 - 17.09)	PotVar0026113
*VW_clo_2019_ch1*	*VW_clo_2019*	1	4.28	0.07	**54.07** (19.69 - 73.16)	c2_37574 ^2)^
*VW_clo_2019_ch5*	*VW_clo_2019*	5	>15.65 ^e^	0.57	**16.54** (13.90 - 17.09)	PotVar0026113
*VW_clo_2020_ch1*	*VW_clo_2020*	1	4.09	0.07	**54.07** (19.69 - 73.16)	c2_37574 ^2)^
*VW_clo_2020_ch5*	*VW_clo_2020*	5	>15.65 ^e^	0.57	**16.54** (13.90 - 17.09)	PotVar0026113
*VM_clo_ch1*	*VM_clo*	1	5.20	0.08	**73.16** (63.40 - 77.36)	c1_6288
*VM_clo_ch5*	*VM_clo*	5	>15.65 ^e^	0.68	**16.54** (10.61 - 19.07)	PotVar0026113
*VM_clo_2019_ch1*	*VM_clo_2019*	1	5.60	0.08	**73.16** (64.00 - 76.30)	c1_6288
*VM_clo_2019_ch5*	*VM_clo_2019*	5	>15.65 ^e^	0.67	**16.54** (10.61 - 24.79)	PotVar0026113
*VM_clo_2020_ch1*	*VM_clo_2020*	1	4.75	0.07	**73.16** (63.40 - 96.16)	c1_6288
*VM_clo_2020_ch5*	*VM_clo_2020*	5	>15.65 ^e^	0.68	**16.54** (10.61 - 19.07)	PotVar0026113
*VS_clo_ch3*	*VS_clo*	3	4.54	0.05	**50.37** (47.00 - 61.17)	PotVar0120301
*VS_clo_ch5*	*VS_clo*	5	>15.65 ^e^	0.62	**17.09** (11.65 - 24.79)	PotVar0077880 ^1)^
*VS_clo_ch10*	*VS_clo*	10	5.68	0.10	**64.49** (57.64 - 72.42)	c2_48127
*VS_clo_2019_ch3*	*VS_clo_2019*	3	4.32	0.05	**50.37** (47.0 - 53.28)	PotVar0120301
*VS_clo_2019_ch5*	*VS_clo_2019*	5	>15.65 ^e^	0.62	**16.54** (13.9 - 24.79)	PotVar0026113
*VS_clo_2019_ch10*	*VS_clo_2019*	10	4.46	0.08	**69.09** (58.0 - 74.04)	c2_22594
*VS_clo_2020_ch3*	*VS_clo_2020*	3	4.33	0.05	**50.37** (31.52 - 61.17)	PotVar0120301
*VS_clo_2020_ch5*	*VS_clo_2020*	5	>15.65 ^e^	0.61	**17.09** (12.30 - 24.79)	PotVar0077880 ^1)^
*VS_clo_2020_ch10*	*VS_clo_2020*	10	6.11	0.12	**64.49** (57.64 - 70.00)	c2_48127
*EB_2019_pheno_ch2*	*EB_2019_raw_pheno*	2	4.24	0.10	**19.30** (13.71 - 85.55)	c2_37254 ^3)^
*EB_2019_pheno_ch5*	*EB_2019_raw_pheno*	5	5.37	0.38	**14.33** (10.61 - 17.09)	c2_11961 ^4)^

a BLUP data abbreviations: LB, Late Blight Foliage Damage; LB-AUDPC, Late Blight Area Under the Disease Progress Curve; EB, Early Blight resistance; VW, Verticillium Wilt resistance; VM, Vine Maturity; VS, Vine Size; clo, a genetic effect of clones; 2019, 2019 year effect; 2020, 2020 year effect. The details of these BLUP datasets are described in **Supplementary Table 5**.

Unlike the other traits, the 2019 raw early blight damage data were subjected to QTL analysis of EB.

b Chromosome numbers.

c Bold figures indicate locations of mapped QTL peaks; numbers in parentheses indicate ranges of support intervals.

d The most adjacent SNPs to each QTL peak were presented in this column; “solcap_snp_” was omitted at the beginning of all the SNP marker names beginning with either “c1” or “c2”.

e Maximum LOP score reported by QTLpoly software is 15.65.

^1)-4)^ If more than one SNP marker is located at the same position, the rest of the SNPs are written below.

^1)^ PotVar0078045, PotVar0078222, PotVar0078411, and PotVar0078439.

^2)^ solcap_snp_c2_37571.

^3)^ solcap_snp_c1_11120.

^4)^ solcap_snp_c2_11923, PotVar0025440, PotVar0025527, PotVar0025554, solcap_snp_c2_11896, and PotVar0025817.

#### QTL associated with late blight resistance

Six significant QTL for *LB* plus *LB-AUDPC* were found at the same position, 17.09 cM on chromosome 5, across two years (**Figure 2**; **Table 3**). SNP marker PotVar0077880 was closest to all six QTL. Support intervals of QTL associated with both *LB* and *LB-AUDPC* were from 13.90 to 19.07 cM, except that range for *LB_clo_2019_ch5* was wider - from 0 to 24.79 cM (**Figure 2**; **Table 3**). Very high LOP scores were found for QTL for *LB* and *LB-AUDPC*, with *LB_clo_ch5, LB_clo_2019_ch5, LB-AUDPC_clo_ch5*, and *LB-AUDPC_clo_2019_ch5* QTL exhibiting the maximum LOP value of 15.65 that QTLpoly software can provide (**Figure 2**; **Table 3**). *LB_clo_2020_ch5* and *LB-AUDPC_clo_2020_ch5* QTL also had 6.01 and 6.81 LOP scores, respectively, being selected as significant QTL. Their *h^2^_QTL_* of *LB_clo_ch5, LB_clo_2019_ch5, LB-AUDPC_clo_ch5*, and *LB-AUDPC_clo_2019_ch5* QTL were also remarkably larger, ranging from 56% up to 71%. The *h^2^_QTL_* of *LB_clo_2020_ch5* and *LB-AUDPC_clo_2020_ch5* QTL were 30% or similar (**Figure 2**; **Table 3**).

#### QTL associated with VW, VM, VS, and EB

A major QTL for VW, VM, VS, and EB was observed at 16.54 cM on chromosome 5 with extremely high LOP and QTL heritabilities (*h^2^_QTL_*) (e.g., *VW_clo_ch5, VW_clo_2019_ch5, VW_clo_2020_ch5, VM_clo_ch5, VM_clo_2019_ch5, VM_clo_2020_ch5, and VS_clo_2019_ch5*). Other QTL on chromosome 5 were observed near 16.54 cM on chromosome 5 (e.g., *EB_2019_pheno_ch5* at 14.33 cM; *VS_clo_ch5* and *VS_clo_2020_ch5* at 17.09 cM) (**Table 3**). Markers adjacent to those QTL were PotVar0026113, solcap_snp_c2_11961, solcap_snp_c2_11923, PotVar0025440, PotVar0025527, PotVar0025554, solcap_snp_c2_11896, PotVar0025817, PotVar0077880, PotVar0078045, PotVar0078222, PotVar0078411, and PotVar0078439. The *h^2^_QTL_* of all the QTL near the 16.54 cM were high ranging from 38% to 68%, verifying all those QTL are major (**Table 3**). On chromosome 1, minor QTL for VW and VM were observed at 54.07 and 73.16 cM, respectively, across the two years (**Figure 2**; **Table 3**). On chromosomes 3 and 10, the minor QTL (or close to minor) for VS were also consistently detected, across the two years. On chromosomes 2 *EB_2019_pheno_ch2* QTL was observed at 19.30 cM. All the details of each QTL were organized in **Table 3**.

### Allelic effects of mapped QTL for Late Blight

This allelic effect analysis could indicate which parent (or allele) predominantly contributes each of the six traits studied in this research. Investigation of allelic effects indicated that all QTL on chromosome 5 exerted the strongest impacts on significant QTL regardless of trait. Details of allelic effects are further discussed below.

#### Allelic effect analyses of LB and LB-AUDPC

When the allele effects of the QTL for LB and LB-AUDPC were inspected together, a total of 24 allele effects (four homologs × two parents × three QTL) were commonly detected for each trait, respectively, consisting of 10 positive (an allele effect worsening late blight damage) and 14 negative alleles (another allele effect alleviating late blight damage) (**Supplementary Figure 4**; **Supplementary Table 7**). The comparison of the absolute values of the two parents’ allele effect showed that Palisade Russet and ND028673B-2Russ provided 3.56 (42.22%) and 4.87 (57.78%) contributions to *LB* respectively. Similarly, Palisade Russet and ND028673B-2Russ had 1047.06 (45.13%) and 1273.29 (54.87%) contributions to LB-AUDPC, respectively, which was surprising in that a greater contribution to late blight resistance was anticipated from Palisade Russet, which is late blight resistant. Therefore, unlike the normal pathogen resistance gene (e.g., potato virus Y resistance genes), the presence (or absence) of the susceptible allele(s) of the *LB* resistance-associated gene also seems to be a key factor in contributing to the phenotypic response to late blight infection observed in the segregating population. More details associated with the allele effect values of the two parents’ contribution were provided in **Supplementary Figure 4**; **Supplementary Table 7**.

#### Allelic effect analyses of VW, VM, VS, and EB

The total absolute values of the *VW* allele effects of Palisade Russet and ND028673B-2Russ were 3.40 (43.02%) and 4.51 (56.98%). ND028673B-2Russ showed a higher impact than Palisade Russet across the six significant QTL associated with *VW* resistance (**Supplementary Table 7**). Significant QTL for VW were detected on chromosomes 1 and 5, and interestingly, the QTL on chromosome 5 (*VW_clo_ch5, VW_clo_2019_ch5, and VW_clo_2020_ch5*) persistently showed higher impact than those on chromosome 1 (**Supplementary Table 7**).

The total absolute values of *VM* allelic effects of Palisade Russet and ND028673B-2Russ were 4.56 (43.39%) and 5.95 (56.61%). Palisade Russet showed higher effects at *VM_clo_ch1* QTL (59.73%), *VM_clo_2019_ch1* QTL (58.91%), and *VM_clo_2020_ch1* QTL (60.61%). ND028673B-2Russ revealed stronger efficacy at *VM_clo_ch5* QTL (61.13%), *VM_clo_2019_ch5* QTL (60.94%), and *VM_clo_2020_ch5* QTL (61.33%). As observed in *VW* allelic effects above, allelic effects of QTL on chromosome 5 (*VM_clo_ch5, VM_clo_2019_ch5*, and *VM_clo_2020_ch5*) were consistently higher than those on chromosome 1 (**Supplementary Table 7**).

The total absolute values of the *VS* allele effects of Palisade Russet and ND028673B-2Russ were 4.40 (51.89%) and 4.08 (48.11%). At the QTL: *VS_clo_ch5, VS_clo_2019_ch5*, and *VS_clo_2020_ch5*, ND028673B-2Russ showed higher effects than Palisade Russet. The rest of the significant QTL showed a higher allele effect in Palisade Russet than ND028673B-2Russ. Like the allele effect analysis results of the previous traits, the allele effects of the QTL on chromosome 5 (*VS_clo_ch5, VS_clo_2019_ch5*, and *VS_clo_2020_ch5*) were consistently higher than those on chromosomes 3 and 10 (**Supplementary Table 7**).

Finally, the total absolute values of *EB* allelic effects of Palisade Russet and ND028673B-2Russ were 1.21 (45.82%) and 1.43 (54.18%). At *EB_2019_pheno_ch2 QTL*, the allelic effect of Palisade Russet was slightly higher than ND028673B-2Russ. At *EB_2019_pheno_ch5* QTL, ND028673B-2Russ showed a higher impact than Palisade Russet. As before, allelic effects of QTL on chromosome 5 exerted greater impact than those on chromosome 2 (**Supplementary Table 7**).

### Haplotype dissimilarities between late blight resistant and susceptible panels

Seventeen resistant and sixteen susceptible clones were selected, consistently earning their top 20% ranking for late blight resistance and susceptibility, respectively across years. Examples of their haplotype images were visualized, and a summary of the haplotype comparison tests was included in **Supplementary Figure 5**. Since all the QTL for late blight resistance (*LB* & *LB-AUDPC*) were consistently located at 17.09 cM on chromosome 5 (**Figure 2**; **Table 3**), we mainly focused on the distinction at this target position (17.09 cM) on chromosome 5 while scrutinizing the haplotypes between resistant and susceptible groups. Before discussing the haplotype comparison results, it was confirmed that the homologs b, e, and h at all the six *LB* & *LB-AUDPC* QTL (located at 17.09 cM on chromosome 5) had positive (susceptible) effects, but homologs a, c, d, f, and g had negative (resistant) effects (**Supplementary Figure 5**). Interestingly, while observing the resistant panel, we found that the majority of the individuals did not have the positive effect alleles on homologs b, e, and h but had the negative effect alleles on homologs a, c, d, f, and g. On the other hand, the susceptible clones did not tend to have the negative allele effects on homolog d but tended to have the positive effect alleles on homologs b and h (**Supplementary Figure 5**).

## Discussion

### Investigation for correspondence between years within a trait and between traits

When the BLUPs of each trait with different years were compared, it turned out that most of the analyzed traits showed high consistency across the two years with a few outliers, indirectly proving the reliabilities of phenotype measurement activities and low variation between the two years. Exceptionally, the comparison between *LB_clo_2019* and *LB_clo_2020*, as well as another comparison between *LB-AUDPC_clo_2019* and *LB-AUDPC_clo_2020*, showed a relatively lower correlation coefficient (about 0.60) compared to other correlation test results. However, it should be noted that the late blight damage evaluations were conducted in an outdoor potato field, with many variation factors. Besides, the infection rate and propagation of *P. infestans* were known to be remarkably affected by environmental conditions such as humidity and temperature (Skelsey et al., 2010). Despite the potential presence of multiple variation factors, those correlation tests maintained at least reasonable or extremely high coefficients (from 0.57 to 0.93; **Supplementary Table 6**), reinforcing low variation between two years.

The correlation tests between *LB_clo, LB-AUDPC_clo, EB_clo*, and *VW_clo* disclosed all the correlation coefficients were positive, ranging from 43% to 59% (**Supplementary Table 6**). It is postulated that some of those pathogen resistances against *LB*, *LB-AUDPC, EB*, and *VW* might result from a shared resistance mechanism with the reported QTL analysis results also supporting this hypothesis.

All correlation testing between vine size and pooled pathogen resistance BLUP datasets resulted in negative coefficients, varying from -0.49 to -0.64. These negative coefficients may indicate that the three diseases impact vine growth of susceptible potato plants, reflected in the negative coefficients observed. Negative correlations were also detected between *VM_clo* and pooled pathogen resistance BLUP datasets, which ranged from -0.49 to -0.64; maturity was impacted by plant response to infection. Previous research identified similar relationships. Visker et al. (2003) and Bradshaw et al. (2004) studied late blight resistance and foliage maturity, based on diploid and tetraploid mapping populations, respectively. Interestingly, they localized a significant QTL for late blight and maturity on chromosome 5, as observed in this study. Visker et al. (2003) found that early maturing clones tend not to be resistant to late blight. Multiple Verticillium wilt-associated research reported that the photosynthetic decline caused by *V. dahliae* infection might occur through activation of the StCDF1 maturity (Tai et al., 2018) and tuberization pathway. Verticillium-induced early vine maturity leads to smaller vines, reduced resources transferred to tubers and significantly reduced yields (Rowe and Powelson, 2002; Simko et al., 2004; Jansky and Miller, 2010; Simko and Haynes, 2016; Tai et al., 2018). Early blight resistance of potatoes was also known to be associated with potato maturity type and late-maturing cultivars were somewhat advantageous to control *EB* damage compared to early maturing cultivars (Boiteux et al., 1995; Rodriguez et al., 2006; Duarte et al., 2014). Our findings support these observations as well.

### Automated linkage and QTL mapping

Even though direct QTL analysis for tetraploid potatoes is possible using TetraploidSNPMap (TPMSNP), QTL analyses of tetraploid mapping populations has not been as commonplace as with the use of diploid potatoes. This is due to the marker phasing process being fully automated in most diploid linkage mapping software, but not being entirely automated in TPMSNP using tetraploid mapping populations, where manual input is commonly required.

Newly released R-package MAPpoly (Mollinari and Garcia, 2019; R Development Core Team, 2020) has improved manual marker phasing, resulting in fully automated linkage mapping in tetraploid potato (Park et al., 2021). This has considerably shortened elapsed time for linkage mapping and minimized complexities previously encountered in manual marker phasing. Combining MAPpoly and QTLpoly automates tetraploid QTL analysis and is used in potato breeding programs (Park et al., 2021). Higher precision in development of 12 linkage groups can be achieved with MAPpoly using potato reference genome PGSC Version 4.03 (Sharma et al., 2013; Hirsch et al., 2014; Spud Database, 2020) while assembling groups. Therefore, the 4040 selected markers in this study were evenly assigned across the 12 linkage groups without a wide gap between two SNPs (**Figure 1**). Almost perfect genome coverage rates were also obtained from all the genetic maps (**Table 1**). These successful outcomes reflect the reliability and benefits of the fully automated QTL analysis pipeline with the shorter elapsed time and higher accuracy than previously encountered with autotetraploid QTL analyses using TPMSNP, thereby expediting the development and use of MAS development in potato breeding programs.

Meanwhile, the *LB, LB-AUDPC*, and *EB* BLUP datasets deviated from a normal distribution; thus, ancillary QTL analyses with transformed data relatively closer to normal were performed to appraise whether the non-normal distributions significantly impacted QTL results or not. For the data transformation, the Ordered Quantile (ORQ) normalization transformation method was utilized (Peterson and Cavanaugh, 2020). The comparison of transformed and non-transformed BLUP datasets showed no significant difference in the major QTL chromosomal positions (data not shown). Therefore, the non-transformed BLUP datasets and their results were used in this study.

### QTL associated with late blight infection of foliage

One important QTL for *LB* and *LB-AUDPC* was observed on chromosome 5 at 17.09 cM, with high LOP and h^2^_QTL_ scores across two years. Mapped *LB* and *LB-AUDPC* QTL had the same support interval area, from 13.90 to 19.07 cM, except for *LB*_clo_2019_ch5 QTL, with a wider support interval (**Figure 3**; **Table 3**). These results suggested the presence of a single major locus impacting late blight resistance on this chromosome. Five SNPs (PotVar0077880, PotVar0078045, PotVar0078222, PotVar0078411, and PotVar0078439) were observed at 17.09 cM (**Table 3**; **Supplementary Figure 2**). Allelic effect analyses for *LB_clo_ch5* QTL showed positive (susceptible) effects on homologs b, e, and h and negative (resistance) allelic effects on homologs a, c, d, f, and g (**Supplementary Figure 4**). Haplotype comparison testing indicated that individuals belonging to the resistant panel tended toward greater negative effect alleles (on homologs a, c, d, f, and g) than positive effect alleles (on homologs b, e, and h), as expected (**Supplementary Figure 5**). On the other hand, the susceptible clones were inclined to have both negative and positive allele effects evenly represented (**Supplementary Figure 5**). Interestingly, 14 of 16 susceptible clones had a positive effect homolog b, reinforcing the importance of homolog b in increasing susceptibility to late blight (**Supplementary Figure 5**). However, given late blight damage phenotype data and allelic effect analysis results, mutual interaction (i.e., dominant or recessive) between positive and negative alleles could not be clearly explained. After developing a molecular marker to distinguish opposite effect alleles at the QTL position, PCR tests and accompanied bioassays, including late blight-resistant and -susceptible panels, are needed to elucidate their interactions.

Chromosome 5 has frequently come up as a hotspot when it comes to late blight resistance QTL. For instance, van Eck and Jacobsen (1996), Leonards-Schippers et al. (1992; 1994); Collins et al. (1999), and Visker et al. (2003) used bi-parental diploid mapping populations and identified foliage and tuber blight resistance QTL, or QTL associated with the R1 gene, on chromosome 5, at the zones between GP179 and GP21 RFLP markers or in close proximity to each marker. Śliwka et al. (2007) also localized QTL for resistance to *P. infestans* at CP113 (allele-specific amplification: ASA) and BA47f2t7 (cleaved amplified polymorphic sequence: CAPS) markers on chromosome 5, based on tuber observation data of a bi-parental diploid mapping population (Niewöhner et al., 1995). Lindqvist-Kreuze et al. (2021) found an environment-specific QTL effective to late blight on chromosome 5 while performing a genome-wide association study with genotyping by sequencing (GBS) markers and a trait observation network (TON) population composed of 380 genotypes, which represented seven International Potato Center (CIP) breeding populations and cultivars that came from various origins. Similar to observations in diploid potato populations, Enciso-Rodriguez et al. (2018) conducted a genomic selection study with a bi-parental tetraploid population, and they reported several SNPs on chromosome 5 were associated with late blight resistance. Our findings confirm previous reports of the importance of chromosome 5 with respect to late blight resistance, but in a tetraploid russet mapping population.

### QTL associated with response to Verticillium wilt infection

The genetic effect was much larger than the other effects in the variance component estimate (**Table 2**) for *VW*. Two QTL had been consistently observed on chromosomes 1 and 5 across the two years (**Table 3**). The *VW_clo_ch5, VW_clo_2019_ch5*, and *VW_clo_2020_ch5* QTL were detected on chromosome 5 at 54.07 cM, having >15.65 LOP score and 57% h^2^_QTL_. The support intervals of the three QTL identified on chromosome 5 were 13.90 to 17.09 cM (**Figure 3**; **Table 3**). The closest SNP marker was PotVar0026113. Interestingly, the PotVar0026113 SNP was placed at the middle of the PGSC0003DMG400030495 genome sequence coordinate, including the auxin efflux carrier gene (Potato Genome Sequencing Consortium, 2011; Sharma et al., 2013; Hirsch et al., 2014; Spud Database, 2020: http://solanaceae.plantbiology.msu.edu). Traditionally, auxin has been commonly known as a classical phytohormone, affecting leaf aging, plant and potato tuber development, etc., by interacting with cytokinins and other phytohormones (Obata-Sasamoto and Suzuki, 1979; Koda and Okazawa, 1983; Kolachevskaya et al., 2019). Auxin can either enhance or weaken plant resistance against biotrophic pathogens such as *Streptomyces scabies* and *Phytophthora infestans* (Tegg et al., 2008; Kazan and Lyons, 2014; Naseem et al., 2015; Kunkel and Harper, 2018; Natarajan et al., 2018). To the best of our knowledge following a review of the literature, no previous studies have associated auxin with Verticillium wilt control, however, the close proximity of the auxin efflux carrier gene to a SNP closely associated with *VW* resistance supports the possible influence of this hormone in a plant’s response to infection by *VW*. Multiple references have also reported *VW* resistance QTL on chromosome 5. Massa et al. (2018) tested 162 F1 progeny derived from a cross between Rio Grande Russet and Premier Russet in Idaho. They found *VW* resistant QTL at solcap_snp_c2_11605 on chromosome 5 with this SNP being 2.29 cM away from SNP PotVar0026113 identified in this study as being the most closely associated with a QTL for *VW* on chromosome 5 in this study (Hirsch et al., 2014; Spud Database, 2020: http://solanaceae.plantbiology.msu.edu). Tai et al. (2018) localized QTL for Verticillium wilt resistance on chromosomes 5 (VW_ch5_Tai QTL) and 9 (VW_ch9_Tai QTL) and described the epistatic relationship between the two QTL, using a diploid mapping population having *S. phureja* and *S. stenotomum* background. They explained the VW_ch5_Tai QTL had a major effect and the VW_ch5_Tai QTL’s support interval included the StCDF1 gene, which controls maturity and tuberization earliness. Another QTL, VW_ch9_Tai QTL, co-localized with the known Verticillium wilt resistance gene, Ve2 (Tai et al., 2018). The epistasis analysis and gene ontology analyses conducted by Tai et al. (2018) elucidated that StCDF1 functioned downstream of Ve2. Furthermore, Ve2 influenced fungal defense and reduced early dying in Verticillium wilt invasion by involving a genetic pathway controlling tuber organogenesis timing. Cycling DOF factors (CDFs) are components of the transcriptional regulatory networks involved in controlling abiotic stress responses (Renau-Morata et al., 2020). The DOF represents DNA-binding with one finger (Salaria et al., 2020). In potatoes, *Solanum tuberosum* CDFs (StCDFs) are a cluster of transcriptional repressors affecting earliness in potatoes (Salaria et al., 2020). Interestingly, previous studies provided evidence that some DOF factors might play important roles in responses to plant hormones, including auxins (De Paolis et al., 1996; Kisu et al., 1998). Since the distance between the StCDF1 and auxin efflux carrier genes was less than 0.3 cM, it is not conclusive at to which of the two may be associated with the *VW* QTL identified in this study (Hirsch et al., 2014; Spud Database, 2020: http://solanaceae.plantbiology.msu.edu). Further analyses of SNPs near the StCDF1 and the auxin efflux carrier genes on the chromosome linkage map 5 developed in this study are warranted for future MAS for Verticillium wilt resistance.

On chromosome 1, another *VW* resistance QTL was consistently observed at 54.07 cM across the three BLUP datasets: *VW_clo, VW_clo_2019*, and *VW_clo_2020*. The support intervals of the three QTL occupied a relatively wider range from 19.69 to 73.16 cM (**Figure 3**; **Table 3**). These QTL seems to be relatively minor compared to the three *VW*_resistancce QTL on chromosome 5 because their approximate average LOP score was 4.20 as well as their h^2^_QTL_ were commonly 7% (**Table 3**). QTLpoly reported the two SNPs, solcap_snp_c2_37574 and solcap_snp_ c2_37571, as the linked markers to the QTL position. Both SNPs were located in the middle of PGSC0003DMG402006333 related to a protein kinase family protein (Hirsch et al., 2014; Spud Database, 2020: http://solanaceae.plantbiology.msu.edu). Li et al. (2018) discussed the cloned cotton cyclin-dependent kinase E (GhCDKE) gene, which was a subunit of the cotton (*Gossypium hirsutum*) Mediator complex and regulates disease resistance. Using *Agrobacterium tumefaciens*, they developed the transgenic *Arabidopsis* plants having overexpressed GhCDKE, and then inoculated the transgenic plants with *V. dahliae*. Interestingly, overexpression of GhCDKE enhanced resistance to *V. dahliae* (Li et al., 2018). Zhang et al. (2013) also observed that the *Arabidopsis*, which had overexpressed a serine/threonine protein kinase obtained from cotton (GbSTK), showed improved resistance to *V. dahliae*. Returning to the potato genetic study, Kumar et al. (2017) found a significant QTL on chromosome 1 while performing a QTL analysis for *VW* resistance with a diploid potato mapping population. Overall, all the experiment results of both current and previous studies commonly pointed out the interconnection between *VW* resistance mechanism and protein kinase family protein and reported the identification of the *VW* resistance QTL on chromosome 1, thereby warranting further examination of this region in the development of MAS for *VW*.

### QTL for vine maturity

QTL analyses for the *VM_clo, VM_clo_2019*, and *VM_clo_2020* BLUP datasets consistently resulted in two significant QTL on chromosomes 1 and 5, respectively. Interestingly, the three QTL, *VM_clo_ch5, VM_clo_2019_ch5*, and *VM_clo_2020_ch5* had similarities with the previously discussed *VW* QTL. For instance, they were commonly observed at 16.54 cM on chromosome 5 where the three *VW* resistance QTL were also found, with PotVar0026113 identified as the closest maker. Their LOP scores also reached the software’s maximum LOP limit (>15.65) (**Figure 2**; **Table 3**). The zone between 10.61 and 19.07 cM was commonly shared by the support intervals of all the three *VM* QTL (**Figure 3**; **Table 3**). Śliwka et al. (2007) mapped a QTL for vegetation period at BA47f2t7 CAPS marker on chromosome 5 after analyzing a diploid potato mapping population. Collins et al. (1999) found a major QTL for maturity on chromosome 5 between GP21 and GP179 RFLP markers with another diploid mapping population. This QTL included the most considerable effect showing between 56.4 and 70.6% of the phenotypic variance explained across the three-year data. Bradshaw et al. (2004) performed a QTL analysis with AFPL and SSR markers based on 277 clones derived from the cross between two tetraploid clones: 12601ab1 and the cultivar Stirling. They successfully mapped a QTL for early maturity at the STM3179 SSR marker on chromosome 5, which explained 41.5% of the variance for the trait. More recently, Hackett et al. (2014) prepared 190 F1 offspring developed from the same parents (Stirling & 12601ab1) and genotyped them with a high-throughput genotyping tool, Infinium 8303 potato SNP array (Felcher et al., 2012). The ability to further saturate chromosome regions with additional SNP makers, allowed the development of twelve high-density linkage groups, with a major QTL (about 55% contribution) for plant maturity localized in closest proximity to solcap_snp_c2_47609 on chromosome 5. Massa et al. (2018) also found a QTL for maturity at solcap_snp_c2_11605 on chromosome 5 across Idaho and North Carolina for two years.

The StCDF1 gene on chromosome 5, which was mentioned earlier in the *VW* resistance QTL section, has been frequently identified and studied by previous researchers as an important gene in the potato life cycle (e.g., earliness, maturity, flowering, tuberization, etc.) (Navarro et al., 2011; González-Schain et al., 2012; Kloosterman et al., 2013; Salaria et al., 2020). In a genome-wide association study (GWAS) with a panel composed of 277 tetraploid clones, Klaassen et al. (2019) confirmed that one of the QTL they found was on chromosome 5 and was associated with alleles of StCDF1. Since the physical map distance between the SNP, PotVar0079081, linked to the StCDF1 gene (Willemsen, 2018; Klaassen et al., 2019) and the SNP, PotVar0026113, linked to the three VM QTL identified on chromosome 5 in this study (**Table 3**) was only 0.24 cM, we hypothesized that those QTL seemed to reflect the effect of the StCDF1, with further investigation warranted.

On chromosome 1, another QTL for *VM* was consistently observed at 73.16 cM across the three *VM* BLUP datasets (**Figure 3**; **Table 3**). Compared to the significant *VM* QTL on chromosome 5, these QTL were more minor with LOP scores of 5.60 or lower, and their h^2^_QTL_ were 7 to 8% (**Table 3**). The adjacent SNP to this QTL position was solcap_snp_c1_6288, and the genotypes of the two parents at this marker were commonly AABB. When the allele effect at 73.16 cM on chromosome 1 was scrutinized, the B alleles of the SNP on homolog d and h seemed to be linked to the most powerful negative impact (**Supplementary Figure 4**). Collins et al. (1999) found minor QTL for early maturity on chromosome 1 near an SSR marker, STM1029. Since the STM1029 marker (chr01: 45754400.45761800) was 27.5 cM away from solcap_snp_c1_6288 (chr01: 73262904), the maturity QTL on chromosome 1 found by Collins et al. (1999) appeared to differ from QTLs associated with *VM_clo_ch1, VM_clo_2019_ch1*, and *VM_clo_2020_ch1*. Even though those QTL were relatively minor and could not be cross-checked with references, it is still worthwhile to consider the information on the QTL for a breeding program. This is because the *VM* QTL on chromosome 5 could not be easily distinguished from another pathogen resistance QTL discussed above; thus, it is not easy to directly use them to control vine maturity exclusively. On the other hand, *VM_clo_ch1, VM_clo_2019_ch1*, and *VM_clo_2020_ch1* exclusively existed without overlapping with other QTL.

### QTL for vine size

QTL controlling vine size were observed on chromosome 3, 5, and 10 across the three BLUP datasets. The three most consequential QTL (*VS_clo_ch5, VS_clo_2019_ch5*, and *VS_clo_2020_ch5*) were commonly observed on chromosome 5, with >15.65 (maximum) LOP score and 62% h^2^_QTL_. Even though the location of *VS_clo_2019_ch5* differed from those of the other two QTL, they were only 0.55 cM, and their support intervals commonly shared the zone between 13.90 and 24.79 cM; thus, the three QTL were inferred to represent one gene (**Figures 2**, **3**; **Table 3**). The most proximate SNP of *VS_clo_2019_ch5* QTL was PotVar0026113. Those of *VS_clo_ch5* and *VS_clo_2020_ch5* were PotVar0077880, PotVar0078045, PotVar0078222, PotVar0078411, and PotVar0078439. It should be noted that *VS_clo_ch5* and *VS_clo_2020_ch5* were located at the same positions where the *LB* and *LB-AUDPC* QTL were previously located (**Figures 2**, **3**; **Table 3**). When Hackett et al. (2014) performed a QTL analysis with 190 progenies from the cross between Stirling and 12601ab1, they found significant QTL for canopy height at solcap_snp_c2_47609 with 30.1% of the variance in the height. This SNP is only 1.57 cM away from *VS_clo_ch5* and *VS_clo_2020_ch5* (PotVar0077880) and 1.72 cM away from *VS_clo_2019_ch5* (PotVar0026113), respectively. Our results and those of Hackett et al. (2014) appear to corroborate one another and indicate the presence of a gene in this region of chromosome 5 associated with vine size using differing tetraploid mapping populations.

The three QTL on chromosome 3 (*VS_clo_ch3, VS_clo_2019_ch3*, and *VS_clo_2020_ch3*) showed minor effects with a 4.4 LOP score and 5% h^2^_QTL_. The effects of the three QTL on chromosome 10 were also relatively minor, showing between 4.46 and 6.11 LOP scores and an average of 10% h^2^_QTL_. The one SNP, PotVar0120301 and another two SNPs, solcap_snp_c2_22594 and solcap_snp_c2_48127 were the most adjacent markers to the QTL on chromosome 3 and 10, respectively.

Most potato breeding-related research projects have focused on either potato disease resistance or other traits directly influencing economic gains (e.g., tuber quality, size, shape, processed food quality, etc.). Consequently, potato vine size has not been emphasized compared to other more economically attractive agronomic traits. However, the lengths and canopy patterns of the upper parts of the potato can increase the light absorption rate for photosynthesis, affecting yield, plant health, etc. For example, Khayatnezhad et al. (2011) confirmed a strong positive correlation between tuber yield and plant height. Furthermore, vine size can also impact potato management methods during the growing season and at vine kill prior to harvest. Therefore, it is worthwhile to delve into those *VS* QTL and PotVar0078045 SNP to select clones having appropriate canopy sizes and patterns adapted to local environmental conditions and farming techniques.

### QTL associated with foliar response to infection by early blight

Two different QTL for *EB* resistance were found on chromosomes 2 and 5 while analyzing *EB_2019_raw_pheno* dataset (**Table 3**). Among them, the QTL on chromosome 5 boasted its greatest influences with higher LOP scores and h^2^_QTL_. The seven SNPs, solcap_snp_c2_11961, solcap_snp_c2_11923, PotVar0025440, PotVar0025527, PotVar0025554, solcap_snp_c2_11896, and PotVar0025817 were linked to *EB_2019_pheno_ch5*. Odilbekov et al. (2020) performed a QTL analysis for early blight resistance with an F1 tetraploid potato population derived from a cross between the cultivars “Matilda” (susceptible) and “Magnum Bonum” (resistant). Interestingly, they not only found *EB* foliar resistant QTL on chromosome 5 but also reported the SNP, PotVar0026113, as the proximate marker to the *EB* resistant QTL (Odilbekov et al., 2020). The PotVar0026113 SNP was only 0.71 cM away from solcap_snp_c2_11961. Massa et al. (2018) localized EB resistance QTL at solcap_snp_c2_11605, which is approximately 1.58 cM away from solcap_snp_c2_11961 (Potato Genome Sequencing Consortium, 2011; Sharma et al., 2013). Therefore, the results of this study in combination with the findings of Odilbekov et al. (2020) and Massa et al. (2018) do provide evidence of the importance of this region of chromosome 5 with respect to the development of MAS for *EB* resistance. Additional investigation with advanced materials and technologies (e.g., chromosome walking or fine mapping with an increased size of the mapping population) for the area near those SNP markers is warranted for the development of MAS for *EB*.

Another *EB* QTL having relatively lower LOP and h^2^_QTL_ were detected on chromosomes 2. The *EB_2019_pheno_ch2* QTL was located at 19.30 cM. Compared to other QTL, the *EB_2019_pheno_ch2* QTL has extremely wider support interval (over 71 cM length), suggesting the potential existence of at least more than one minor *EB* resistance-associated QTL or more on this chromosome (**Figure 3**; **Table 3**). Odilbekov et al. (2020) reported QTL for tuber resistance against the *A. solani* (the causal agent of potato EB) on chromosome 2, but their results could not be compared with this current study because *EB* resistances of tubers were not evaluated.

Since the one-year raw phenotype dataset was only usable and was used for this *EB* QTL analysis, unlike the other QTL analyses, an additional verification process was executed to evaluate the reliability of the QTL results from the raw phenotype data. Both 2019- and 2020-year raw data (which did not segregate) were first converted to EB BLUP datasets, based on the mixed model (eq. 2), and then the BLUP datasets were loaded on the QTLpoly to run a new QTL analysis. As expected, the 2020 *EB* BLUP data produced no significant QTL, but the 2019 *EB* BLUP dataset showed two significant QTL which were located at the exact same positions and chromosomes of the *EB_2019_pheno_ch2* and *EB_2019_pheno_ch5* QTL, respectively (data not shown). Furthermore, the two QTL derived from the *EB* BLUP data had the same (or almost identical) LOP scores, QTL heritabilities (h^2^_QTL_), and support intervals compared to those of the *EB_2019_pheno_ch2* and *EB_2019_pheno_ch5* QTL (data not shown). Those same (or almost the same) results between the two different QTL analyses with raw *EB* damage phenotype and EB BLUP data, respectively, reflect that the direct use of the raw phenotype data did not significantly affect the QTL results. However, further QTL analysis with multiple-year data is necessary to scrutinize the consistency of the *EB_2019_pheno_ch2* and *EB_2019_pheno_ch5* QTL as well as an interaction between an environmental effect and the two QTL.

### QTL hotspot on chromosome 5

Interestingly, the region between 14.33 and 17.09 cM (A08241_14-17_hotspot, an abbreviation representing the zone between 14.33 and 17.09 cM) on chromosome 5 was repeatedly identified as having significant QTL for the three-potato pathogen resistances and two agronomic traits assessed in this study, even though their relationships were not obviously explained (**Table 3**). A literature search was conducted with references being found relating to the examined traits with QTL physical map locations being reported and previously discussed. The physical map location information (PGSC version 4.03) reported allowed us to objectively compare each reference’s QTL positions to those of ours, in that they do not change regardless of mapping populations. **Supplementary Table 8** has organized physical map locations of all the SNP linked to significant QTL detected in the literature and the current study, allowing an overall summarization of the previous discussion, with the term “A08241_QTL analyses” used to distinguish our QTL analyses from those of the other references. The comparative analyses in **Supplementary Table 8** show considerable similarities in physical map location were consistently observed across multiple studies and our own for the traits examined, even though different mapping populations and varied environmental conditions were used. When we focus on, for instance, the locations of all the QTL for late blight, which were localized by the A08241_QTL analysis and references, all of them considered in **Supplementary Table 8** were near one to one another with less than a 1.82 cM gap. Similar patterns were also observed in the results of the *VW, VM, VS*, and *EB* QTL analyses while comparing the references with our study (**Supplementary Table 8**), reinforcing the presence of the genes controlling *LB, EB, VW, VM*, and *VS* near the identified QTL in this study, and corroborating the importance of chromosome 5 for further development of MAS in cultivated potato. The A08241_14-17_hotspot identified on chromosome 5 (~2.76 cM length) is thought to represent either a gene having a pleiotropic effect on several apparently unrelated traits (Collins et al., 1999; Visker et al., 2003), or the existence of a family of genes each impacting singly a trait, but in close proximity to one another. Additional study is warranted to better delineate which scenario (or combination of the two) is most plausible.

## Conclusion

The QTL analyses for *LB, LB-AUDPC, EB, VW, VM*, and *VS* provided useful genetic information, which can be used for future MAS or potato breeding programs of the russet market class. On chromosome 5, the A08241_14-17_hotspot emerged as an essential genetic location for all the five traits evaluated in this study; thus, detailed research on this hotspot is expected to create greater added value in the future russet potato breeding. It was also revealed that chromosome 1 included Verticillium wilt and vine maturity QTL, chromosomes 3 and 10 possessed vine size QTL, and chromosome 2 had an early blight QTL, suggesting additional options for better MAS.

## Data availability statement

The original contributions presented in the study are included in the article/**Supplementary Materials**, further inquiries can be directed to the corresponding author/s.

## Author contributions

All authors contributed to the study conception and design. JP, JW, and RN performed material preparation, field experiments, and data collection in Idaho. In Oregon, VS and SY conducted other field experiments and data collection. JP mainly performed data analysis, statistical analyses, linkage and QTL mapping, and writing the initial draft of the manuscript. All authors commented on previous versions of the manuscript with revisions incorporated. All authors also read and approved the final submitted manuscript.
