# Supplementary material for: Identification of QTL associated with plant vine characteristics and infection response to late blight, early blight, and Verticillium wilt in a tetraploid potato population derived from late blight-resistant Palisade Russet
Source: Front Plant Sci. 2023 Oct 11;14:1222596. doi: 10.3389/fpls.2023.1222596 (PMC10600477; doi:10.3389/fpls.2023.1222596)
Supplement: Supplementary file 1 [file DataSheet_1.zip › DataSheet_1.docx]

**Supplementary Figure 1. The comparison between SNP marker positions of the linkage groups and those of PGSC version 4.03 physical maps**

**
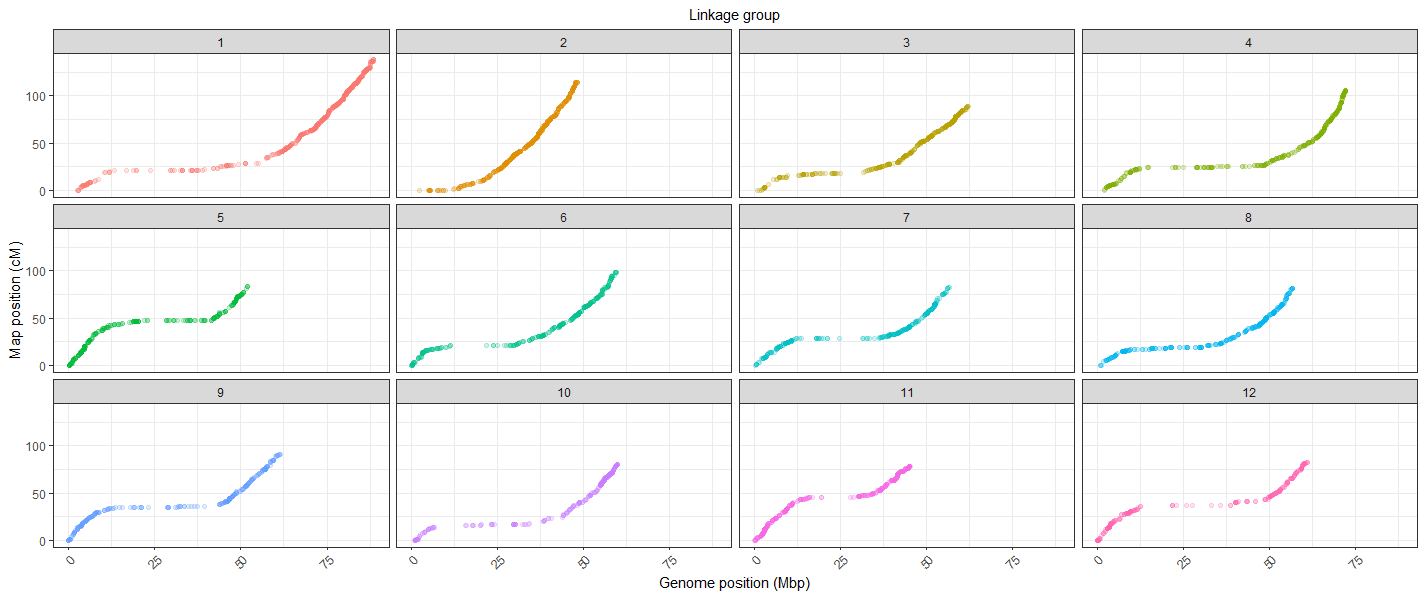
**
