# Supplementary material for: Identification of QTL associated with plant vine characteristics and infection response to late blight, early blight, and Verticillium wilt in a tetraploid potato population derived from late blight-resistant Palisade Russet
Source: Front Plant Sci. 2023 Oct 11;14:1222596. doi: 10.3389/fpls.2023.1222596 (PMC10600477; doi:10.3389/fpls.2023.1222596)
Supplement: Supplementary file 1 [file DataSheet_1.zip › DataSheet_3.docx]

**Supplementary Figure 3. Distribution** **of the BLUP datasets of late blight foliage damage, LB-AUDPC, early blight, Verticillium wilt, vine maturity, and size**

**
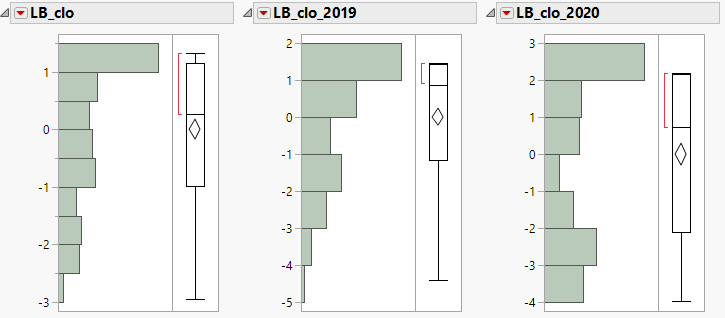

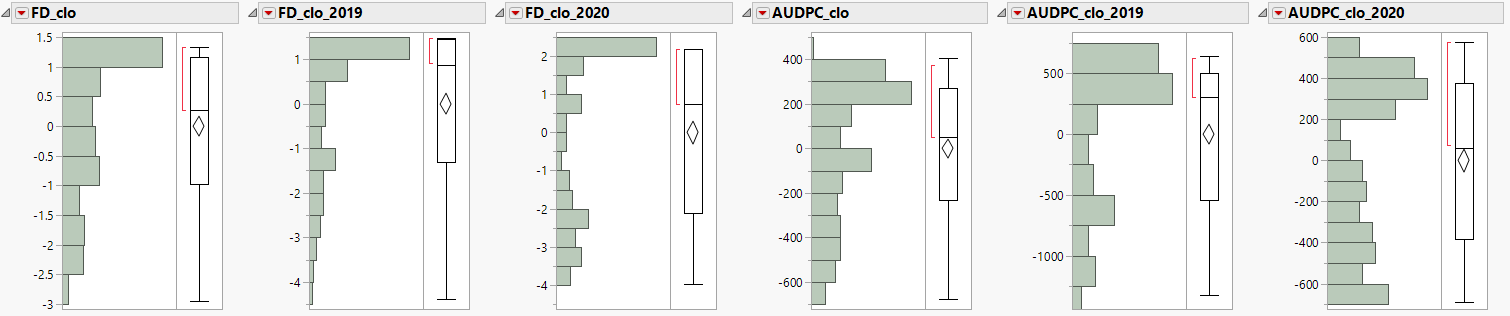
**

**
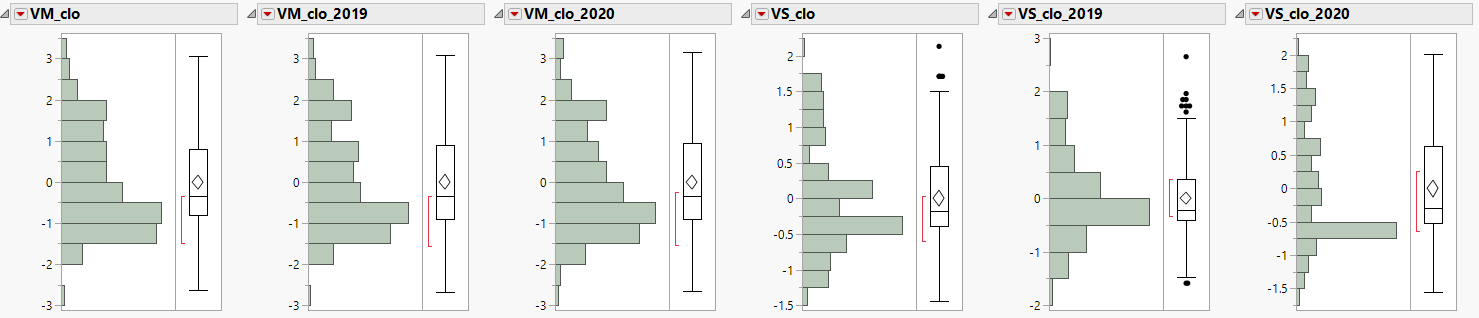
**

**
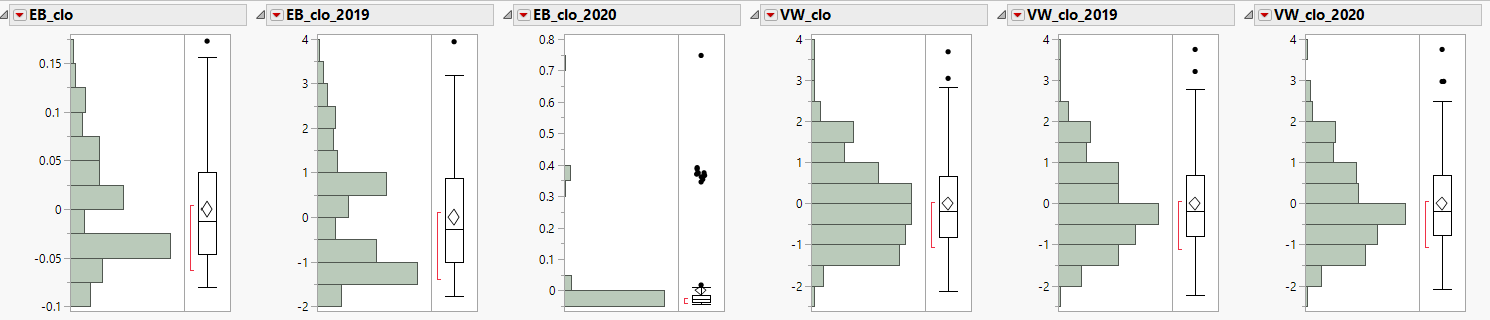

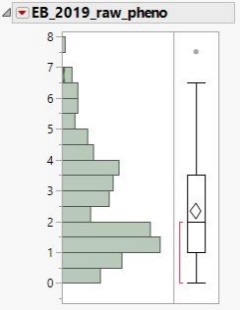
**

BLUP data abbreviations: Late Blight Foliage Damage (LB), Late Blight Area Under the Disease Progress Curve (LB-AUDPC), Early Blight resistance (EB), Verticillium Wilt resistance (VW), Vine Maturity (VM), Vine Size (VS), a genetic effect of clones (clo), 2019 (2019), and 2020 (2020) year effects.

The distribution patterns of the six traits were arranged here. Unlike the other five traits, the 2020 EB damage phenotype data resulted in an almost ignorable segregation pattern; thus, the average of two replicates of the raw 2019 early blight damage phenotype data (*EB_2019_raw_pheno*) was presented.
