# Supplementary material for: Identification of QTL associated with plant vine characteristics and infection response to late blight, early blight, and Verticillium wilt in a tetraploid potato population derived from late blight-resistant Palisade Russet
Source: Front Plant Sci. 2023 Oct 11;14:1222596. doi: 10.3389/fpls.2023.1222596 (PMC10600477; doi:10.3389/fpls.2023.1222596)

Supplementary Fig. 4. Allele effects of the significant QTL positions

- The "a" to "d" at the X-axes in the following bar graphs represents four phased homologs of Palisade Russet, and the "e" to "h" represents another four homologs of ND028673B-2Russ. The Y axes in the same bar graphs indicate each homolog's contribution to the average of the whole mapping population. The qtl_effects function of QTLpoly calculated an estimate of each allele effect across the eight homologs; thus, all the Y axes in Supplementary Figure 4 were labeled as “Estimate” without any unit. Through those bar graphs, it was possible to quantify how much each homolog of each parent adds to or subtracts from the mean of the 190 progenies, revealing which allele(s) among the eight parental homologs most significantly impact a trait.
- BLUP data abbreviations: Late Blight Foliage Damage (LB), Late Blight Area Under the Disease Progress Curve (LB-AUDPC), Early Blight resistance (EB), Verticillium Wilt resistance (VW), Vine Maturity (VM), Vine Size (VS), a genetic effect of clones (clo), 2019 (2019), and 2020 (2020) year effects.
- The details of these BLUP datasets are described in Supplementary Table 5.
- Unlike the other traits, the 2019 raw early blight damage data were subjected to QTL analysis of EB.

LB_clo_ch5

SNP (Location): PotVar0077880 (17.09 cM)
Chromosome: chr05
BLUP data: *LB_clo*

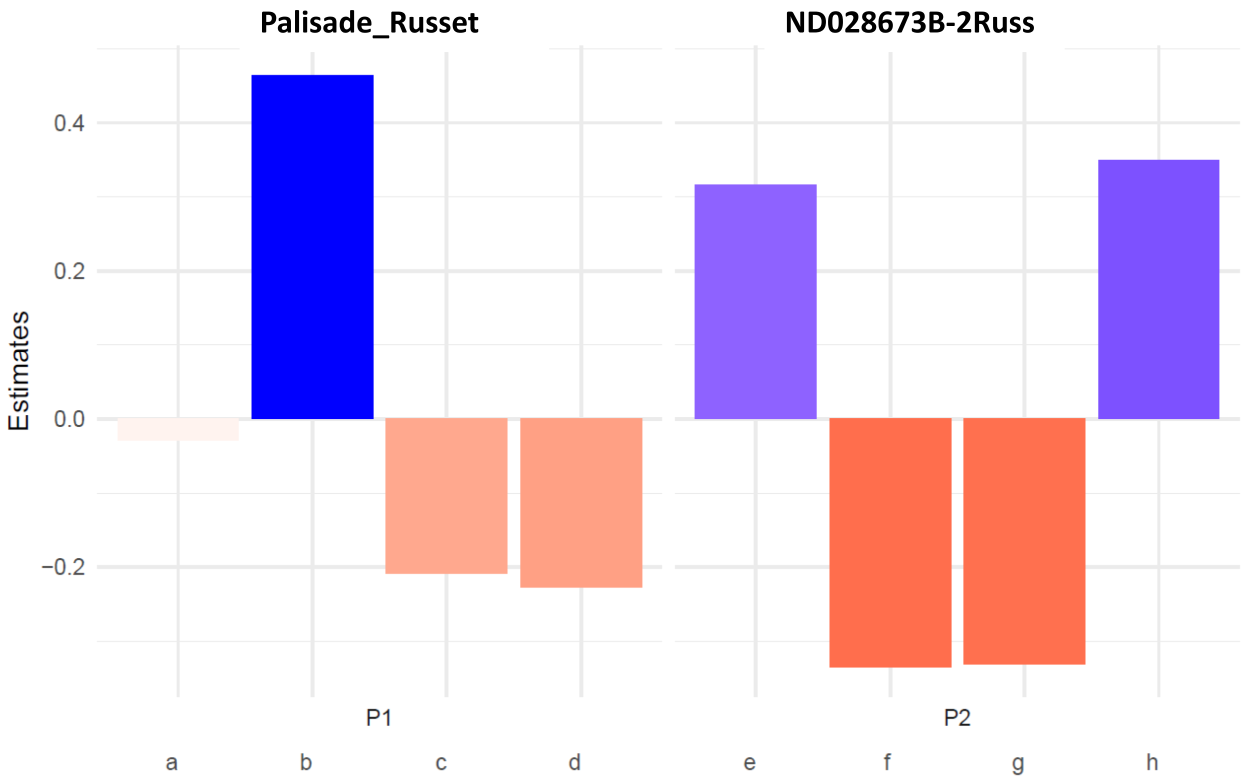

LB_clo_2019_ch5

SNP (Location): PotVar0077880 (17.09 cM)
Chromosome: chr05
BLUP data: *LB_clo_2019*

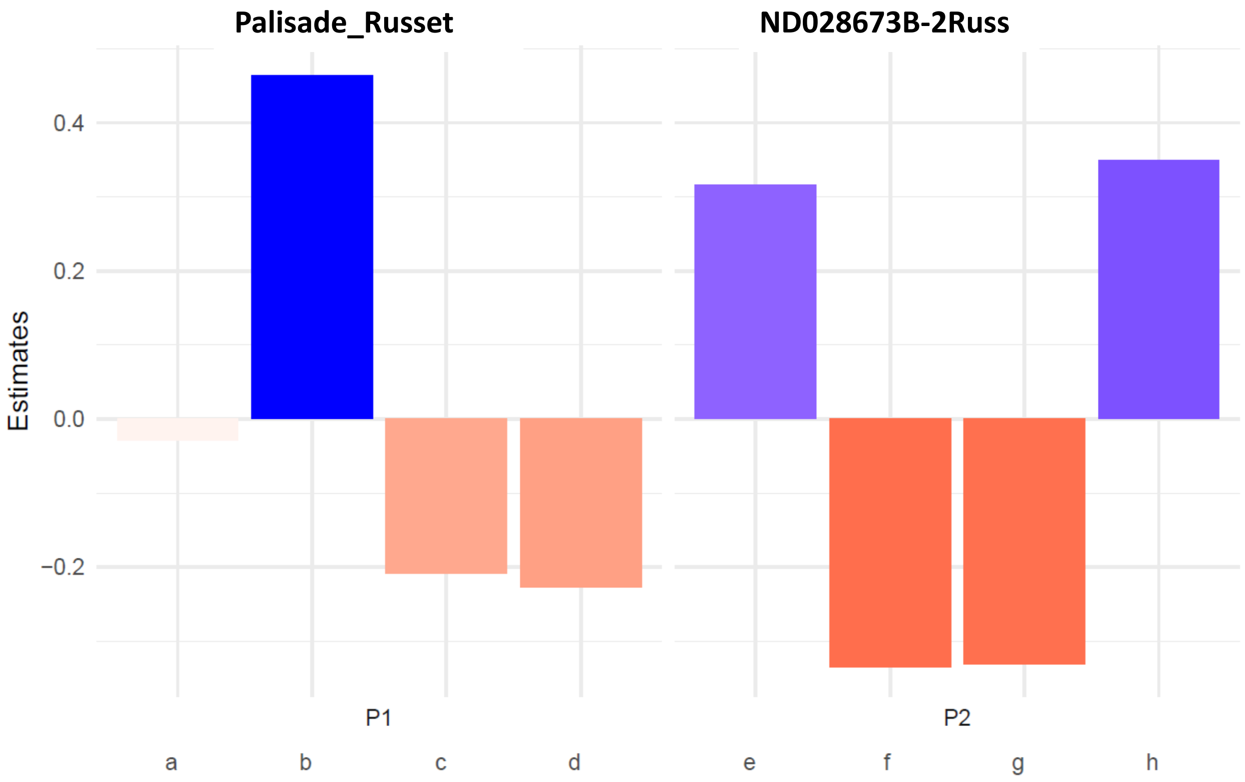

LB_clo_2020_ch5

SNP (Location): PotVar0077880 (17.09 cM)
Chromosome: chr05
BLUP data: *LB_clo_2020*

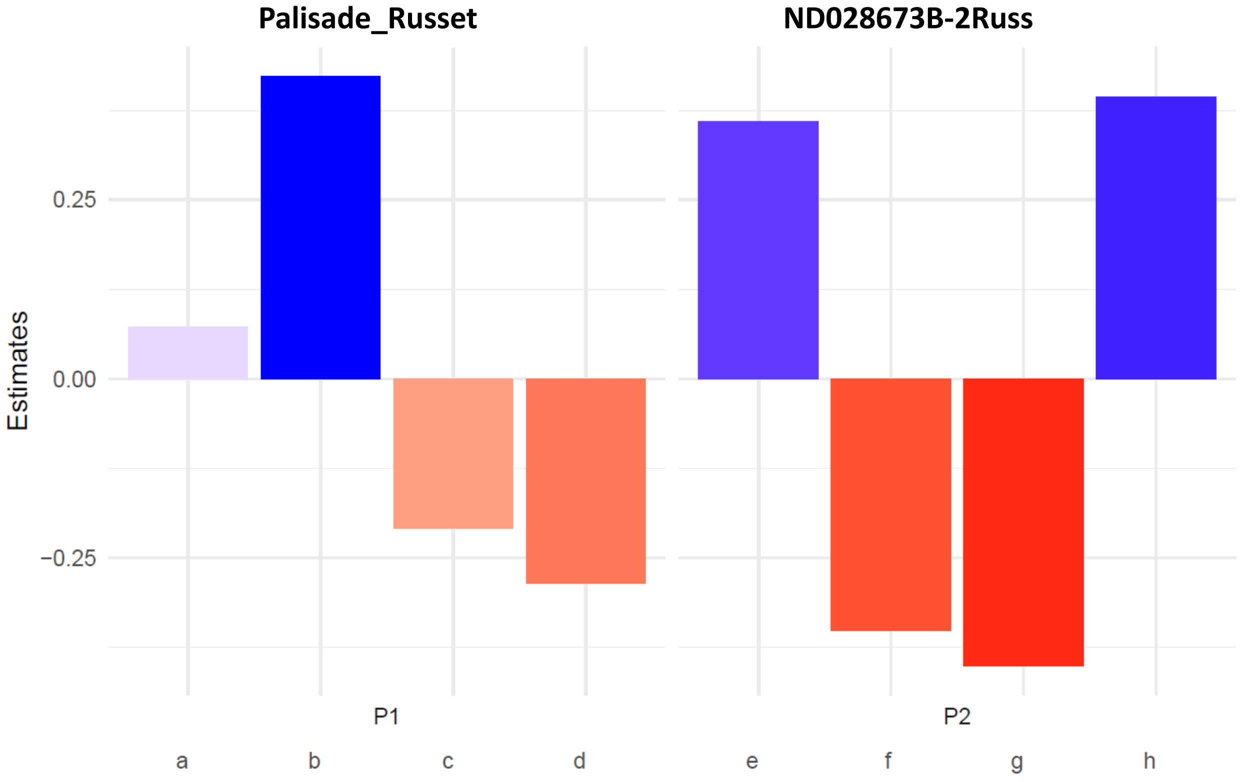

LB-AUDPC_clo_ch5

SNP (Location): PotVar0077880 (17.09 cM)
Chromosome: chr05
BLUP data: *LB-AUDPC_clo*

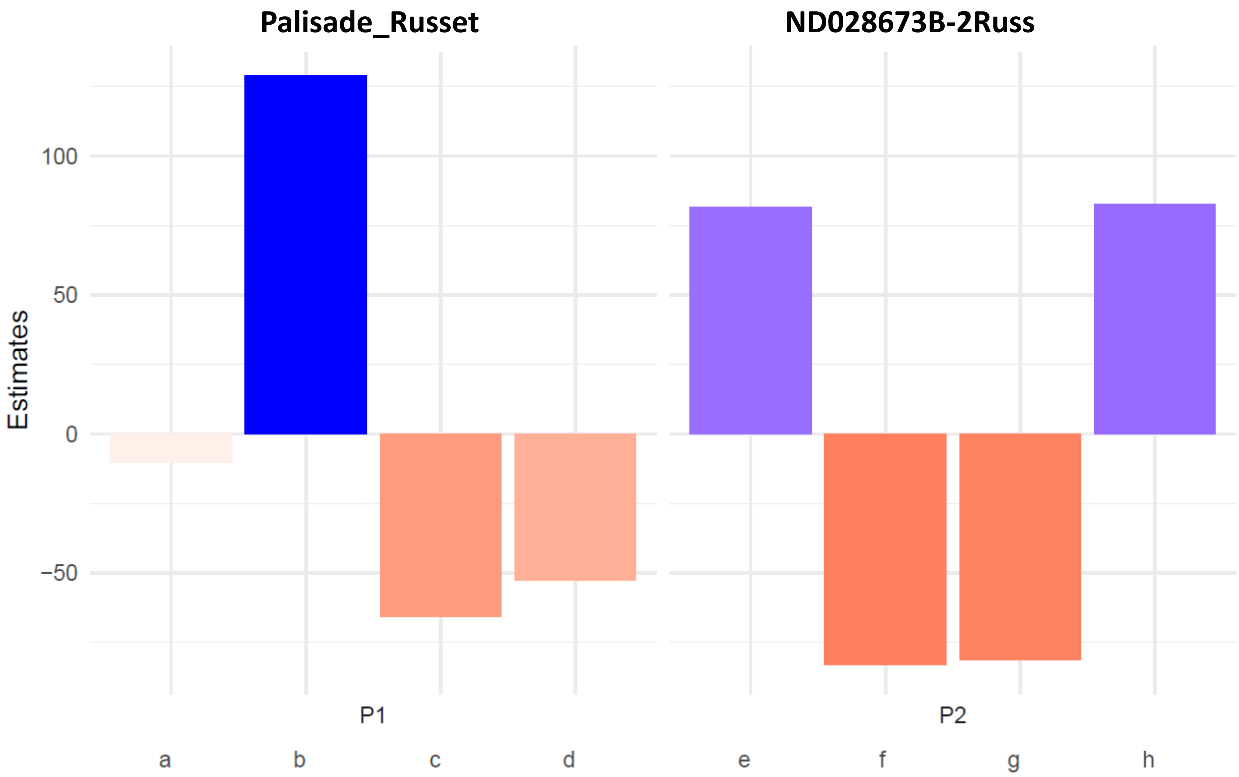

LB-AUDPC_clo_2019_ch5

SNP (Location): PotVar0077880 (17.09 cM)
Chromosome: chr05
BLUP data: *LB-AUDPC_clo_2019*

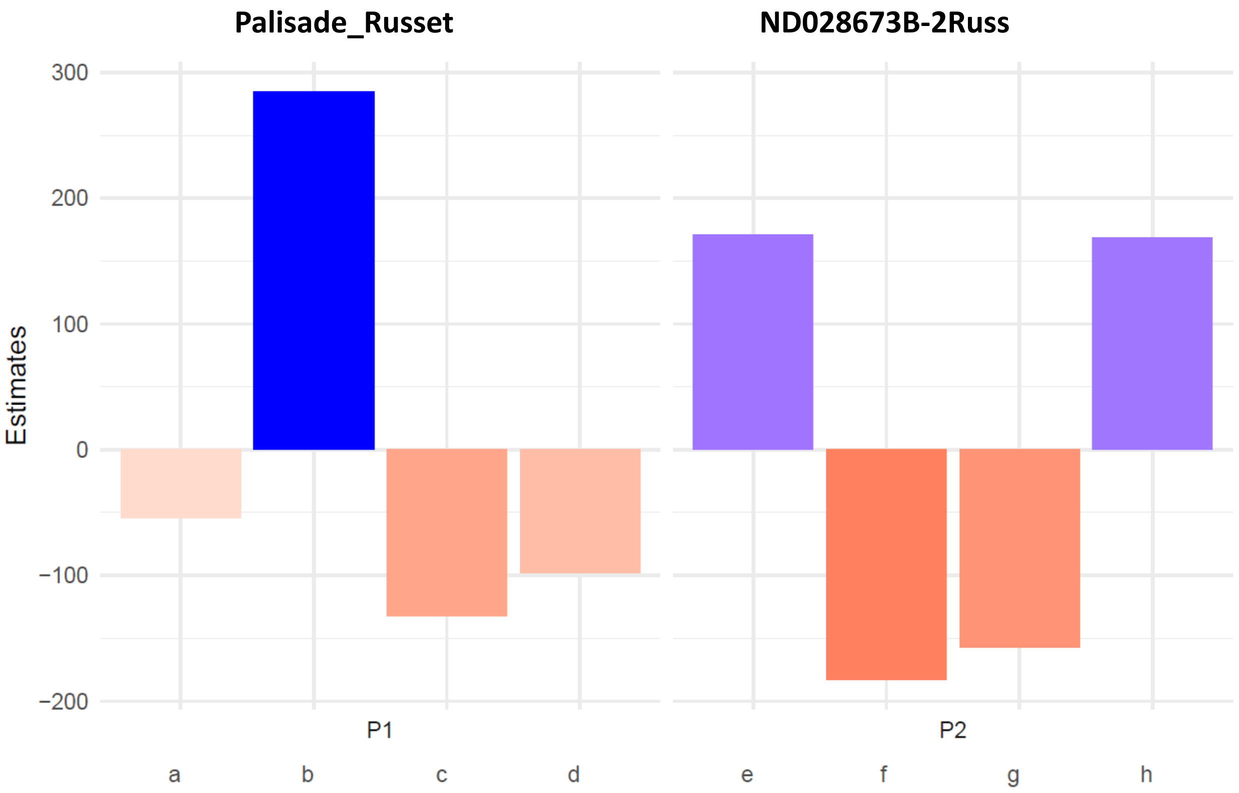

LB-AUDPC_clo_2020_ch5

SNP (Location): PotVar0077880 (17.09 cM)
Chromosome: chr05
BLUP data: *LB-AUDPC_clo_2020*

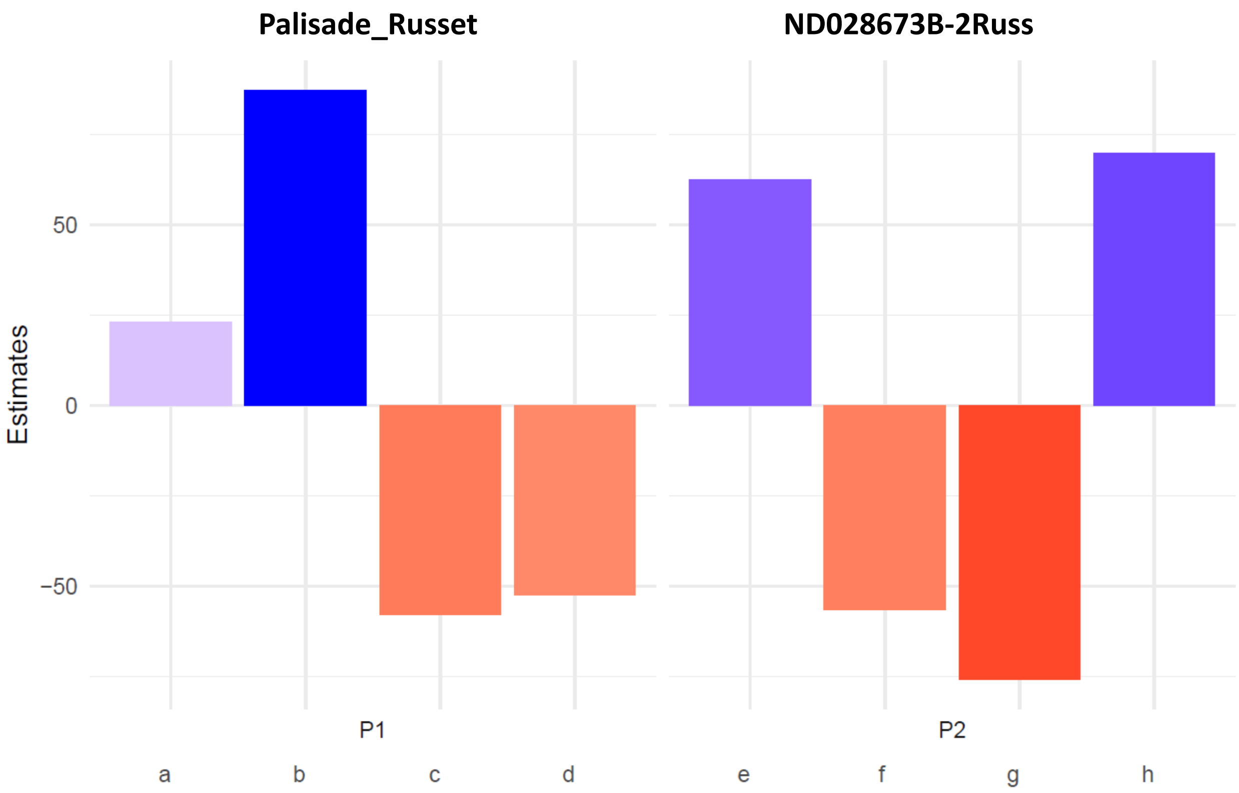

VW_clo_ch1

SNP (Location): solcap_snp_c2_37574 (54.07 cM)
Chromosome: chr01
BLUP data: *VW_clo*

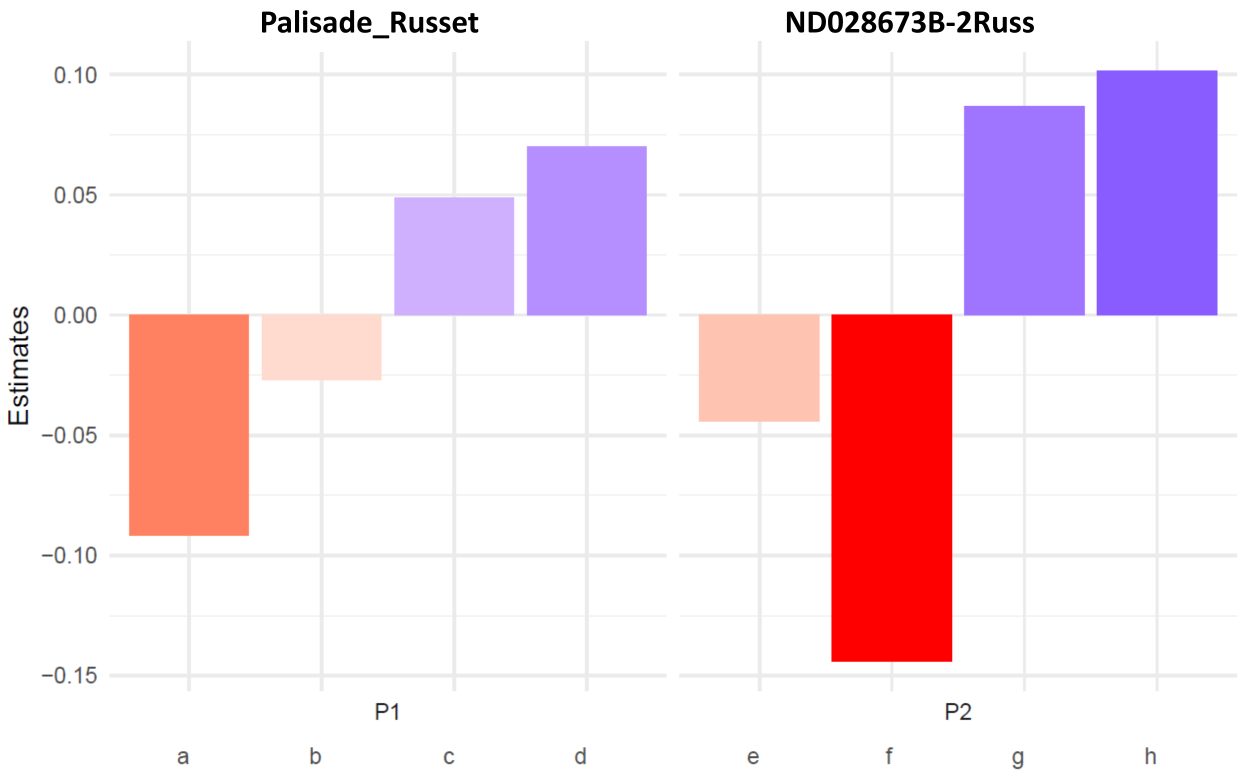

VW_clo_2019_ch1

SNP (Location): solcap_snp_c2_37574 (54.07 cM)
Chromosome: chr01
BLUP data: *VW_clo_2019*

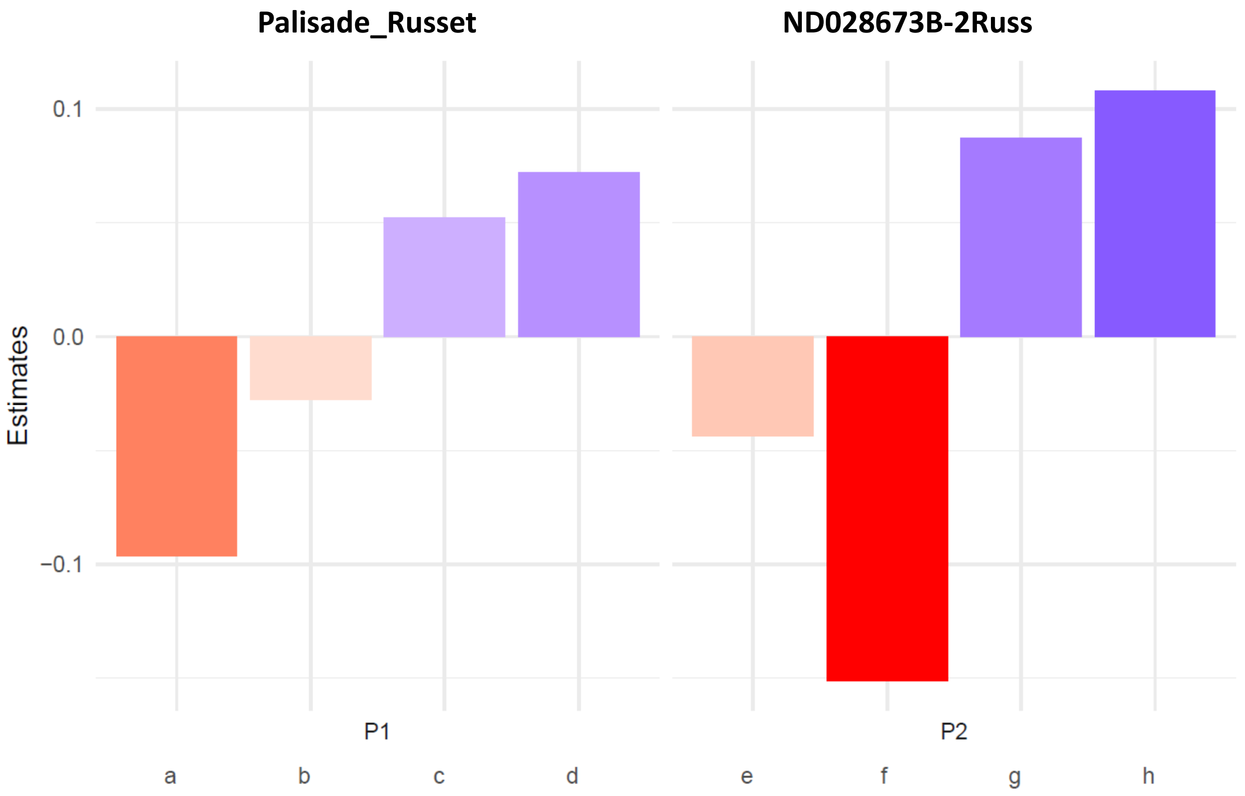

VW_clo_2020_ch1

SNP (Location): solcap_snp_c2_37574 (54.07 cM)
Chromosome: chr01
BLUP data: *VW_clo_2020*

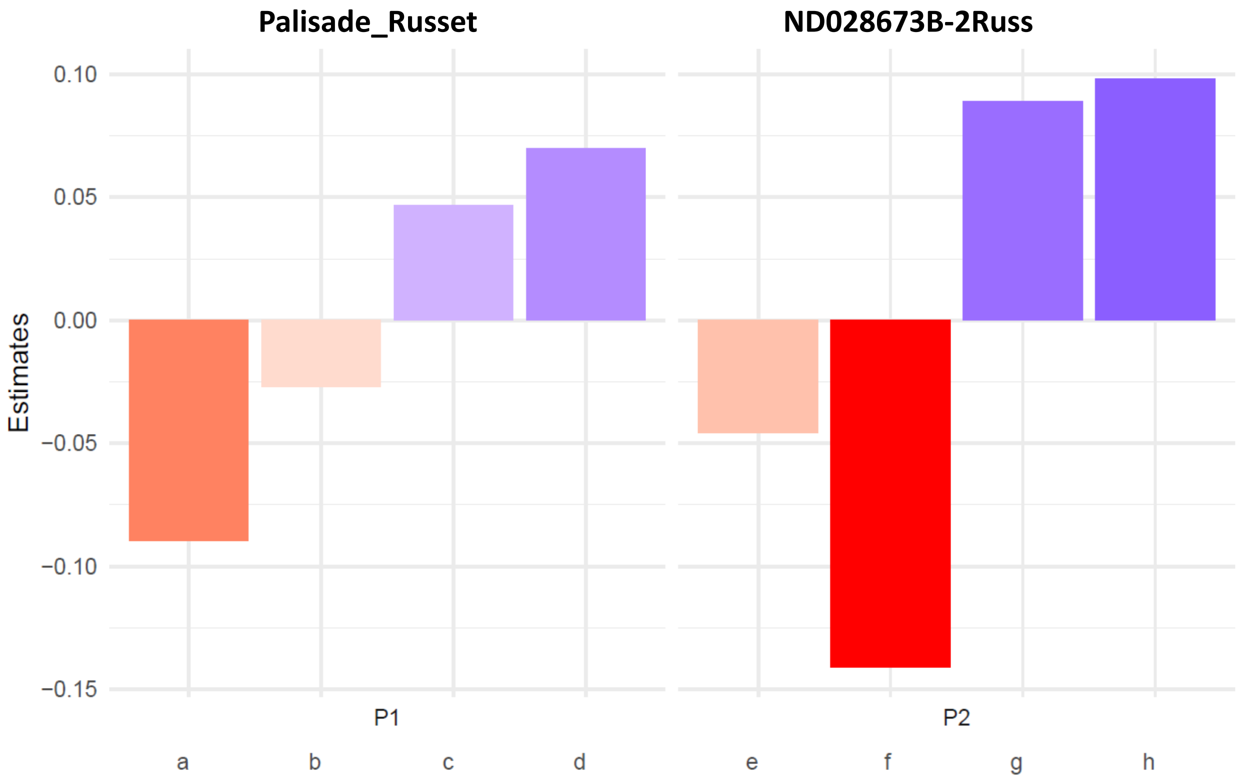

VW_clo_ch5

SNP (Location): PotVar0026113 (16.54 cM)
Chromosome: chr05
BLUP data: *VW_clo*

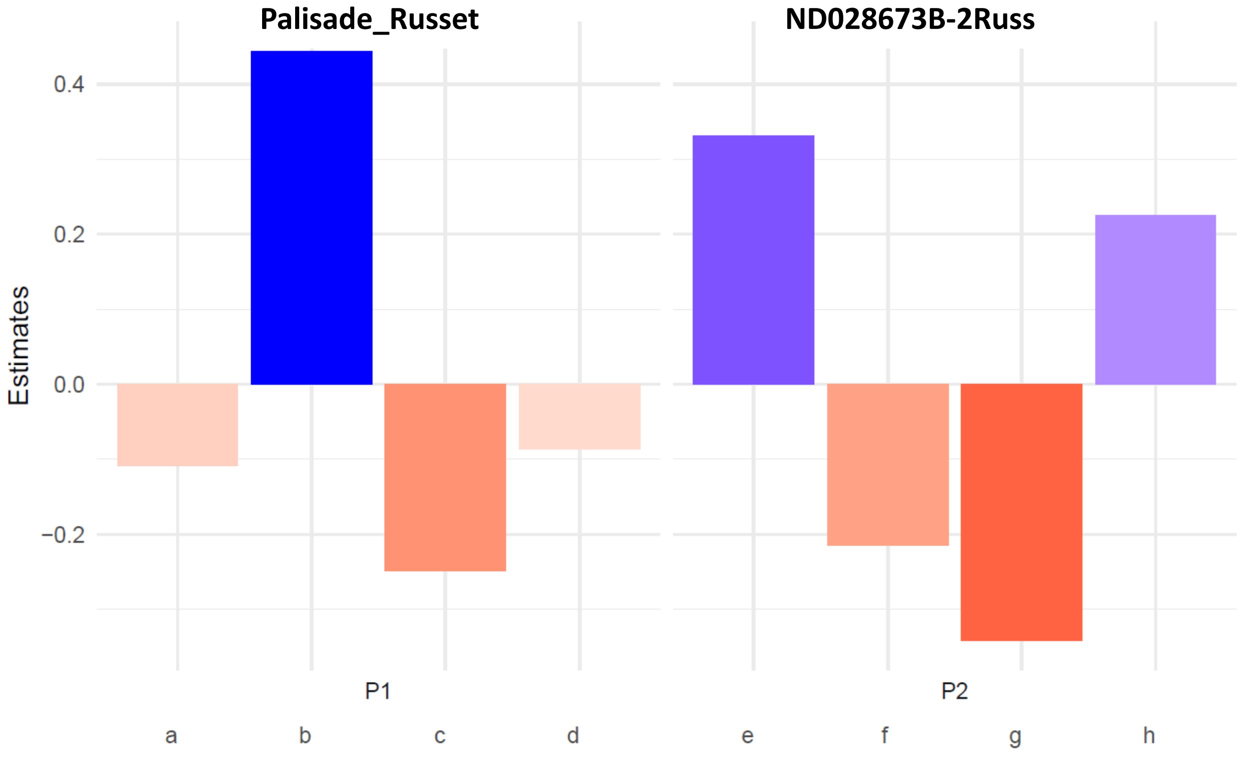

VW_clo_2019_ch5

SNP (Location): PotVar0026113 (16.54 cM)
Chromosome: chr05
BLUP data: *VW_clo_2019*

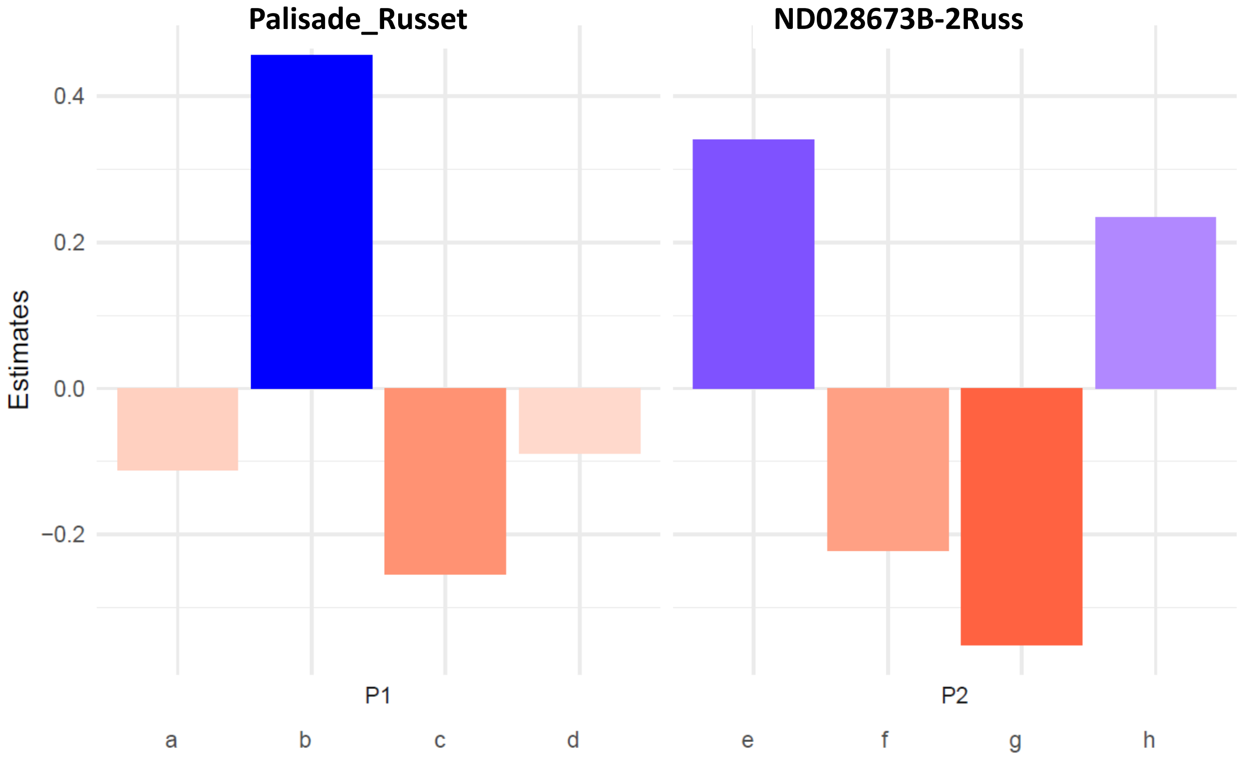

VW_clo_2020_ch5

SNP (Location): PotVar0026113 (16.54 cM)
Chromosome: chr05
BLUP data: *VW_clo_2020*

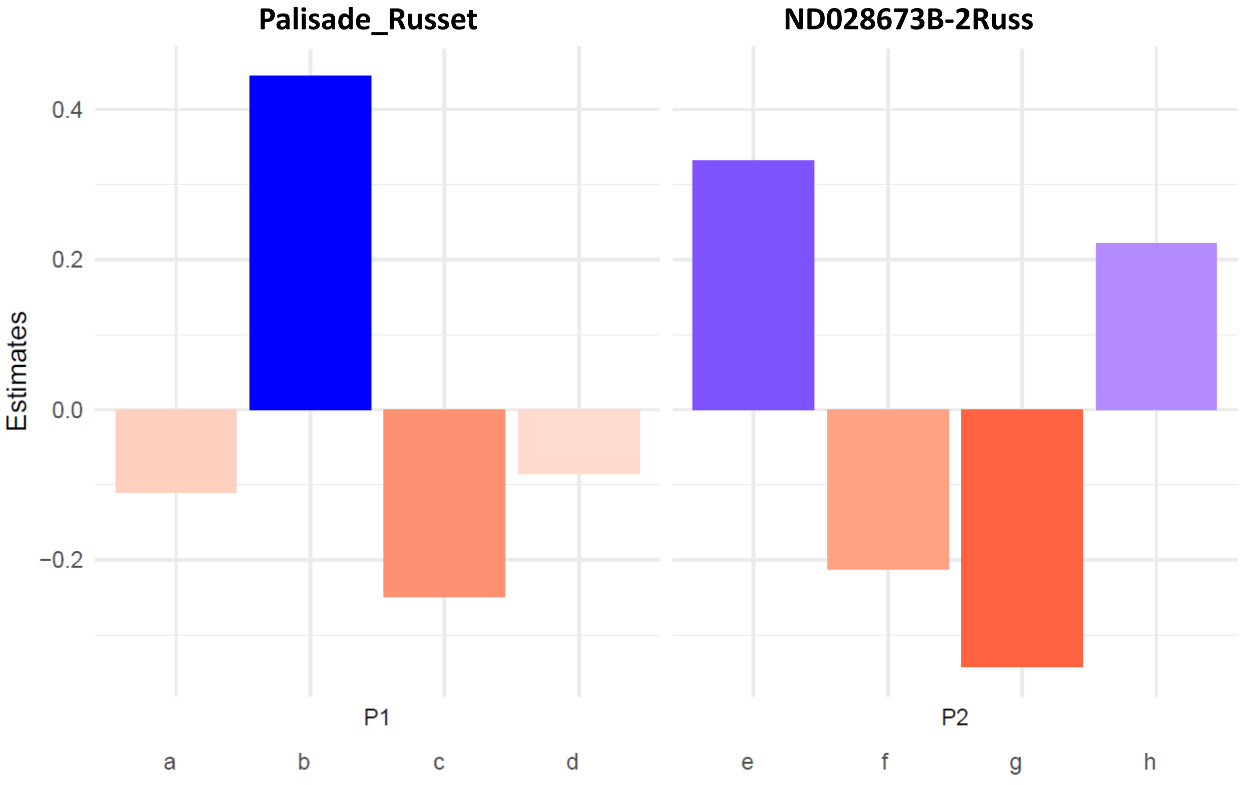

VM_clo_ch1

SNP (Location): solcap_snp_c1_6288 (73.16 cM)
Chromosome: chr01
BLUP data: *VM_clo*

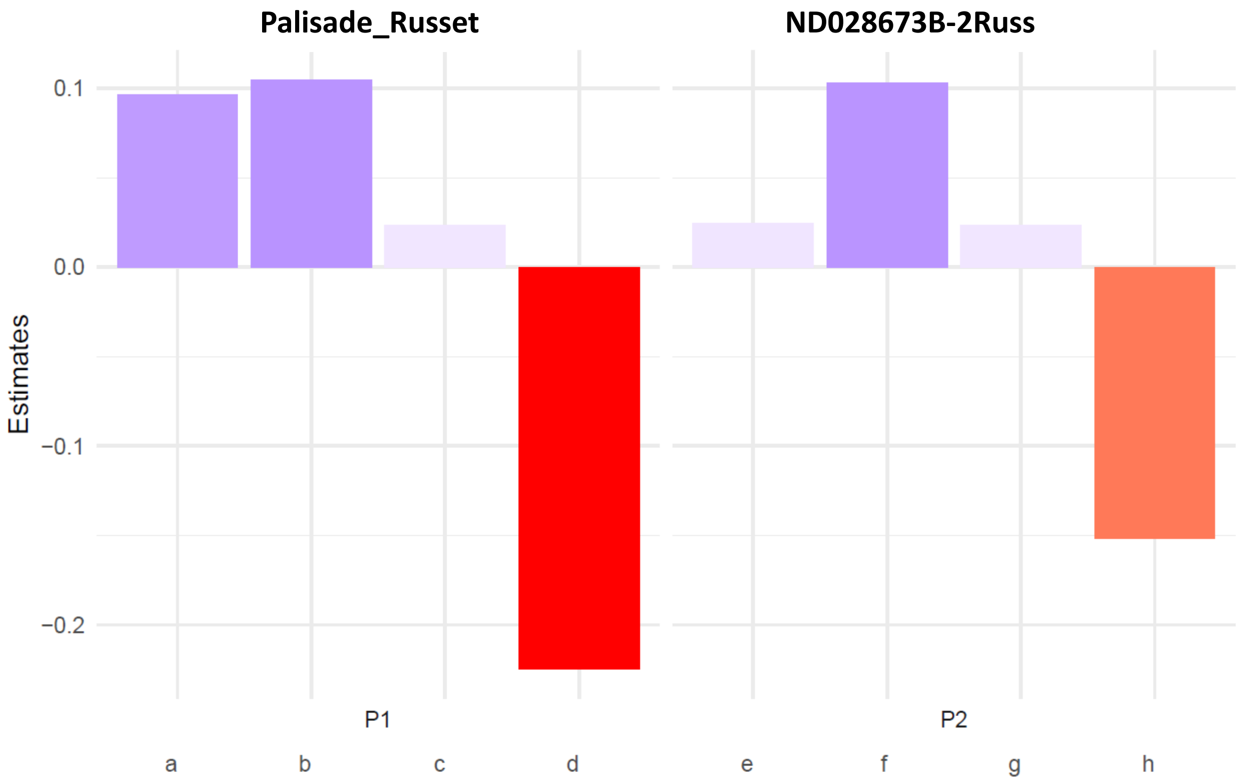

VM_clo_2019_ch1

SNP (Location): solcap_snp_c1_6288 (73.16 cM)
Chromosome: chr01
BLUP data: *VM_clo_2019*

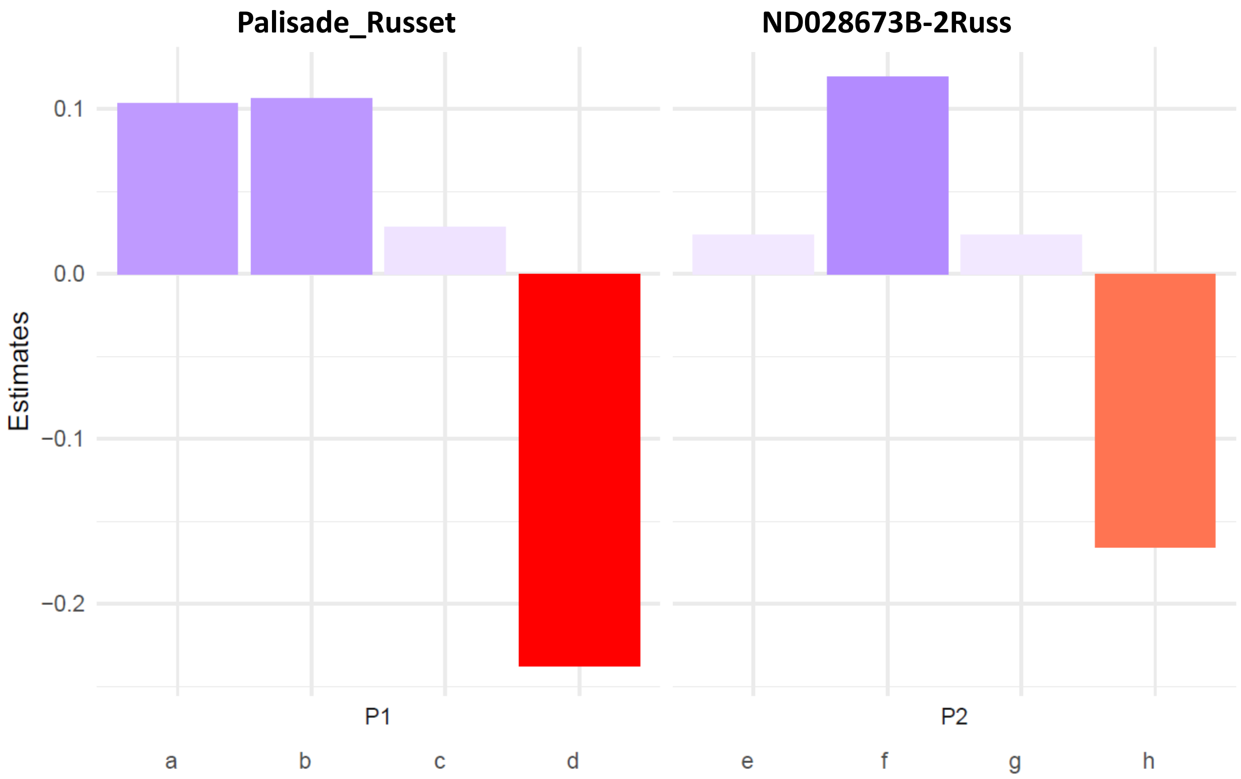

VM_clo_2020_ch1

SNP (Location): solcap_snp_c1_6288 (73.16 cM)
Chromosome: chr01
BLUP data: *VM_clo_2020*

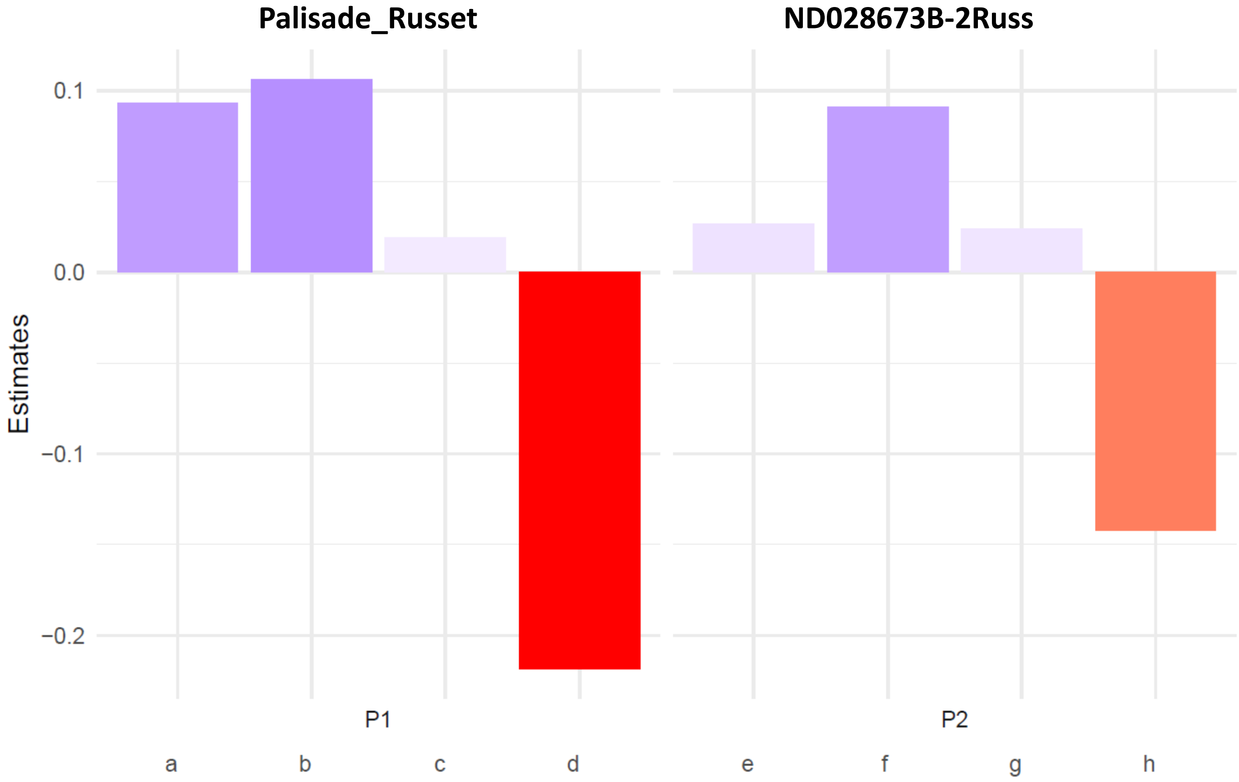

VM_clo_ch5 SNP (Location): PotVar0026113 (16.54 cM)
Chromosome: chr05
BLUP data: *VM_clo*

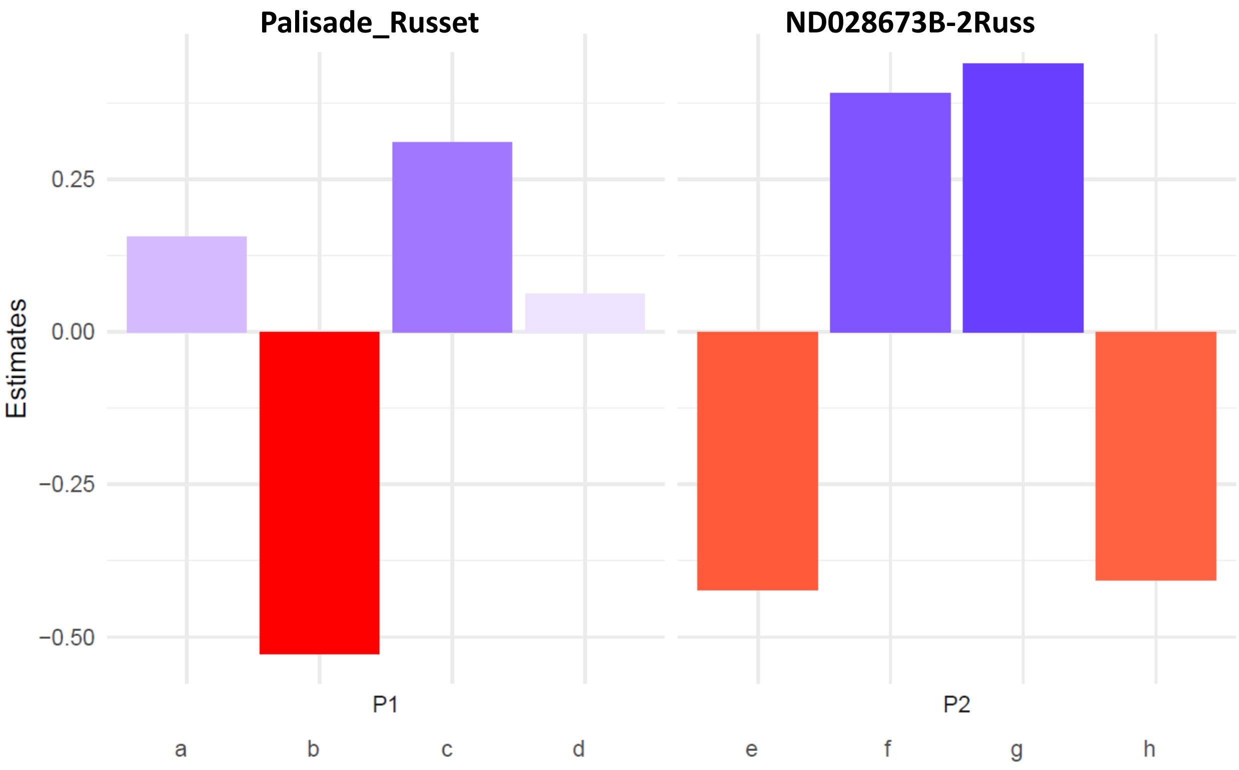

VM_clo_2019_ch5 SNP (Location): PotVar0026113 (16.54 cM)
Chromosome: chr05
BLUP data: *VM_clo_2019*

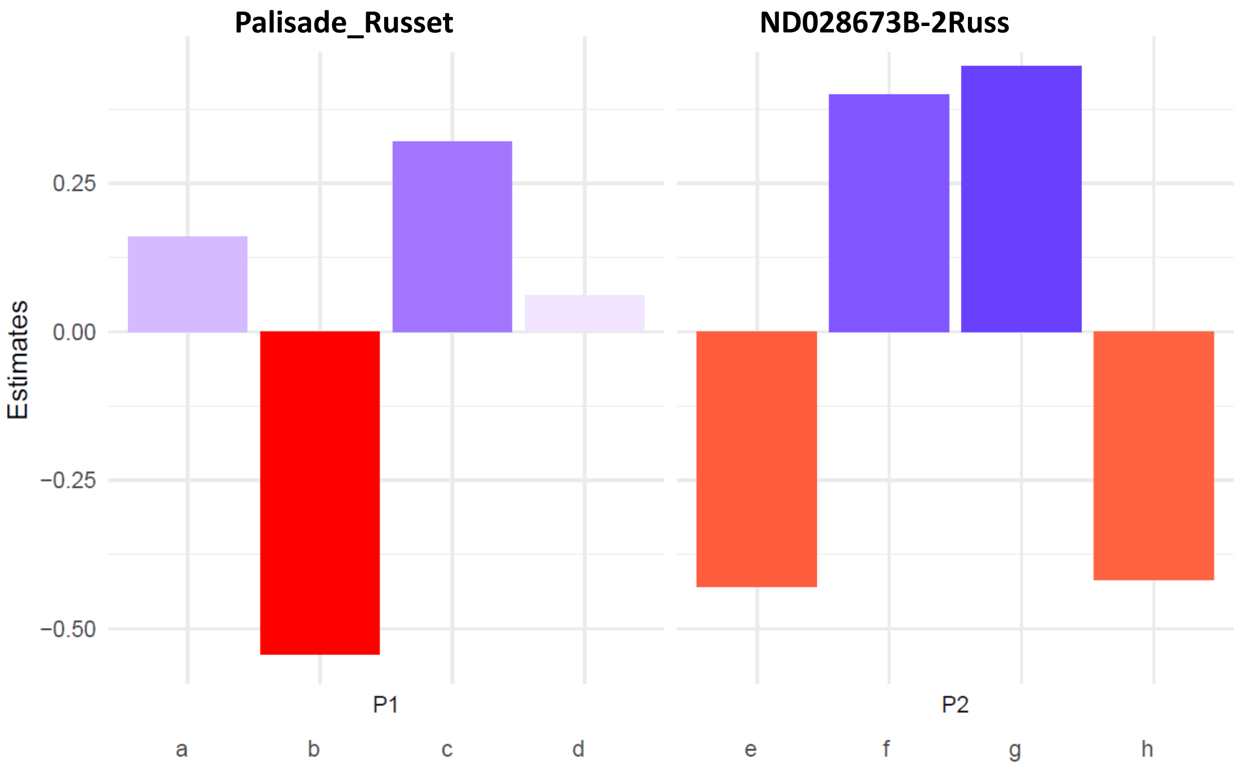

VM_clo_2020_ch5 SNP (Location): PotVar0026113 (16.54 cM)
Chromosome: chr05
BLUP data: *VM_clo_2020*

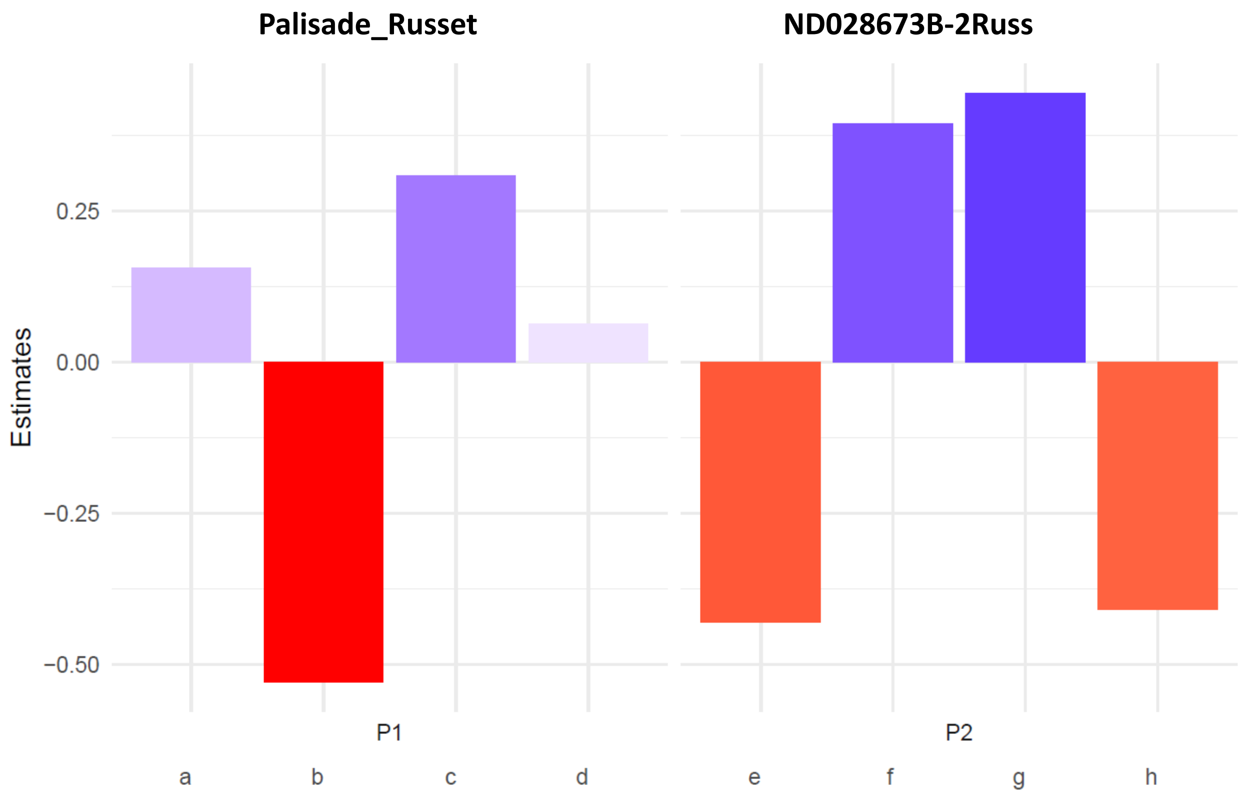

VS_clo_ch3 SNP (Location): PotVar0120301 (50.37 cM)
Chromosome: chr03
BLUP data: *VS_clo*

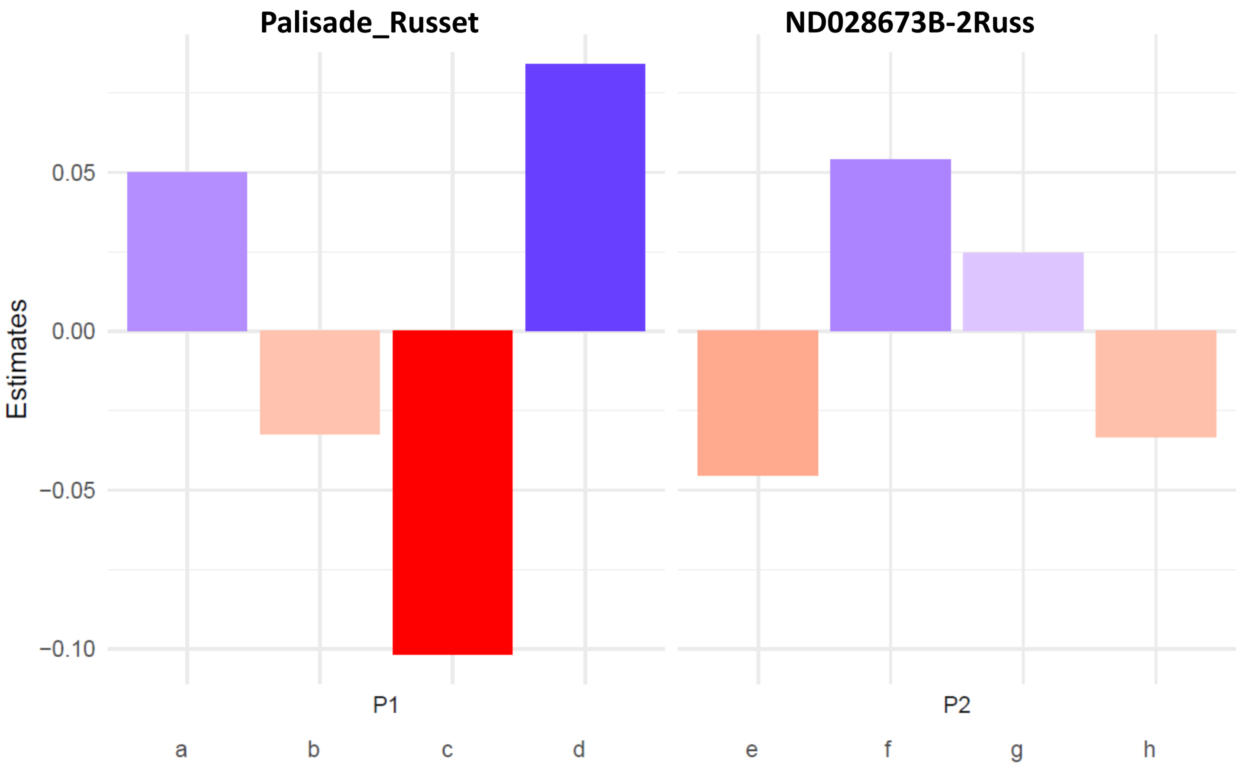

VS_clo_2019_ch3 SNP (Location): PotVar0120301 (50.37 cM)
Chromosome: chr03
BLUP data: *VS_clo_2019*

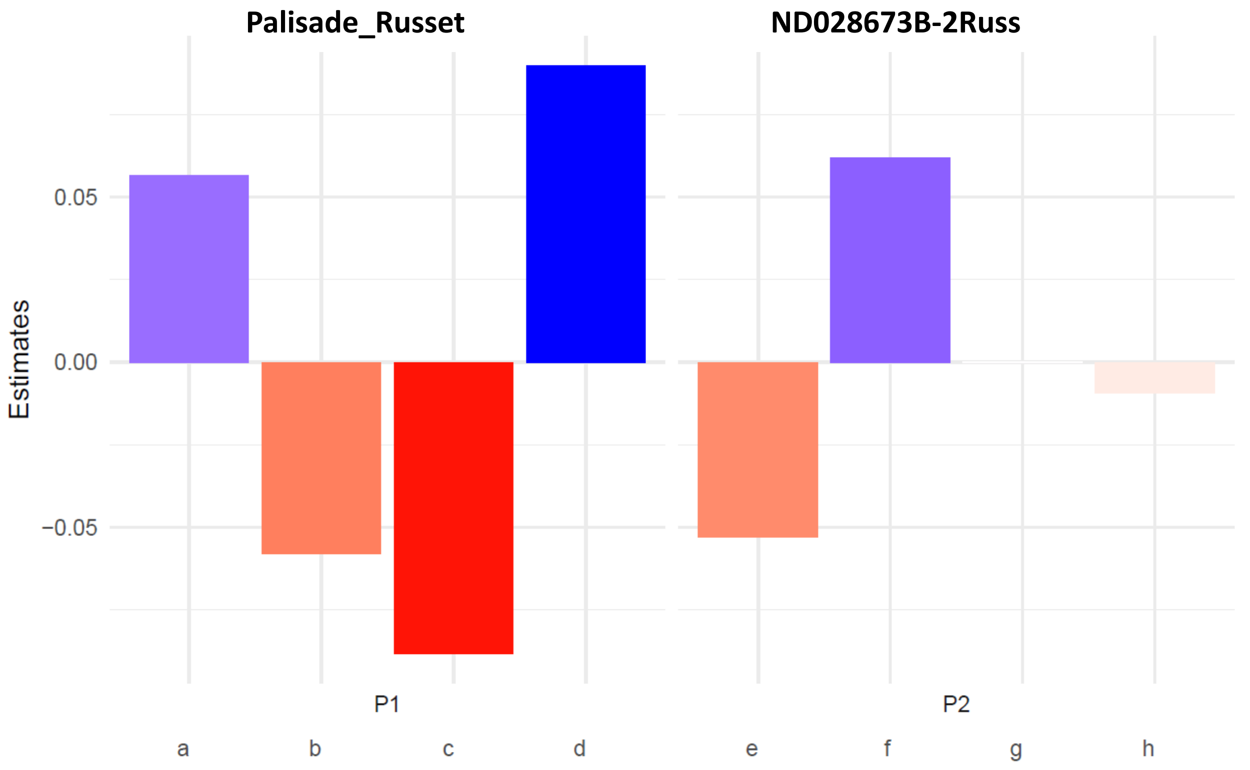

VS_clo_2020_ch3 SNP (Location): PotVar0120301 (50.37 cM)
Chromosome: chr03
BLUP data: *VS_clo_2020*

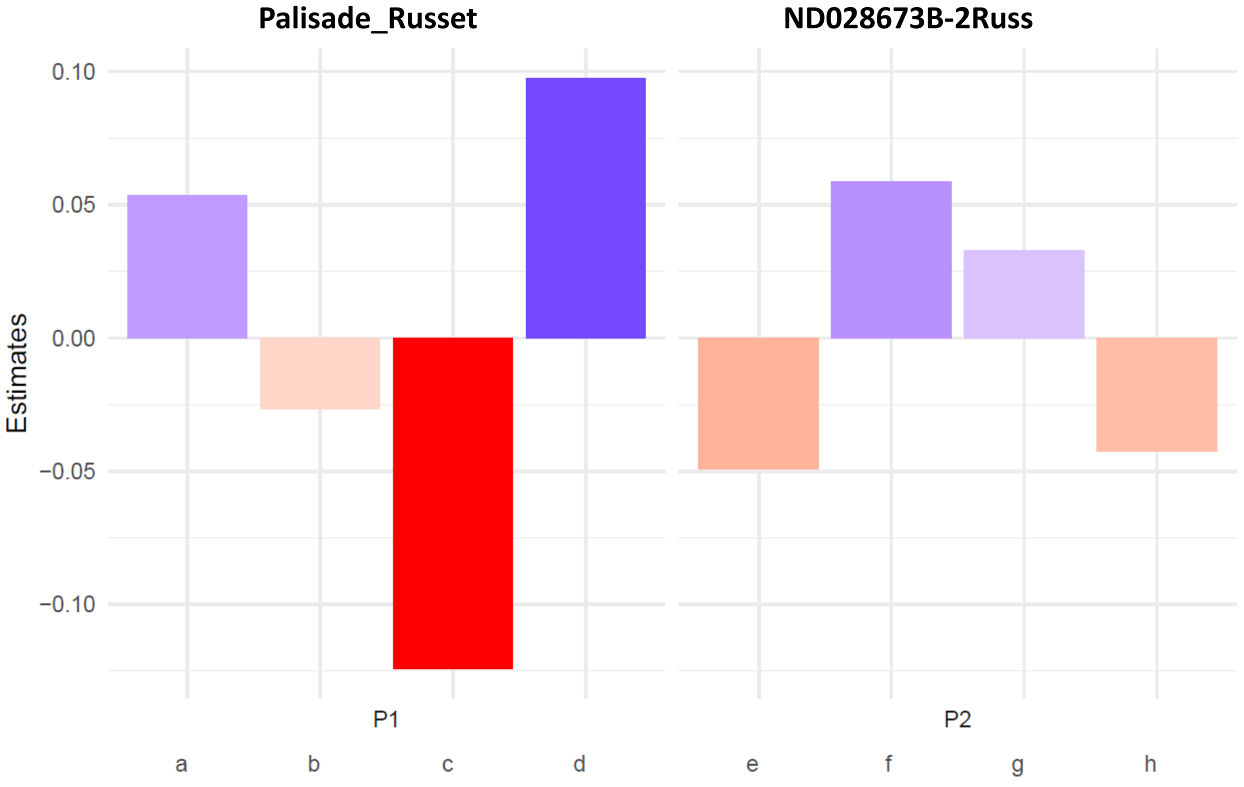

VS_clo_ch5

SNP (Location): PotVar0077880 (17.09 cM)
Chromosome: chr05
BLUP data: *VS_clo*

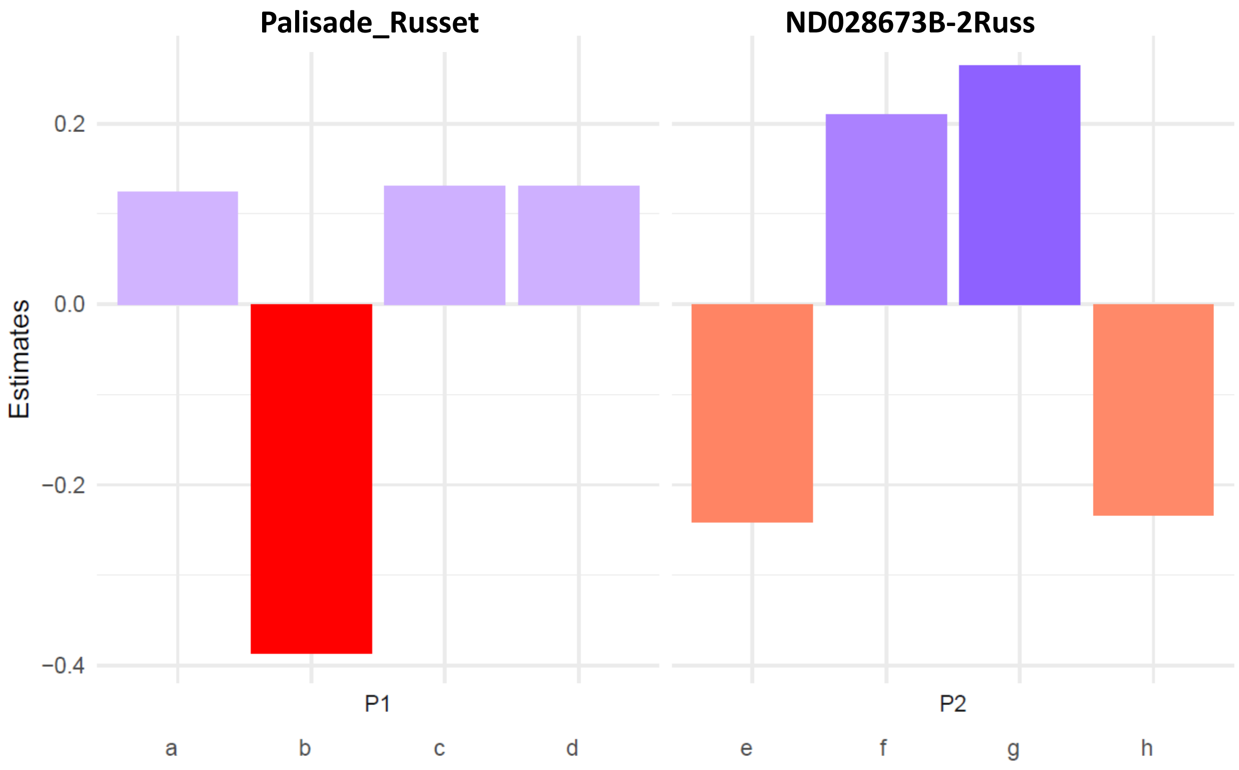

VS_clo_2019_ch5

SNP (Location): PotVar0026113 (16.54 cM)
Chromosome: chr05
BLUP data: *VS_clo_2019*

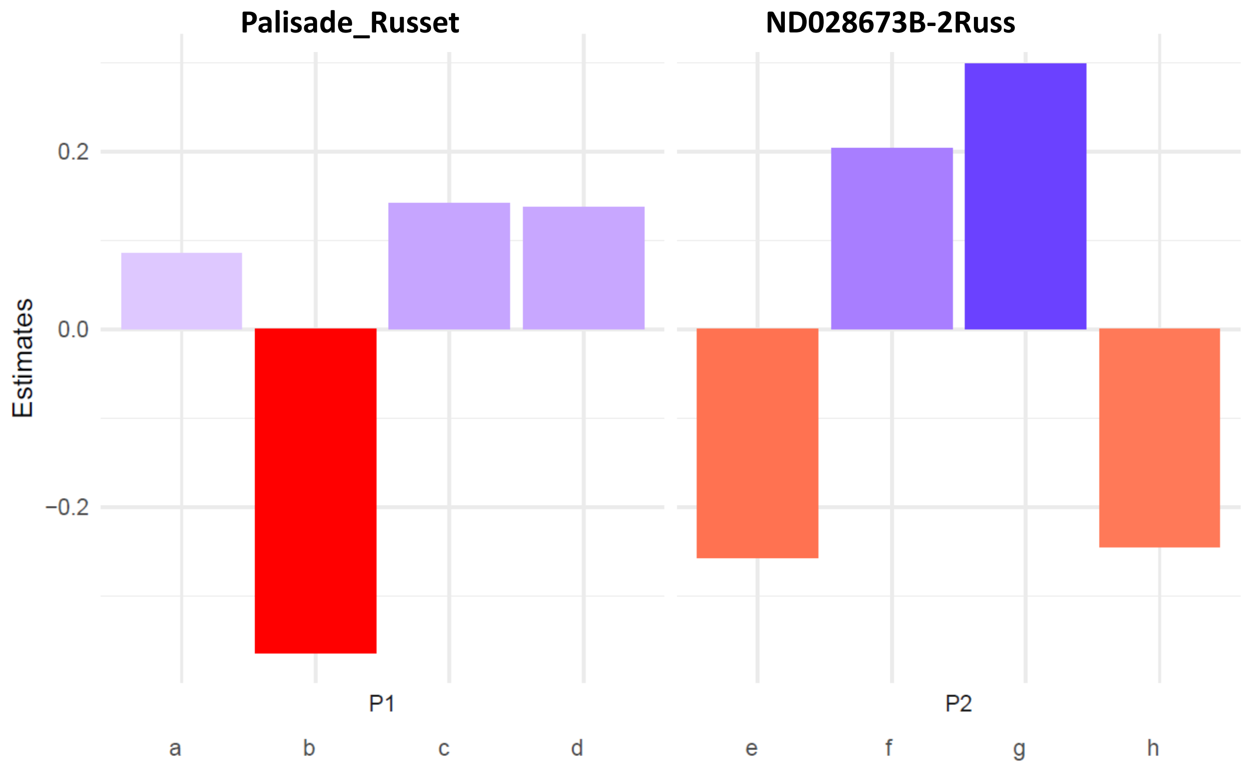

VM_clo_2020_ch5

SNP (Location): PotVar0077880 (17.09 cM)
Chromosome: chr05
BLUP data: *VS_clo_2020*

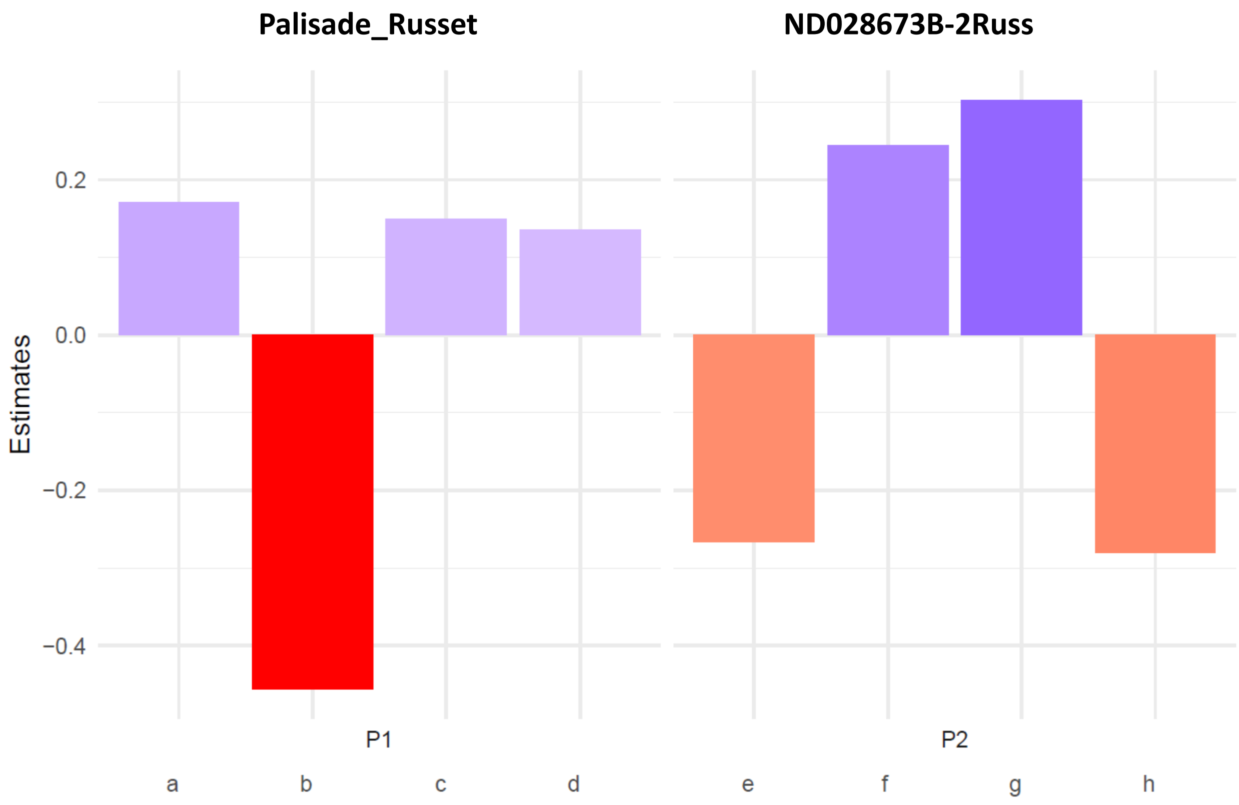

VS_clo_ch10 SNP (Location): solcap_snp_c2_48127 (64.49 cM)
Chromosome: chr10
BLUP data: *VS_clo*

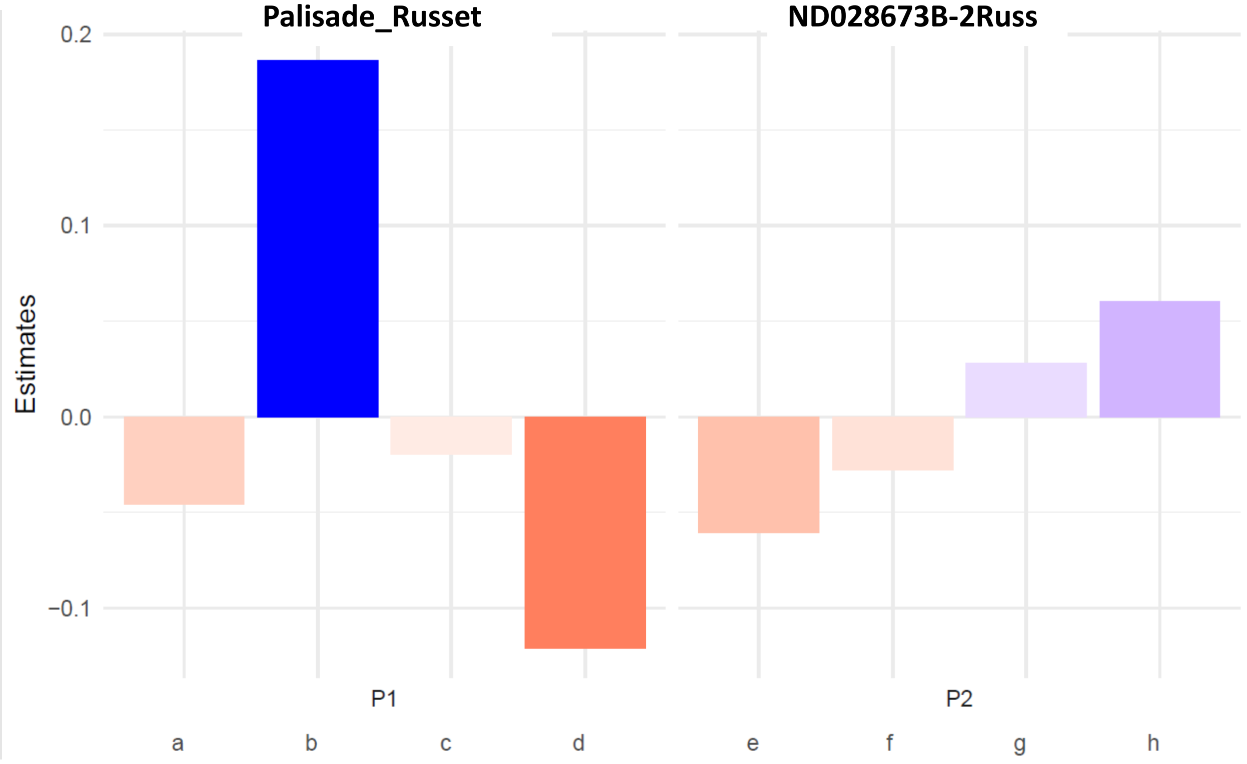

VS_clo_2019_ch10 SNP (Location): solcap_snp_c2_22594 (69.09 cM)
Chromosome: chr10
BLUP data: *VS_clo_2019*

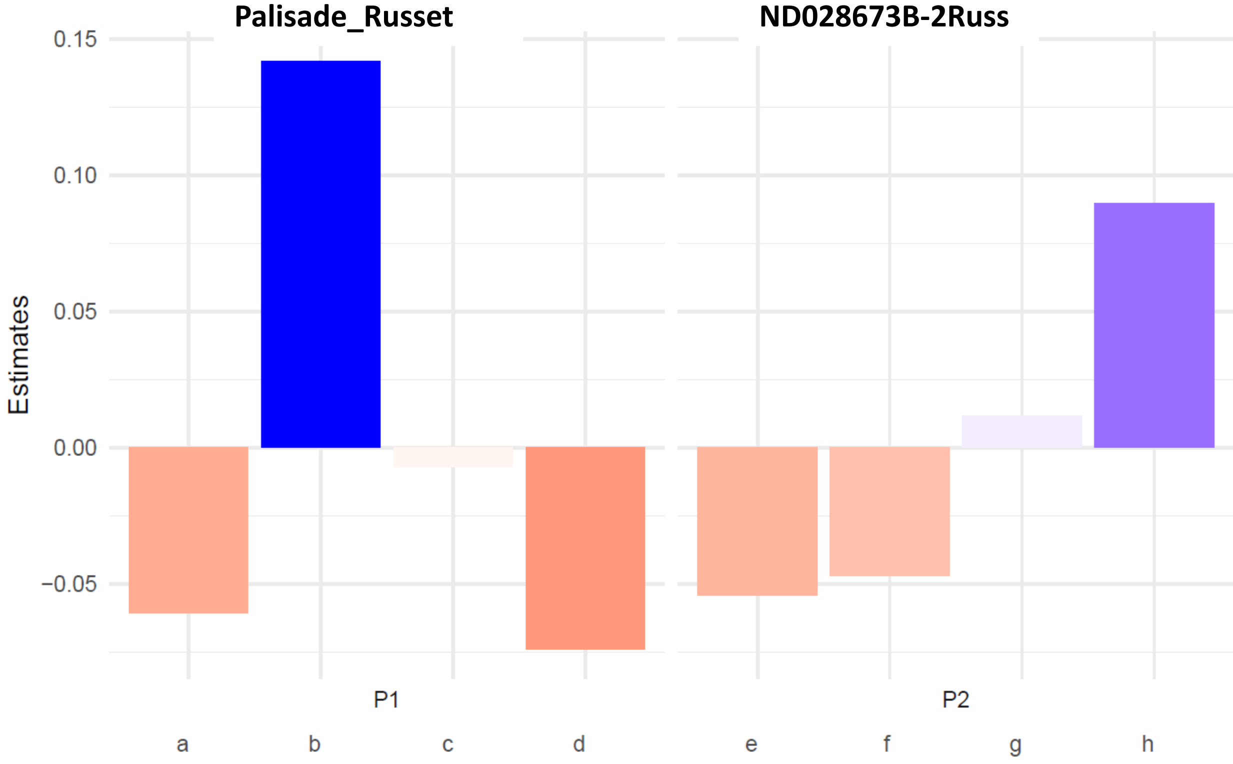

VS_clo_2020_ch10 SNP (Location): solcap_snp_c2_48127 (64.49 cM)
Chromosome: chr10
BLUP data: *VS_clo_2020*

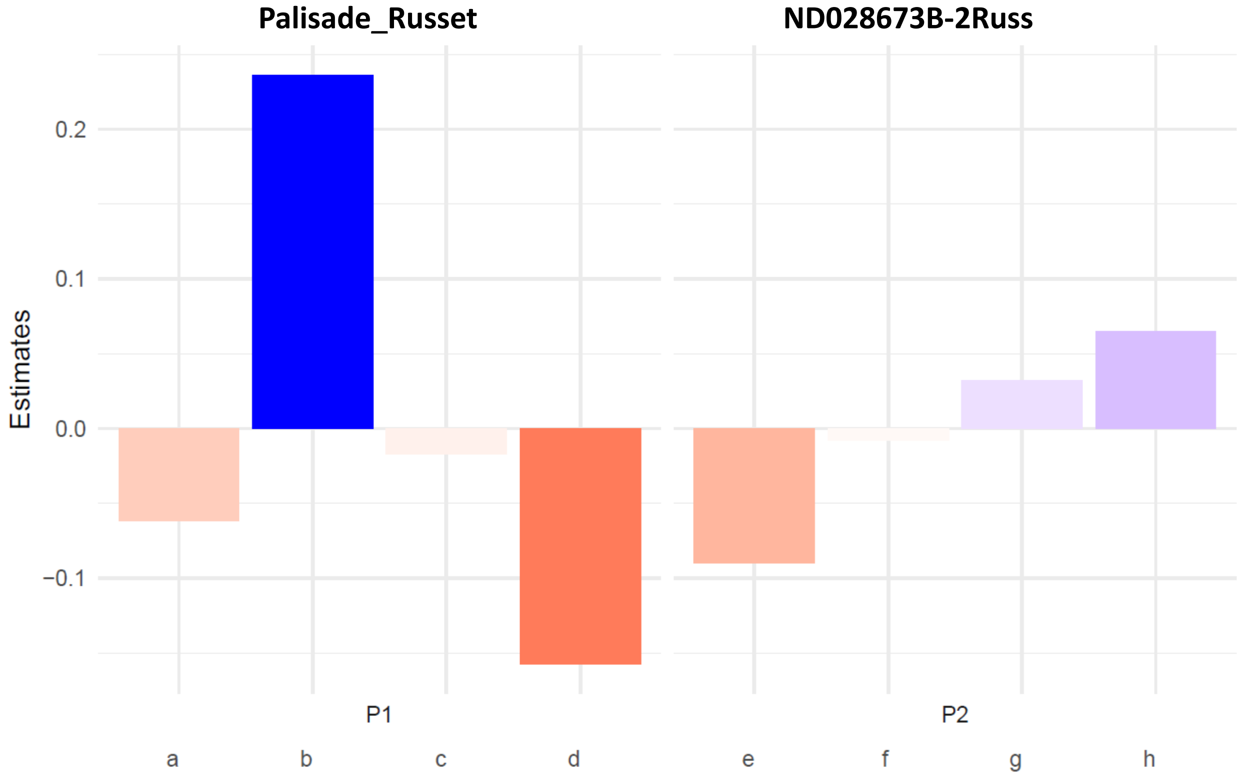

EB_2019_pheno_ch2

SNP (Location): solcap_snp_c2_37254 (19.30 cM)
Chromosome: chr02
BLUP data: *EB_clo_2019*

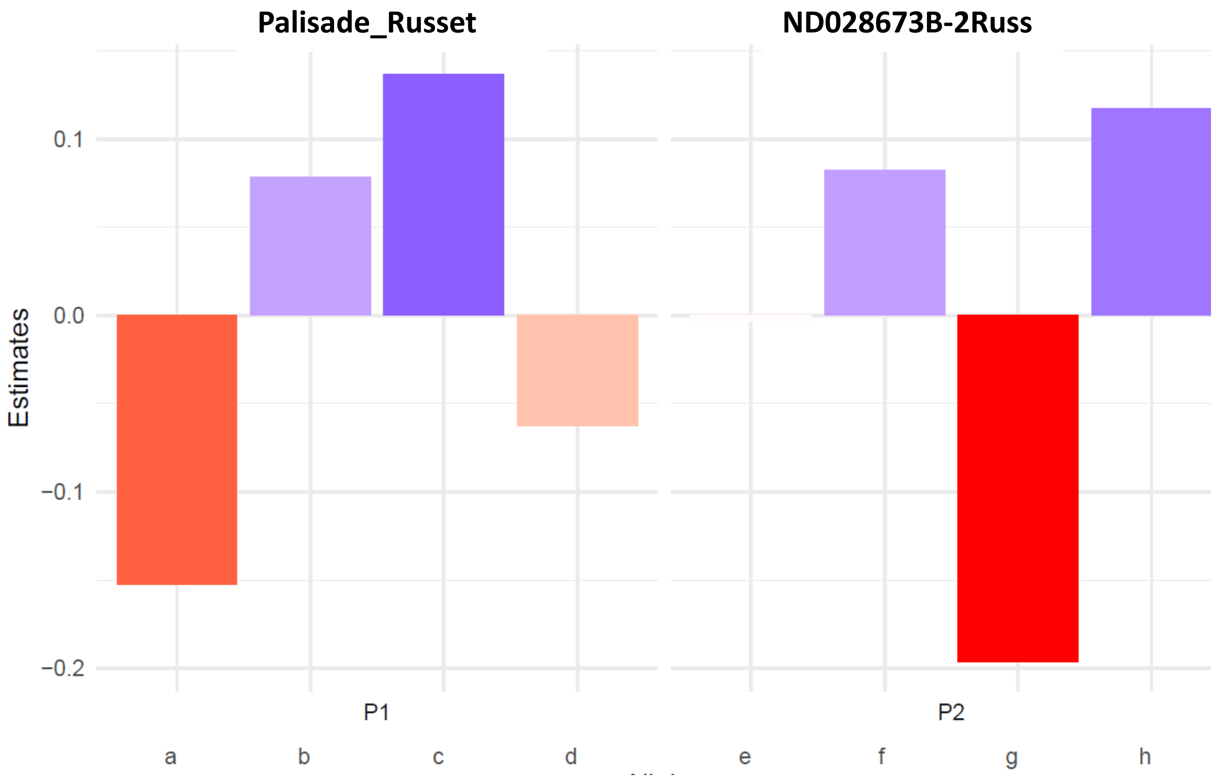

EB_2019_pheno_ch5

SNP (Location): solcap_snp_c2_11961 (14.33 cM)
Chromosome: chr05
BLUP data: *EB_clo_2019*

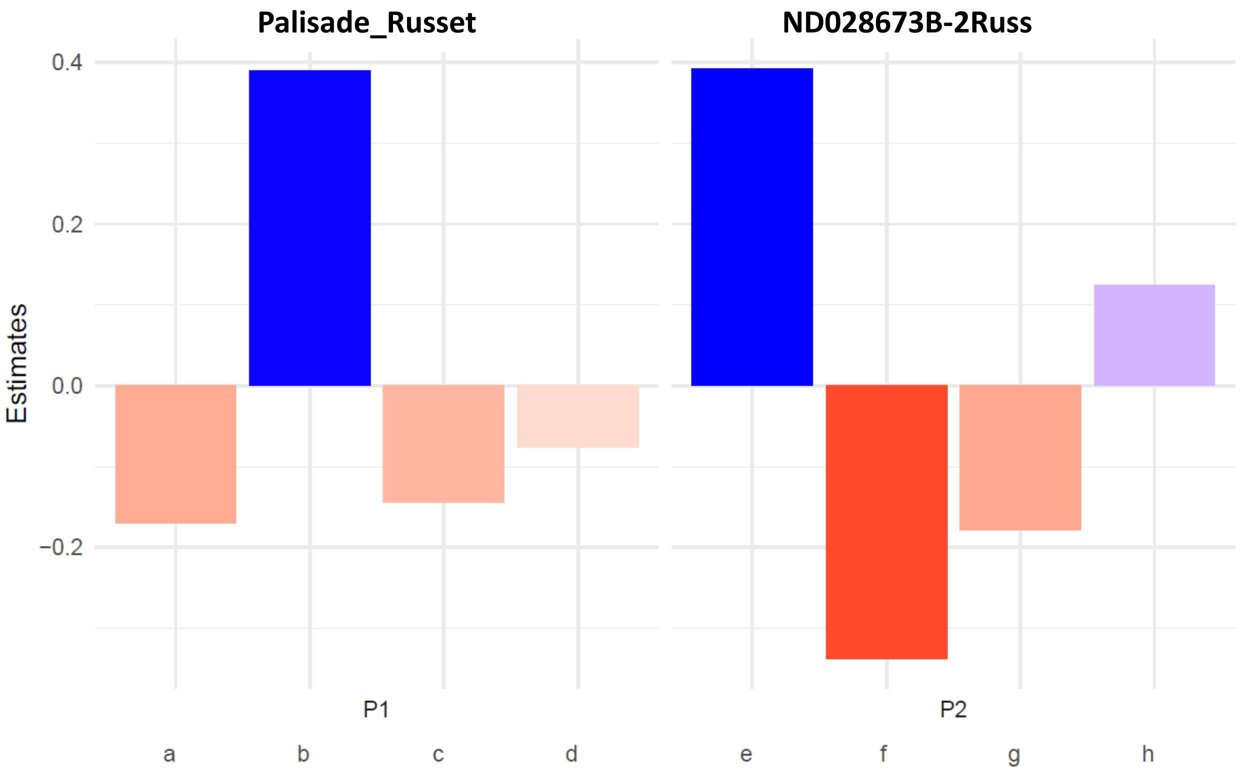

Supplement: Supplementary file 1 [file DataSheet_1.zip › DataSheet_4.pdf]
