# Supplementary material for: Identification of QTL associated with plant vine characteristics and infection response to late blight, early blight, and Verticillium wilt in a tetraploid potato population derived from late blight-resistant Palisade Russet
Source: Front Plant Sci. 2023 Oct 11;14:1222596. doi: 10.3389/fpls.2023.1222596 (PMC10600477; doi:10.3389/fpls.2023.1222596)
Supplement: Supplementary file 1 [file DataSheet_1.zip › DataSheet_5.docx]

**Supplementary Figure 5. Haplotype dissimilarities between late blight resistant and susceptible panels**


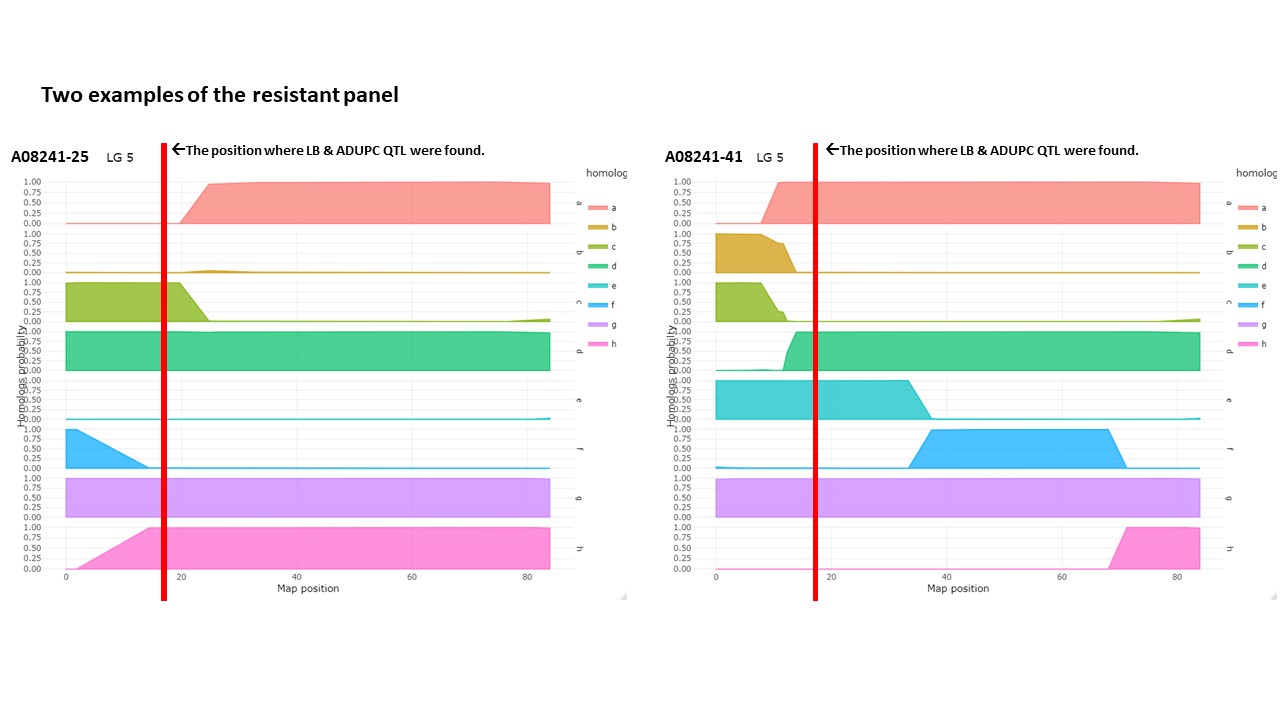


These two figures represented haplotype images of the two examples of the late blight-resistant clones, which were consistently ranked in the top 20% of the resistant panel across the two years. Each horizontal bar represented four homologs (a-d) of Palisade Russet and another four homologs (e-h) of ND028673B-2Russ. The vertical red bars indicated the position (17.09 cM) where significant QTL for LB and LB-AUDPC were found. As described in Supplementary Figure 4, the negative effect allele at 17.09 cM tended to be located on homologs a, c, d, f, and g. On the other hand, the positive effect allele at 17.09 cM tended to be located on homologs b, e, and h. It is also possible to see the place where recombination took place. For example, when the A08241-25 clone was analyzed, recombination between homologs a and c took place near 22 cM. Another recombination between homologs f and h took place.

|  |  |  | |  | |  |  |  |  |  |  |  |  |  |
| --- | --- | --- | --- | --- | --- | --- | --- | --- | --- | --- | --- | --- | --- | --- |
| Resistant haplotype panel list | | | hmlg^p^ | | hmlg^p^ | hmlg^p^ | hmlg^p^ | hmlg^N^ | hmlg^N^ | hmlg^N^ | hmlg^N^ |  |  |  |
| total 17 / homologs a-h | | | a | | b | c | d | e | f | g | h | count m | count p | SUM |
| A08241-2 | | | m | |  | m |  |  | m | m |  | 4 | 0 | -4 |
| A08241-25 | | |  | |  | m | m |  |  | m | p | 3 | 1 | -2 |
| A08241-27 | | |  | |  | m | m |  | m | m |  | 4 | 0 | -4 |
| A08241-37 | | | m | |  | m |  |  | m |  | p | 3 | 1 | -2 |
| A08241-39 | | |  | |  | m | m |  | m | m |  | 4 | 0 | -4 |
| A08241-41 | | | m | |  |  | m | p |  | m |  | 3 | 1 | -2 |
| A08241-54 | | | m | |  |  | m | p |  | m |  | 3 | 1 | -2 |
| A08241-55 | | |  | |  | m | m | p | m |  |  | 3 | 1 | -2 |
| A08241-56 | | | m | |  | m |  |  | m | m |  | 4 | 0 | -4 |
| A08241-75 | | |  | |  | m | m |  | m | m |  | 4 | 0 | -4 |
| A08241-104 | | |  | |  | m | m |  |  | m | p | 3 | 1 | -2 |
| A08241-105 | | |  | |  | m | m |  | m | m |  | 4 | 0 | -4 |
| A08241-114 | | |  | |  | m | m |  | m | m |  | 4 | 0 | -4 |
| A08241-175 | | |  | | p |  | m |  | m | m |  | 3 | 1 | -2 |
| A08241-177 | | | m | |  | m |  |  | m | m |  | 4 | 0 | -4 |
| A08241-179 | | | m | |  | m |  | p | m |  |  | 3 | 1 | -2 |
| A08241-190 | | | m | |  | m |  |  | m | m |  | 4 | 0 | -4 |
| Total effect count | | | 8 | | 1 | 14 | 11 | 4 | 13 | 14 | 3 |  |  |  |

"m" is minus (or negative) effects

"p" is plus (or positive) effects

“hmlg^P”^ represents the four homologs of Palisade Russet.

“hmlg^N”^ represents another four homologs of ND028673B-2Russ.

Before starting to interpret the table above, it should be noted that the *LB-*AUDPC_clo_ch5 allele effect test (Supplementary Figure 4) showed that negative effect alleles were located on homologs a, c, d, f, and g. On the other hand, positive effect alleles were located on homologs b, e, and h.

This table marked which homolog each negative and positive effect allele resides on while analyzing the resistant panel's haplotypes (at 17.09 cM). For instance, if you see the haplotype image of A08241-25 above, alleles were detected on homologs c, d, g, and h at 17.09 cM; thus, we marked colors on the columns, representing homologs c, d, g, and h in the following table. Likewise, the same process was repeated for the other 16 clones to fill in the blanks in the table. After completely filling in those blanks based on the haplotype images, the number of positive and negative effect alleles of each clone was counted, and then the net worth (labeled with "SUM") for each clone was obtained. All the numbers of the positive and negative alleles as well as their sums were indicated on the right side of the table above.


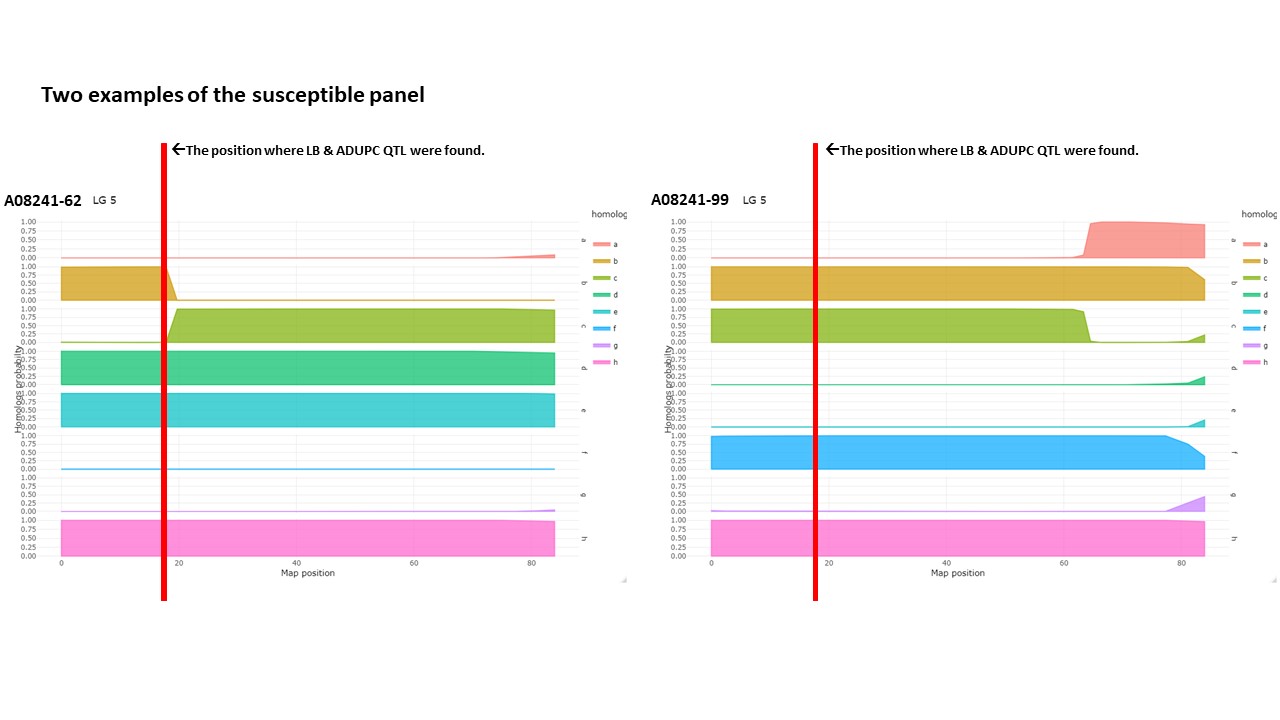


These two figures represented haplotype images of the two examples of the late blight susceptible clones, which were consistently ranked in the top 20% of the susceptible panel across the two years. The rest of the description is the same as above.

|  |  |  | |  | |  |  |  |  |  |  |  |  |  |  |
| --- | --- | --- | --- | --- | --- | --- | --- | --- | --- | --- | --- | --- | --- | --- | --- |
| Susceptible haplotype panel list | | | hmlg^p^ | | hmlg^p^ | | hmlg^p^ | hmlg^p^ | hmlg^N^ | hmlg^N^ | hmlg^N^ | hmlg^N^ |  |  |  |
| total 16 / homologs a-h | | | a | | b | | c | d | e | f | g | h | count m | count p | SUM |
| A08241-16 | | | m | | p | |  |  |  |  | m | p | 2 | 2 | 0 |
| A08241-35 | | |  | | p | |  | m | p |  | m |  | 2 | 2 | 0 |
| A08241-38 | | |  | | p | | m |  | p | m |  |  | 2 | 2 | 0 |
| A08241-45 | | |  | | p | | m |  | p |  | m |  | 2 | 2 | 0 |
| A08241-62 | | |  | | p | |  | m | p |  |  | p | 1 | 3 | 2 |
| A08241-63 | | | m | |  | | m |  | p |  |  | p | 2 | 2 | 0 |
| A08241-64 | | | m | | p | |  |  | p |  | m |  | 2 | 2 | 0 |
| A08241-66 | | | m | | p | |  |  |  | m |  | p | 2 | 2 | 0 |
| A08241-78 | | | m | | p | |  |  | p |  |  | p | 1 | 3 | 2 |
| A08241-85 | | |  | | p | | m |  |  | m |  | p | 2 | 2 | 0 |
| A08241-90 | | |  | | p | | m |  |  | m |  | p | 2 | 2 | 0 |
| A08241-99 | | |  | | p | | m |  |  | m |  | p | 2 | 2 | 0 |
| A08241-149 | | |  | | p | |  | m |  | m |  | p | 2 | 2 | 0 |
| A08241-150 | | |  | | p | |  | m |  | m | m |  | 3 | 1 | -2 |
| A08241-159 | | | m | | p | |  |  |  |  | m | p | 2 | 2 | 0 |
| A08241-185 | | | m | |  | | m |  | p |  |  | p | 2 | 2 | 0 |
| Total effect count | | | 7 | | 14 | | 7 | 4 | 8 | 7 | 6 | 11 |  |  |  |

"m" is minus (or negative) effects

"p" is plus (or positive) effects

“hmlg^P”^ represents the four homologs of Palisade Russet.

“hmlg^N”^ represents another four homologs of ND028673B-2Russ.

This table marked which homolog each negative and positive effect allele resides on while analyzing the susceptible panel's haplotypes (at 17.09 cM). The rest of the description is the same as above.
