# Supplementary material for: Identification of QTL associated with plant vine characteristics and infection response to late blight, early blight, and Verticillium wilt in a tetraploid potato population derived from late blight-resistant Palisade Russet
Source: Front Plant Sci. 2023 Oct 11;14:1222596. doi: 10.3389/fpls.2023.1222596 (PMC10600477; doi:10.3389/fpls.2023.1222596)
Supplement: Supplementary file 1 [file DataSheet_1.zip › Table_2.docx]

**Supplementary Table 2. Disease rating scores for early blight and Verticillium wilt**

| Score | Percent | Descriptions |
| --- | --- | --- |
| 0 | 0 | No symptom of infection. |
| 1 | <1 | Only light trace of the symptom (e.g., spotted color change) is observed. |
| 2 | <5 | More than 1% but less than 5% of the foliage expresses symptoms. |
| 3 | 5-15 | Lesions are easily seen at a close distance. |
| 4 | 15-25 | Up to 25% of the foliage is covered with lesions or destroyed. |
| 5 | 25-40 | Up to 40% of the foliage is covered with lesions or destroyed. |
| 6 | 40-60 | More than half of the foliage is covered with lesions or destroyed. |
| 7 | 60-75 | The majority of the foliage was covered with lesions with only small areas of green visible. |
| 8 | 75-90 | Few green areas remain. |
| 9 | >90 | Foliage was almost completely destroyed. |
