# Supplementary material for: Identification of QTL associated with plant vine characteristics and infection response to late blight, early blight, and Verticillium wilt in a tetraploid potato population derived from late blight-resistant Palisade Russet
Source: Front Plant Sci. 2023 Oct 11;14:1222596. doi: 10.3389/fpls.2023.1222596 (PMC10600477; doi:10.3389/fpls.2023.1222596)
Supplement: Supplementary file 1 [file DataSheet_1.zip › Table_3.docx]

**Supplementary Table 3. Plant vine maturity rating scores**

| Score | Brief description | Detailed descriptions |
| --- | --- | --- |
| 1 | Very early | 100% of foliage dead. |
| 2 |  | 40-70% of foliage dead or brown. |
| 3 |  | 10-30% of foliage dead or brown. |
| 4 | Onset of senescence | Foliage begins to reveal yellow coloration. |
| 5 | Middle | All foliage is green. |
| 6 |  | All foliage is intense green without flowers. |
| 7 |  | All foliage is intense green; few flowers observed. |
| 8 |  | All foliage is intense green; more than ten flowers observed. |
| 9 | Very late | All foliage is intense green; full blooms and new buds observed. |
