# Supplementary material for: Identification of QTL associated with plant vine characteristics and infection response to late blight, early blight, and Verticillium wilt in a tetraploid potato population derived from late blight-resistant Palisade Russet
Source: Front Plant Sci. 2023 Oct 11;14:1222596. doi: 10.3389/fpls.2023.1222596 (PMC10600477; doi:10.3389/fpls.2023.1222596)
Supplement: Supplementary file 1 [file DataSheet_1.zip › Table_4.docx]

**Supplementary Table 4. Plant vine size rating scores**

| Score | Brief description | Detailed descriptions |
| --- | --- | --- |
| 1 | less than 1 foot tall | The plants are less than 1 foot tall. |
| 2 | Between foot and knee | Approximately 12 inches (≈ 1 foot tall). |
| 3 | Knee high | Approximately 19 inches (≈ 1.58 feet tall). |
| 4 | Between knee and waist | Approximately 29 inches (≈ 2.42 feet tall). |
| 5 | Waist high | Approximately 39 inches (≈ 3.25 feet tall). |
| 6 | Between waist and chest | Approximately 45 inches (≈ 3.75 feet tall). |
| 7 | Chest high | Approximately 50 inches (≈ 4.17 feet tall) when vine is pulled upright vertically off the ground. ^a^ |
| 8 | Between chest and neck | Approximately 55 inches (≈ 4.6 feet tall) when vine is pulled upright vertically off the ground. ^a^ |
| 9 | Neck high | Approximately 60 inches (≈ 5 feet tall) or more when vine is pulled upright vertically off the ground. ^a^ |

^a^ The vines of some potato plants lean toward the ground because of their extended length and heavy weight. In those cases, the longest vine was pulled vertically from the ground and measured.
