# Supplementary material for: Identification of QTL associated with plant vine characteristics and infection response to late blight, early blight, and Verticillium wilt in a tetraploid potato population derived from late blight-resistant Palisade Russet
Source: Front Plant Sci. 2023 Oct 11;14:1222596. doi: 10.3389/fpls.2023.1222596 (PMC10600477; doi:10.3389/fpls.2023.1222596)
Supplement: Supplementary file 1 [file DataSheet_1.zip › Table_5.docx]

**Supplementary Table 5. Summary of the BLUP datasets of all the five traits**

| **Late Blight (*LB*) foliage damage** | | | | | | | |  |
| --- | --- | --- | --- | --- | --- | --- | --- | --- |
| BLUP datasets ^a^ |  | Description | |  | Total  BLUPs ^b^ | | | |
| *LB_clo* |  | BLUPs of pooled phenotype data across the two years | |  | 190 | | | |
| *LB_clo_2019* |  | BLUPs of interaction between clone and 2019 (year). | |  | 190 | | | |
| *LB_clo_2020* |  | BLUPs of interaction between clone and 2020 (year). | |  | 190 | | | |
|  | | | | | | | |  |
| **Area Under the Disease Progress Curve (*LB-AUDPC*)** | | | | | | | |  |
| BLUP datasets ^a^ |  | Description | |  | | | Total  BLUPs ^b^ | |
| *LB-AUDPC_clo* |  | BLUPs of pooled phenotype data across the two years |  | | | 190 | |  |
| *LB-AUDPC_clo_2019* |  | BLUPs of interaction between clone and 2019 (year). |  | | | 190 | |  |
| *LB-AUDPC_clo_2020* |  | BLUPs of interaction between clone and 2020 (year). |  | | | 190 | |  |
|  | | | | | | | |  |
| **Verticillium Wilt (*VW*) foliage damage** | | | | | | | |  |
| BLUP datasets ^a^ |  | Description | |  | | | Total  BLUPs ^b^ | |
| *VW_clo* |  | BLUPs of pooled phenotype data across the two years |  | | | 184 | |  |
| *VW_clo_2019* |  | BLUPs of interaction between clone and 2019 (year). |  | | | 184 | |  |
| *VW_clo_2020* |  | BLUPs of interaction between clone and 2020 (year). |  | | | 184 | |  |
|  | | | | | | | |  |
| **Vine Maturity (*VM*)** | | | | | | | |  |
| BLUP datasets ^a^ |  | Description | |  | | | Total  BLUPs ^b^ | |
| *VM_clo* |  | BLUPs of pooled phenotype data across the two years |  | | | 184 | |  |
| *VM_clo_2019* |  | BLUPs of interaction between clone and 2019 (year). |  | | | 184 | |  |
| *VM_clo_2020* |  | BLUPs of interaction between clone and 2020 (year). |  | | | 184 | |  |
|  | | | | | | | |  |
| **Vine Size (*VS*)** | | | | | | | |  |
| BLUP datasets ^a^ |  | Description | |  | | | Total  BLUPs ^b^ | |
| *VS_clo* |  | BLUPs of pooled phenotype data across the two years |  | | | 184 | |  |
| *VS_clo_2019* |  | BLUPs of interaction between clone and 2019 (year). |  | | | 184 | |  |
| *VS_clo_2020* |  | BLUPs of interaction between clone and 2020 (year). |  | | | 184 | |  |

^a^ BLUP data abbreviations: Late Blight Foliage Damage (*LB*), Late Blight Area Under the Disease Progress Curve (*LB-AUDPC*), Verticillium Wilt resistance (*VW*), Vine Maturity (*VM*), Vine Size (*VS*), a genetic effect of clones (*clo*), 2019 (*2019*), and 2020 (*2020*) year effects

Unlike the other five traits, no BLUP data information for *EB* was included in Supplementary Table 5 because the average values of two replicates of 2019 raw data for early blight resistance phenotype (*EB_2019_raw_pheno*) were used in this study,

^b^ Some of the BLUP datasets had slightly different numbers of components due to several missing values during the field experiments.
