# Supplementary material for: Identification of QTL associated with plant vine characteristics and infection response to late blight, early blight, and Verticillium wilt in a tetraploid potato population derived from late blight-resistant Palisade Russet
Source: Front Plant Sci. 2023 Oct 11;14:1222596. doi: 10.3389/fpls.2023.1222596 (PMC10600477; doi:10.3389/fpls.2023.1222596)
Supplement: Supplementary file 1 [file DataSheet_1.zip › Table_8.docx]

**Supplementary Table 8. Location comparison of the mapped QTL and linked SNPs between the A08241 mapping population and previously published references on molecular markers associated with the significant QTL**

|  | Information obtained in current study (A08241_QTL analyses) | | | | Information obtained from references | | | |
| --- | --- | --- | --- | --- | --- | --- | --- | --- |
| **Trait categories** | QTL ^a^ (Traits) | Ch | SNPs ^b^ | Phy. Map. Pos. ^c^ | R Gene or Molecular markers from references ^d^ | References | Reference  Marker phy. Map. Pos. ^e^ | Dist. Bet. Our SNP and reference SNP (cM) ^f^ |
| Late Blight | ***LB_clo_ch5***  ***(LB_clo)***  ***LB_clo_2019_ch5***  ***(LB_clo_2019)***  ***LB_clo_2020_ch5***  ***(LB_clo_2020)***  ***LB-AUDPC_clo_ch5***  ***(LB-AUDPC_clo)***  ***LB-AUDPC_clo_2019_ch5 (LB-AUDPC_clo_2019)***  ***LB-AUDPC_clo_2020_ch5***  ***(LB-AUDPC_clo_2020)*** | 5 | PotVar0077880 | 4401848 | R1  (R Gene) | Ballvora et al. 2002; Bormann et al. 2004; Gebhardt et al. 2004 | 5211400  - 5352950 | 0.81 |
|  |  |  |  |  | GP21 (RFLP) | Leonards-Schippers et al. 1994;  Van Eck and Jacobsen 1996;  Collins et al. 1999; Oberhagemann et al. 1999;  Visker et al. 2003 | 4034000 - 4042300 | 0.36 |
|  |  |  |  |  | GP179  (RFLP) | Leonards-Schippers et al. 1994;  Collins et al. 1999; Oberhagemann et al. 1999;  Bormann et al. 2004 | 5946200  - 5952500 | 1.54 |
|  |  |  |  |  | STM0013  (SSR) | Collins et al. 1999; Ghislain et al. 2001 | 2586070  - 2586177 | 1.82 |
|  |  |  |  |  | BA121o1t7  (BAC clone) | Bormann et al. 2004 | 5189200  - 5193000 | 0.79 |
|  |  |  |  |  | STM3179  (SSR) | Bradshaw et al. 2004 | 6035018  - 6035174 | 1.63 |
| Verticillium wilt | ***VW_clo_ch5***  ***(VW_clo)***  ***VW_clo_2019_ch5***  ***(VW_clo_2019)***  ***VW_clo_2020_ch5***  ***(VW_clo_2020)*** | 5 | PotVar0026113 | 4250232 | solcap_snp_c2_11605  (SNP) | Massa et al. 2018 | 1957512 | 2.29 |
|  |  |  |  |  |  |  |  |  |
| Vine Maturity | ***VM_clo_ch5***  ***(VM_clo)***  ***VM_clo_2019_ch5***  ***(VM_clo_2019)***  ***VM_clo_2020_ch5***  ***(VM_clo_2020)*** | 5 | PotVar0026113 | 4250232 | GP21 (RFLP) | Collins et al. 1999  Visker et al. 2003 | 4034000 - 4042300 | 0.21 |
|  |  |  |  |  | GP179  (RFLP) | Collins et al. 1999 | 5946200  - 5952500 | 1.70 |
|  |  |  |  |  | STM3179  (SSR) | Bradshaw et al. 2004 | 6035018  - 6035174 | 1.78 |
|  |  |  |  |  | solcap_snp_c2_47609  (SNP) | Hackett et al. 2014 | 5972404 | 1.72 |
|  |  |  |  |  | solcap_snp_c2_11605  (SNP) | Massa et al. 2018 | 1957512 | 2.29 |
|  |  |  |  |  | StCDF1  (Gene) | Navarro et al., 2011; González-Schain et al., 2012;  Kloosterman et al., 2013;  Salaria et al. 2020 | 4538880  - 4541730 | 0.29 |
|  |  |  |  |  | PotVar0079081 | Klaassen et al. 2019 Willemsen 2018 | 4489481 | 0.24 |
| Vine Size | ***VS_clo_ch5***  ***(VS_clo)***  ***VS_clo_2020_ch5***  ***(VS_clo_2020)*** | 5 | PotVar0077880 | 4401848 | solcap_snp_c2_47609  (SNP) | Hackett et al. 2014 | 5972404 | 1.57 |
|  | ***VS_clo_2019_ch5***  ***(VS_clo_2019)*** |  | PotVar0026113 | 4250232 | solcap_snp_c2_47609  (SNP) | Hackett et al. 2014 | 5972404 | 1.72 |
| Early Blight | ***EB_2019_pheno_ch5***  ***(EB_2019_raw_pheno)*** | 5 | solcap_snp_c2_11961 | 3538675 | PotVar0026113  (SNP) | Odilbekov et al. 2020 | 4250232 | 0.71 |
|  |  |  |  |  | PotVar0079374  (SNP) | Odilbekov et al. 2020 | 4495794 | 0.96 |

All the physical map location information of the molecular markers presented in Supplementary Table 8 was obtained from the potato reference genome PGSC Version 4.03

^a^ Titles of the mapped QTL and BLUP datasets

^b^ SNPs linked to the mapped significant QTL in this study

^c^ Physical map positions of those SNPs

^d^ Molecular markers linked to gene(s) or QTL associated with late blight, early blight, Verticillium wilt resistances, as well as vine size and maturity identified by reference researchers

^e^ Physical map positions of those molecular markers identified by reference researchers

^f^ Distance between SNPs identified in this study and the molecular markers studied by reference researchers
