# Supplementary material for: Identification of QTL associated with plant vine characteristics and infection response to late blight, early blight, and Verticillium wilt in a tetraploid potato population derived from late blight-resistant Palisade Russet
Source: Front Plant Sci. 2023 Oct 11;14:1222596. doi: 10.3389/fpls.2023.1222596 (PMC10600477; doi:10.3389/fpls.2023.1222596)
Supplement: Supplementary file 1 [file DataSheet_1.zip › Table_1.docx]

**Supplementary Table 1. Late blight field reading scores**

| Score | Percent | Descriptions |
| --- | --- | --- |
| 1 | 1 | No symptom of infection or very low number of lesions within the row. |
| 2 | 3 | More than 0% but less than 10%. |
| 3 | 10 | Lesions are easily seen at a close distance. |
| 4 | 25 | About 25% of the foliage is covered with lesions or destroyed. |
| 5 | 50 | Half of the foliage was destroyed. |
| 6 | 75 | 75% of each plant is affected. |
| 7 | 90 | Only top leaves are green. |
| 8 | 97 | Very few green areas remain. |
| 9 | 100 | Foliage completely destroyed. |
