# Supplementary material for: Identification of QTL associated with plant vine characteristics and infection response to late blight, early blight, and Verticillium wilt in a tetraploid potato population derived from late blight-resistant Palisade Russet
Source: Front Plant Sci. 2023 Oct 11;14:1222596. doi: 10.3389/fpls.2023.1222596 (PMC10600477; doi:10.3389/fpls.2023.1222596)
Supplement: Supplementary file 1 [file DataSheet_1.zip › DataSheet_6.doc]

**Supplementary Material 1. Protocol of the modified rye A media for late blight (*Phytophthora infestans*) increase.**

1. Soak 100g of rye grain (use organic) in distilled water (~400ml) for 24 hrs at room temperature.
2. Place grain & soaking water in a blender and coarsely chop grain (1 pulsate for ~15 sec)
3. Heat in microwave for 2-3 minutes on high (don’t boil).
   Let stand for 30 min.
4. Filter through 4-layers of cheesecloth.
   Discard the cheesecloth and grain sediment
5. Bring liquid volume to one liter with distilled water.
   Add 20g Sucrose & 15 g Bacto agar.
6. Autoclave at 15 psi for 20 minutes.
7. Pour into plates.
   One liter will yield 40-50, 100x15 mm petri plates.
